# A mitochondrion–targeted natural polyphenolic copper carrier overcomes tumor resistance to cisplatin by potentiating cuproptosis

**DOI:** 10.1186/s13046-026-03642-5

**Published:** 2026-01-20

**Authors:** Haoyu Yang, Xiang Xiong, Xin Chen, Siqi Huang, Hongfang Dai, Liqin Yuan, Jialong Fan, Zhenhong Xiang, Wei Wang, Yan Qin

**Affiliations:** 1 School of Pharmacy, Hunan University of Chinese Medicine, Changsha, Hunan 410208 P. R. China; 2 The Second Xiangya Hospital, Central South University, Changsha, Hunan 410011 P. R. China; 3 Hunan Provincial Key Laboratory of the Research and Development of Novel Pharmaceutical Preparation, Changsha Medical University, Changsha, China

**Keywords:** Drug resistance, Cuproptosis, Glutathione (GSH), Mitochondrial copper, ATPase (ATP7A/B)

## Abstract

**Background:**

Platinum–based drug resistance remains a major obstacle in cancer therapy. Cuproptosis, a novel form of copper–dependent cell death regulated through mitochondrial pathways, represents a promising strategy to counteract drug resistance in tumors. However, its efficacy is constrained by several physiological barriers, including elevated intracellular glutathione (GSH) levels, inadequate copper accumulation both cytoplasmically and within mitochondria, and the overexpression of copper efflux transporters such as ATP7A/B.

**Methods:**

A series of material characterization techniques (FT-IR, UV-vis, XPS, ICP-MS, XRD, DLS, TEM) were employed to systematically characterize bCCM. At the cellular level, experiments including CCK-8 assay, live/dead staining via holographic microscopy, apoptosis/necrosis staining imaging, ROS staining imaging, copper ion imaging, mitochondrial co-localization imaging, mitochondrial membrane potential imaging, and JC-1 staining were performed on cisplatin-resistant hepatocellular carcinoma cells BEL7402/DDP. In animal models, further studies were conducted, such as small animal in vivo imaging, tissue copper content measurement, TUNEL assay, and immunohistochemistry (IHC).

**Results:**

We have developed a mitochondria-targeted polyphenol-copper nanocarrier, bCCM, which operates through three synergistic mechanisms to enhance the efficacy of cisplatin-resistant hepatocellular carcinoma (HCC) therapy. First, it significantly increases intracellular copper delivery through high-capacity tridentate chelation while depleting glutathione (GSH) to prevent the formation of inert GSH-Cu/Pt complexes, thereby improving the bioavailability of both copper and cisplatin. Second, it promotes mitochondrial copper accumulation via targeted delivery and localized GSH depletion, leading to irreversible mitochondrial damage. Third, it downregulates ATP7B expression, inhibiting the efflux of copper and cisplatin, which further enhances intracellular copper retention and chemosensitivity. Both in vitro and in vivo evaluations demonstrate that bCCM effectively targets tumor cells, exerts potent antitumor activity against cisplatin-resistant HCC, and does not induce systemic toxicity or adverse copper accumulation.

**Conclusions:**

bCCM downregulates key proteins associated with cuproptosis and cisplatin resistance, demonstrating effective synergy between cuproptosis and conventional chemotherapy. This study establishes bCCM as an innovative therapeutic platform for overcoming platinum-based chemotherapy resistance, showing significant potential for clinical translation in oncology.

**Supplementary Information:**

The online version contains supplementary material available at 10.1186/s13046-026-03642-5.

## Introduction

Platinum–based drugs, exemplified by cisplatin (DDP), constitute a cornerstone of cancer chemotherapy. However, their clinical utility is substantially limited by the emergence of drug resistance [1]. Prolonged or repeated drug administration has been shown to induce a multi–mechanistic synergistic resistance phenotype characterized primarily by two interrelated processes. First, membrane transporter–mediated drug efflux, driven by the overexpression of P–glycoprotein (P–gp) and copper–transporting ATPase (ATP7B), which significantly diminishes intracellular drug accumulation [2–4]. Second, metabolic inactivation of drugs through conjugation with endogenous molecules such as glutathione (GSH)[5, 6]. These mechanisms often coexist and reinforce one another, forming a complex resistance network. Conventional strategies such as dose escalation or the use of more cytotoxic agents not only fail to overcome this network but also may exacerbate adverse effects[7, 8]. Consequently, the development of innovative therapeutic strategies that effectively reverse resistance while maintaining a favorable safety profile represents a critical challenge in contemporary oncology research.

Cuproptosis, a novel form of regulated cell death first described by Tsvetkov et al. in 2022 [9], is triggered by copper overload–induced mitochondrial dysfunction [9–11] and offers promising potential for overcoming tumor resistance to conventional therapies [12–14]. Specifically, enriched intracellular copper ions within cells directly bind to lipoylated components of the mitochondrial tricarboxylic acid (TCA) cycle[9], leading to the aggregation of lipoylated proteins, particularly dihydrolipoamide S–acetyltransferase (DLAT) and the loss of iron–sulfur (Fe–S) cluster proteins, including ferredoxin 1 (FDX1) and lipoic acid synthetase (LIAS)[15, 16]. These events induce proteotoxic stress and ultimately result in cell death. The mechanism of copper involvement involves two key aspects. FDX1 and LIAS mediate the lipoylation of DLAT, enabling copper to promote its oligomerization, and copper downregulates Fe–S cluster proteins. Together, these processes culminate in cuproptosis[17, 18]. Notably, not all copper ions are equally effective, cuprous ions (Cu⁺) exhibit greater toxicity than cupric ions (Cu²⁺) because of their superior ability to bind lipoylated TCA cycle proteins[19]. Therefore, to induce cuproptosis, Cu²⁺ must first be reduced to Cu⁺, dissociate from ion carriers, and subsequently bind lipoic acid moieties on DLAT to trigger aggregation. Thus, enhancing both copper accumulation and its reduction to Cu⁺ represents a crucial strategy for activating cuproptosis in resistant tumors.

However, the therapeutic application of cuproptosis is limited by several obstacles. Simply elevating copper levels is often insufficient to trigger effective cuproptosis within the complex tumor microenvironment. First, GSH level elevation chelates copper ions, which is common in drug–resistant cells, reducing the amount of bioavailable copper and impeding cuproptosis[20–24]. Second, copper efflux is mediated by copper–transporting ATPases (ATP7A/B), which expel excess copper to maintain homeostasis[25, 26]. These same mechanisms also contribute to resistance against chemotherapeutic agents such as cisplatin via GSH–mediated inactivation and ATP7A/B–dependent efflux. Current strategies to increase the level of intracellular copper include enhancing copper uptake, minimizing copper loss, and inhibiting copper export. Several copper ionophores, such as elesclomol (ES), dithiocarbamate, bis(thiosemicarbazone) ligands, 8–hydroxyquinolines, pyrithione, and disulfiram have been explored for their ability to promote copper accumulation and cuproptosis[27–33]. Among these, ES uniquely delivers copper to mitochondria, facilitating sustained copper accumulation[34]. Nonetheless, clinical trials of ES have revealed limited efficacy due to rapid systemic clearance and metabolism[35]. Other ionophores face similar translational challenges, including poor biosafety, lack of targeting, or insufficient cuproptosis induction[36]. Thus, there is an urgent need to develop safe and efficient copper delivery systems with high loading capacity, GSH depletion ability, and ATPase inhibitory activity.

In this study, we designed and synthesized a novel multifunctional copper carrier, bisdemethylcurcumin (bm–Cur), which contains multiple metal–chelating groups (adjacent carbonyl and phenolic hydroxyl moieties) capable of coordinating Cu²⁺ at three distinct sites. bm–Cur also depletes GSH via Michael addition, which is mediated by its α, β–unsaturated ketone structures[37–40], and inhibits the cystine/glutamate antiporter (xCT), thereby disrupting GSH synthesis[41, 42]. Additionally, bm–Cur facilitates mitochondrial copper delivery, induces mitochondrial damage, and demonstrates selectivity toward cancer cells over normal hepatocytes[43]. It also downregulates ATP7B expression, further inhibiting copper efflux. We therefore hypothesized that bm–Cur could serve as an ideal multifunctional copper ionophore, enhancing copper delivery while simultaneously depleting GSH and suppressing ATP7B–mediated export to potentiate cuproptosis.

**Scheme 1:**
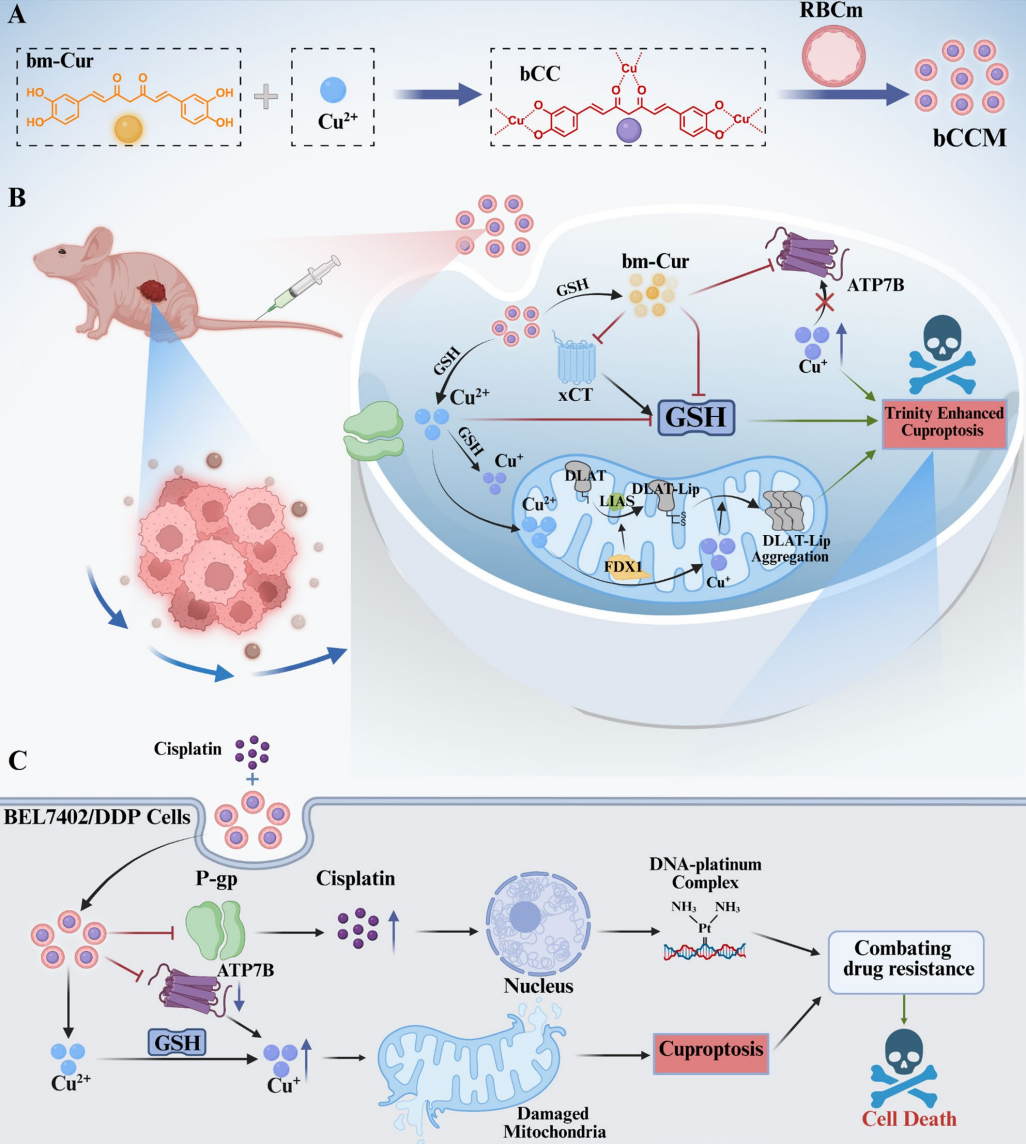
Schematic illustration of bCCM preparation and its mechanisms for potentiating cuproptosis in drug–resistant cancer therapy. (**A**) Preparation of bCCM. (**B**) Detailed mechanism by which bCCM potentiates cuproptosis. (**C**) Cuproptosis sensitizes BEL7402/DDP cells to cisplatin to overcome drug resistance

Leveraging these properties, we complexed bm–Cur with Cu (II) and encapsulated it within red blood cell (RBCm) membranes to form a nanocomplex designated bm–Cur–Cu (II)@RBCm (bCCM) (Scheme 1A). Upon cellular internalization, bCCM releases both copper and bm–Cur, initiating a cascade of therapeutic effects: Cu²⁺ is reduced to Cu⁺ via a Fenton–like reaction involving GSH, and the resulting Cu⁺ binds lipoylated DLAT to trigger cuproptosis. Concurrently, bm–Cur amplifies this process by depleting GSH (via Michael addition) and inhibiting xCT while also downregulating ATP7B to reduce copper export. This self–reinforcing cycle enhances copper retention and promotes cuproptosis in cisplatin–resistant hepatocellular carcinoma (HCC) cells (Scheme 1B). Moreover, bm–Cur and bCCM specifically target mitochondria, increase mitochondrial copper levels, deplete mitochondrial GSH, and induce mitochondrial dysfunction[44–46], significantly augmenting cuproptosis and reversing cisplatin resistance (Scheme 1C). Transcriptomic analyses further supported the association between bCCM treatment and cuproptosis activation. Both in vitro and in vivo, bCCM exhibited potent anti–HCC activity without harming normal tissues, downregulating the expression of cuproptosis–related proteins (FDX1, DLAT, ATP7B, and LIAS) and resistance markers (ATP7B and P–gp). Importantly, bCCM demonstrated favorable pharmacokinetics, high tumor accumulation, minimal off–target retention, and excellent biocompatibility, underscoring its clinical translational potential.

## Materials and methods

### Materials and reagents

GSH was purchased from Shanghai Yuanye Biotechnology Co. Copper perchlorate hexahydrate was purchased from Sinopharm (Beijing, China). Dimethyl sulfoxide (DMSO) and cisplatin were purchased from Shanghai McLean Biochemical Technology Co. cis-Diaminodichloroplatinum (DDP) and Tetrathiomolybdate (TTM) were purchased from MedChemExpress. Phosphate buffer solution (PBS), fetal bovine serum (FBS), Duchenne–modified Eagle’s medium (DMEM), trypsin, and methylthiazolium tetrazolium (MTT) were purchased from Procell Life Science and Technology Co. Ltd. (Wuhan, China). The Annexin V–FITC Apoptosis Detection Kit and BCA Protein Detection Kit were purchased from Beijing Sola Bio–technology Co. Reactive oxygen species (ROS) assay kits, GSH and GSSG assay kits, ATP assay kits, cellular mitochondrial isolation kits, 4% paraformaldehyde (PFA), Hoechst 33342, 4’,6–diamidino–2–phenylindole (DAPI), and radioimmunoprecipitation (RIPA) lysates were purchased from Biyuntian (Nanjing) Bio–Technology Co. β–Actin (1:2000), FDX1 polyclonal antibodies, ATP7B polyclonal antibodies, DLAT polyclonal antibodies and LIAS polyclonal antibodies were purchased from Wuhan Protein Technology Co. xCT polyclonal antibodies were purchased from Abcam plc (Cambridge, UK). The Cellular Cuprous Fluorometric Assay Kit, Cell Copper (Cu^2+^) Colorimetric Assay Kit (Complexing Method), Copper (Cu^2+^) Colorimetric Assay Kit (Complexing Method), terminal deoxynucleotidyl transferase–mediated nick end labeling (Complexing Method), terminal deoxynucleotidyl transferase–mediated nick end labeling (TUNEL) and 3,3’–diaminobenzidine (DAB) staining were purchased from Elabscience Biotechnology Co. (1E, 6E)–1,7–Bis (3,4–dihydroxyphenyl)–1,6–heptadiene–3,5–dione) synthesized from curcumin was named bm–Cur. bm–Cur was obtained from the Laboratory of Chinese Medicine Activity Screening and Eugenics, Hunan University of Traditional Chinese Medicine. HepG2 cells were purchased from Fuheng Biological Company, Shanghai, China (RRID: CVCL_0027). BEL7402/DDP and A2780/DDP cells were purchased from Meixuan Biological Company, Shanghai, China. Hepa1–6 (RRID: CVCL_0327) and HepG2/DDP cells were purchased from Procell Life Science and Technology Co., Ltd. The BEL7402/DDP, A2780/DDP, and HepG2/DDP cell lines are all cisplatin (DDP)–resistant. DDP–induced cisplatin–resistant cell lines were established in vitro and verified via the CCK–8 method.

### Apparatus

UV–vis spectra were measured on a UV–1800 UV spectrophotometer (Shimadzu Corporation, Tokyo, Japan). Inductively coupled plasma–Mass Spectrometry (ICP–MS) measurement was carried out Agilent 8900 Triple Quadrupole Inductively Coupled Plasma Mass Spectrometer (Agilent Technologies, Santa Clara, America). Dynamic light scattering (DLS) measurement was carried out on BeNano 90 Zeta Nano Particle Size and Zeta Potential Analyzer (Bettersize Instruments Ltd., Dandong, China). Fluorescence imaging was performed on a confocal microscope (FV 1200, objective lenses: 60× and 20×) (Olympus Corporation, Tokyo, Japan). Apoptosis analysis was performed on a flow cytometer (Shenzhen Dakovi Biotechnology Co., Ltd.). Mass spectrometry (MS) was performed with a liquid mass spectrometer (Thermo Fisher Scientific, Rockford, IL, USA). Cellular copper ion assays, tissue copper ion assays, cytosolic cuprous ion assays, MTT assays, intracellular ATP content assays, and intracellular GSH content assays were performed with a microplate detector (PerkinElmer Inc., Waltham, Massachusetts, USA). The samples for fluorescence measurements were reacted in a 37 °C water bath. All plastic products used in the cell chambers were autoclaved. All the cells were cultured in a carbon dioxide incubator (HERA CELL 150i) (Thermo Fisher Scientific, Rockford, IL, USA). All the cell experiments were performed in a biosafety cabinet (Thermo Fisher Scientific, Rockford, IL, USA).

### Synthesis of bCCM

Five milliliters of 10 mM copper perchlorate solution was added dropwise to 5 mL of 5 mM bm–Cur solution, Tris–HCl 8.8 was added to adjust the pH to 7.4, the mixture was stirred for 2 h, the mixture was observed to turn black, 10 mL of Erythrocyte membrane suspension were added, and the mixture was stirred for 1 h. bCCM was obtained.

### Spectroscopic measurements

The bCCM stock solution was diluted with distilled water, and GSH was prepared with distilled water. The bm–Cur solutions used for spectroscopic measurements were diluted with distilled water to the final concentration, and GSH was diluted to various concentrations. The test solutions were reacted for 120 min at room temperature, and all the spectroscopic experiments were carried out at room temperature. UV spectra were recorded after the addition of various analytes. The emission wavelengths ranged from 350 nm to 450 nm.

### ICP–MS

ICP–MS was applied to evaluate copper and intracellular platinum content. Glass vials with teflon septa were cleaned with 65% nitric acid, rinsed with ultrapure water, and dried. Cells were collected, rinsed twice with 1× PBS, quantified using an Entek automated counter, and distributed into prepared vials containing 200 µL of either 1× PBS or ultrapure water. Background subtraction used the same volumes in separate vials, duplicated per experiment. Following lyophilization using a CHRIST 2–4 L D plus system, samples were combined with 65% nitric acid and heated at 80 °C overnight. After cooling, they were diluted to 0.475 N with ultrapure water and prepared for mass spectrometry in metal–free centrifuge vials (VWR). Metal quantification was conducted using an Agilent 7900 ICP–QMS in low–resolution mode. Isotope accuracy was maintained with a Micro Mist micro–nebulizer and a Scott spray chamber, employing a helium collision–reaction interface to remove interferences. Scandium and indium were used as internal standards to counteract signal drift and matrix effects. Standards spanning sample concentrations normalized cell count data.

### DLS measurement

A filtered sample (bCC or bCCM) are loaded into a temperature–controlled cuvette. After equilibration, a laser illuminates it, and a detector measures the fluctuations in scattered light intensity over time. The instrument analyzes these fluctuations by computing an intensity autocorrelation function. The decay rate of this function is analyzed to determine the particles’ diffusion coefficient, which is then used to calculate the hydrodynamic size distribution via the Stokes–Einstein equation.

### Stability half-life

The sample was diluted at the same ratio in PBS and serum, then placed simultaneously in a constant temperature and humidity chamber. The UV absorption spectrum was measured hourly, and the time at which a noticeable change in the UV absorption peak occurred was recorded at 40 °C, 50 °C, and 60 °C, respectively. The Arrhenius equation was then used to calculate the stability half-life at room temperature.

### Cell culture

BEL7402/DDP, HepG2/DDP, HepG2 and Hepa1–6 cells were cultured in Dulbecco’s modified Eagle’s medium (DMEM) (Gibco; Thermo Fisher Scientific, USA) supplemented with 10% fetal bovine serum (Procell Life Science and Technology Co., Ltd., China) and 100 U/mL/1% streptomycin in an incubator at 37 °C with 5% humidity. A2780/DDP cells were cultured in Roswell Park Memorial Institute medium 1640 (RPMI–1640) (Gibco; Thermo Fisher Scientific, USA) containing 10% fetal bovine serum (Procell Life Science and Technology Co., Ltd., China) and 100 U/mL/1% streptomycin in an incubator at 37 °C with 5% humidity, where BEL7402/DDP was incubated with a cisplatin concentration of 200 nM, HepG2/DDP with 1 µM and A2780/DDP with 500 nM. The human normal liver cell LX–2 was cultured in Dulbecco’s Modified Eagle Medium/Nutrient Mixture F–12 (DMEM/F12) (Gibco; Thermo Fisher Scientific, USA) supplemented with 10% fetal bovine serum (Procell Life Science and Technology Co., Ltd., China) and 100 U/mL/1% streptomycin in an incubator at 37 °C with 5% humidity.

### CCK–8 assay

BEL7402/DDP cells were inoculated into 96–well plates (8 × 10^3^ cells/well) and incubated in cell culture medium (10% FBS) with different concentrations of bCCM (0–60 µg/mL) and DDP (0–6 µg/mL) for 24 h. Then, CCK–8 solution was added to each well, and the mixture was incubated for 1 h. The cells were then subjected to a CCK–8 assay at 450 nm in a microtiter plate.

### YO–PRO–1/PI apoptosis and necrosis assay

BEL7402/DDP cells (5 × 10^5^ cells/well) were inoculated in six–well plates and incubated with 1 mL of YO–PRO–1/PI test working solution for 20 min after 12 h of treatment with DDP (3 µg/mL), bCCM (60 µg/mL), or bCCM (60 µg/mL) + DDP (1.5 µg/mL), and red and green fluorescence was observed at the end of the incubation period via confocal laser scanning microscopy.

### Cellular copper ion imaging and detection

BEL7402/DDP cells (5 × 10^5^ cells/well) were inoculated in six–well plates and incubated with rhodamine B hydrazide (20 µM) for 30 min after 6 h of treatment with Cu^2+^ (6.4 µg/mL), DDP (3 µg/mL), bCCM (60 µg/mL), or bCCM (60 µg/mL) + DDP (1.5 µg/mL), and red fluorescence was observed at the end of the incubation via confocal laser scanning microscopy. For the copper ion assay, BEL7402/DDP cells were inoculated in six–well plates, and after 24 h of different treatments, the cells were collected, supplemented with 0.2 mL of lysate, lysed on ice for 10 min, and then centrifuged for 10 min at 4℃ and 12,000 × g. The supernatant was collected for the assay, and some of the supernatant was retained for use in the protein concentration assay. A total of 100 µL of standard or sample to be tested was added to the bottom of each well, and 50 µL of color working solution was added. The membrane was covered, incubated at 37 °C for 5 min, and the OD value of each well was detected at 580 nm. Copper ion content (µmol/gprot) = (ΔA580 – standard curve intercept) ÷ standard curve slope × sample dilution ÷ sample protein concentration.

### ROS assay and live/dead staining

The cells (1 × 10^6^ cells/well) were cultured in 6–well plates, treated with DDP (3 µg/mL), bCCM (60 µg/mL), or bCCM (60 µg/mL) + DDP (1.5 µg/mL) for 6 h, incubated with DCFH–DA reagent for 30 min, and then immediately visualized under a confocal laser scanning microscope. Live/dead cell staining: Cells (1 × 10^6^ cells/well) were cultured in 6–well plates, treated with DDP (3 µg/mL), bCCM (60 µg/mL), or bCCM (60 µg/mL) + DDP (1.5 µg/mL) for 6 h, incubated with an appropriate volume of calcein AM/PI assay working solution for 30 min, and then observed under a confocal laser scanning microscope.

### Intracellular GSH assay

The cells (1 × 10^6^ cells/well) were cultured in 6–well plates with DDP (3 µg/mL), bCCM (60 µg/mL), or bCCM (60 µg/mL) + DDP (1.5 µg/mL) for 24 h, after which the cells were harvested. Three times the volume of the cellular precipitate was added to Protein Removal Reagent M solution (GSH and GSSG Assay Kit purchased from Beyotime Institute of Biotechnology, China). If the volume of the cellular precipitate was 10 µl, 30 µl of protein removal reagent M solution was added, and the mixture was vortexed thoroughly. The volume of the cellular precipitate can be estimated from the weight of the precipitate. The centrifuge tubes were weighed before and after collection of the cells so that the weight of the cellular precipitate could be calculated. A total of 10 mg of cellular precipitate can be roughly viewed as 10 µL. The samples were then subjected to two rapid freeze–thaw cycles via liquid nitrogen and a water bath at 37 °C. The samples were left at 4 °C or in an ice bath for 5 min and centrifuged at 4 °C for 10 min at 10,000 × g. The supernatant was used for GSH testing.

### Intracellular ATP assay

The cells (1 × 10^6^ cells/well) were cultured in 6–well plates containing Cisplatin (3 µg/mL), bCCM (60 µg/mL), or bCCM (60 µg/mL) + Cisplatin (1.5 µg/mL) for 24 h, and the cells were lysed by adding lysis buffer at a ratio of 200 µL of lysate per well to a 6–well plate. To sufficiently lyse the cells, a pipette can be used to repeatedly blow or shake the plate so that the lysate can fully contact and lyse the cells. The cells will be lysed immediately after contact with the lysate. After lysis, the cells were centrifuged at 12,000 × g for 5 min at 4 °C, and the supernatant was removed for subsequent assays. One hundred microliters of ATP assay working solution was added to the assay wells. After leaving at room temperature for 5 min, 20 µL of sample or standard was added to the assay wells or tubes, which were mixed quickly with a gun (micropipette), and after an interval of at least 2 s, the RLU value or CPM was determined with a chemiluminescence meter (luminometer) or liquid flash meter.

### Western blot

The cells (1 × 10^6^ cells/well) were cultured in 6–well plates with DDP (3 µg/mL), bCCM (60 µg/mL), or bCCM (60 µg/mL) + DDP (1.5 µg/mL) for 24 h and harvested. The protein concentration in the total extract was quantified via a BCA protein assay kit (562 nm). The expression of GST was detected strictly by western blot (WB) with different antibodies: β–actin (1:2000), xCT polyclonal (1:1000), FDX1 polyclonal (1:1000), DLAT polyclonal (1:2000), ATP7B polyclonal (1:1000), LIAS polyclonal (1:1000), and P–gp polyclonal (1:500) antibodies. Detection was carried out on a BIO–RAD ChemiDoc XRS chemiluminescence system.

### Mitochondrial damage tracking

Cells were seeded into 12–well plates (2 × 10^5^ cells/well) with DDP (3 µg/mL), bCCM (60 µg/mL), or bCCM (60 µg/mL) + DDP (1.5 µg/mL) for 24 h. Subsequently, the cells were stained with MitoTracker Red CMXRos for 30 min, fixed with 4% PFA for 15 min and stained with DAPI (200 µL) for 15 min, followed by fluorescence imaging.

### JC–1 testing

The cells were seeded into 12–well plates (2 × 10^5^ cells/well) with DDP (3 µg/mL), bCCM (60 µg/mL), or bCCM (60 µg/mL) + DDP (1.5 µg/mL) for 24 h. Subsequently, the cells were stained with JC–1 (2.0 µg/mL) staining solution at 37 °C for 20 min, fixed with 4% PFA for 15 min and stained with DAPI (200 µL) for 15 min, followed by fluorescence imaging.

### In vivo imaging

BALB/C mice (5–7 weeks, 18–20 g) were provided by Beijing Vital River Laboratory Animal Technology Co., Ltd., and were randomly divided into two groups (*n* = 3). BEL7402/DDP cells (5 × 10^6^ cells in PBS) were injected subcutaneously into the nude mice. When the tumor volume reached approximately 80 mm^3^, the mice were injected in situ with bCCM@IR783, and small animal imaging was performed. The animal study protocol was approved by the Animal Research Ethics Committee, Hunan University of Chinese Medicine.

### Nude mouse model

Female BALB/c nude rats (5–7 weeks old) were purchased from Beijing Vital River Laboratory Animal Technology Co., Ltd. (China). The animal experimental protocol was approved by the Animal Research Ethics Committee of Hunan University of Traditional Chinese Medicine. BEL7402/DDP cells (1 × 10^7^ cells) were suspended in PBS and then injected into the right hind abdomens of the mice. After two weeks of tumor growth, 10 mice were randomly divided into the following four groups (*n* = 5 in each group): the PBS group, DDP group (5 mg/kg), bCCM group (3 mg/kg) and bCCM + DDP (3 mg/kg + 2.5 mg/kg) group. The mice in the DDP group received 5 mg/kg/day Curcumin treatment (tail vein administration) once every two days. The control mice were injected with 0.9% NaCl. The mice were examined for tumor growth every 2 days. The mice died after intrahepatic injection of sodium pentobarbital. Two weeks after the administration of Curcumin, the mice were injected intraperitoneally with sodium pentobarbital (200 mg/kg). The size of each tumor was measured. Tumor tissue was collected for subsequent experiments. All the mice were euthanized at the end of the treatment. Tumors and major organs were harvested and weighed for H&E–staining (Ethics Approval Number: SLBH–202311090018).

### In vivo antitumor activity assay

The tumors were fixed with 4% PFA (24 h) and then sliced into paraffin Sect. (5 μm). H&E staining and TUNEL experiments were performed according to the manufacturer’s instructions. The expression of DLAT, FDX1, XCT and GPx4 in tumor tissues was detected by immunohistochemistry. The paraffin sections of tumor tissues subjected to different treatments were dewaxed. After acid–base repair, ovulin and d–biotin was added for blocking, and endogenous peroxidase was blocked with a 3% hydrogen peroxide solution. Then, the prepared primary antibody solutions were added to the slides and incubated for 2 h in a wet box away from light. The reaction enhancement solution was incubated for 20 min in a light–resistant environment and then incubated for 20 min in a dark environment with the secondary antibodies labeled with HRP corresponding to the primary antibodies. After DAB staining, the nucleus was restained with hematoxylin for 5 min. The sections were scanned and sealed under an inverted microscope. WB analysis was performed to detect protein expression levels in tumor tissues. The tumor tissues were disrupted with a homogenizer, and RIPA lysis buffer was added to extract total protein. Blood samples were collected for biochemical and hematological assays. Moreover, the heart, liver, spleen, lung and kidney were collected for sectioning and H&E staining.

### Toxicity test of zebrafish embryos

The zebrafish experiments were conducted in accordance with the Animal Ethics Committee of Hunan University of Traditional Chinese Medicine. All experiments used wild–type AB strain zebrafish. Zebrafish embryos were treated with bm–Cur or cisplatin at specified concentrations postfertilization (hpf) and analyzed at 24, 48, 72, or 96 hpf.

### Analysis of pearson’s coefficient calculations

Pearson’s coefficient is used to measure the degree of linear correlation between two variables and has a value between − 1 and 1. A value of 1 represents a positive correlation, − 1 represents a negative correlation, and 0 represents no correlation. ImageJ software was used for Pearson coefficient analysis, ImageJ software was used to separate the color channels, pseudo–colors were added to the color channels, the Coloc2 plug–in was opened, and the algorithm was tickged for calculation.

### Statistical analysis

The data were analyzed with GraphPad Prism 8.4.0 software and are presented as the mean ± S.D. values; *n* = 3 independent experiments. *p ≤ 0.05; **p ≤ 0.01; ***p ≤ 0.001; ****p ≤ 0.0001, whereas ns represents no statistically significant difference compared with the vehicle group according to one–way ANOVA.

## Results

### Design, preparation, and characterization of the Cu–nanocarrier bCCM

**Fig. 1:**
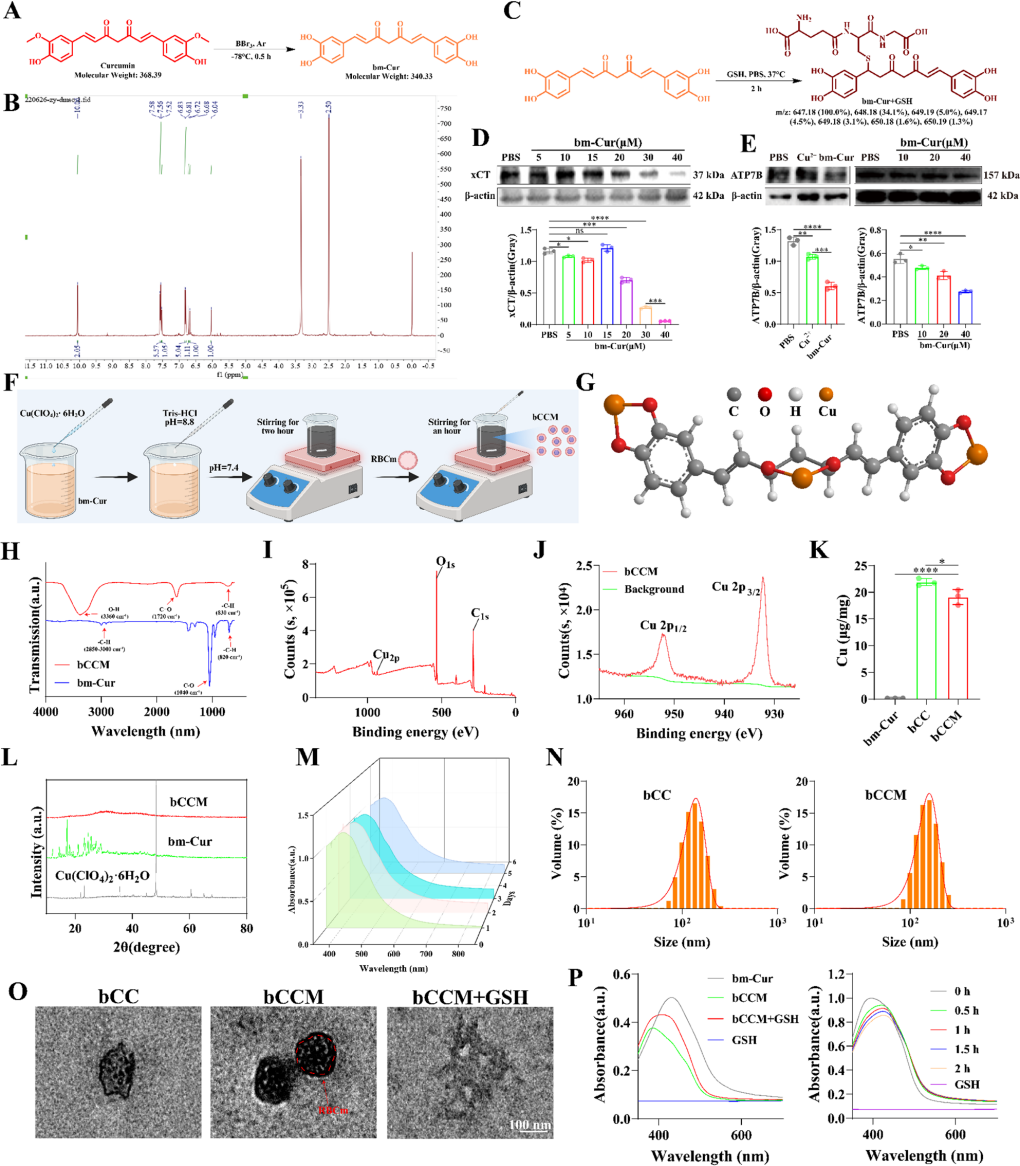
Characterization of the Cu nanocomplex–nanocarrier bCCM and its response to GSH. (**A**) The synthetic process of bm–Cur. (**B**) NMR hydrogen spectra of bm–Cur. 1 H NMR (400 MHz, DMSO–d6) δ 10.06 (s, 2 H), 7.52 (d, J = 16 Hz, 2 H), 6.81 (d, J = 8 Hz, 2 H), 6.72 (dd, J = 36, 16 Hz, 2 H), 6.04 (s, 1 H). (**C**) Reaction process of bm–Cur + GSH. (**D**–**E**) Expression and quantitative analysis of the xCT and ATP7B proteins in BEL7402/DDP cells after different treatments for 24 h. (**F**) Preparation process of bCCM. (**G**) The simulated three–dimensional structure of the bCCM. (**H**) FTIR spectra of bCCM and bm–Cur. (**I**) XPS spectra of bCCM. (J) XPS spectra for Cu 2p of bCCM. (**K**) Determination of Copper Content in bCC (only chelated with copper ions) and bCCM (coated with red blood cell membrane) via ICP–MS. (**L**) XRD pattern of bCCM. (**M**) UV–vis absorption spectral analysis of bCCM over time (Days 1, 2, 3, and 5). (**N**) Size characterization of nanoparticles via DLS. (**O**) TEM images of bCC, bCCM and bCCM + GSH (bCCM reacted with GSH). Scale bar, 100 nm. (**P**) UV–vis spectra of bCCM, bm–Cur, GSH, and bCCM + GSH. UV–vis spectra of bCCM (60 µg/mL) with 10 mM GSH over time. (*n* ≥ 3; error bars represent SD, *p ≤ 0.05; ** *p* ≤ 0.01; *** *p* ≤ 0.001; **** *p* ≤ 0.0001; ns, not significant)

As a constituent unit of the Cu–nanocarrier bCCM, the bm–Cur compound was synthesized via efficient demethylation of curcumin (Fig. 1A). The structural characterization was confirmed by 1 H NMR spectrum analysis and Low resolution liquid chromatography-mass spectrometry (LRLC–MS) (Fig. 1B and Fig. S1), which revealed a curcumin parent nucleus rich in four phenolic hydroxyl groups and two α, β–unsaturated ketones. Considering that bm–Cur with an α, β–unsaturated ketone structure can react with the sulfhydryl group of GSH to form a bm–Cur–SG complex through Michael addition, HR–MS and electrospray ionization (ESI) were performed to confirm the successful reaction between bm–Cur (C₁₉H₁₆O₆) and GSH. As expected, ESI^−^ analysis of the reaction between C₁₉H₁₆O₆ and GSH (Mw = 647.18) revealed a molecular ion peak at m/z = 646.1423 (Fig. 1C and Fig. S2). Furthermore, the UV–vis spectra revealed an absorbance peak at 430 nm in the sample containing bm–Cur alone. However, the absorbance intensity of bm–Cur at 430 nm decreased in a concentration–dependent manner with increasing GSH concentration (0–10 mM). Notably, the absorbance of bm–Cur progressively decreased over time at 10 mM GSH (Fig. S3), further confirming its responsiveness to GSH. Moreover, bm–Cur inhibited GSH synthesis by downregulating xCT expression (Fig. 1D) and reducing the intracellular GSH level in a concentration–dependent manner (Fig. S4). Further tests on the cytotoxicity of bm–Cur toward tumor cells revealed a certain ability to kill both cisplatin–sensitive (HepG2 and Hepa1–6) and resistant tumor cells (BEL7402/DDP and HepG2/DDP) (Fig. S5 and S6). Moreover, bm–Cur significantly and concentration–dependently downregulated the expression of ATP7B, a copper ion and a cisplatin efflux protein (Fig. 1E).

Inspired by the remarkable properties of bm–Cur, which leverages the strong metal–chelating ability of its adjacent phenolic hydroxyl groups and ortho–diketone moieties, the supramolecular nanocomplex bCCM was prepared by directly chelating bm–Cur with Cu (II) in Tris HCl buffer supplemented with encapsulation in red blood cell membranes (RBCm) (Fig. 1F). The simulated three–dimensional structure of bCCM clearly shows the three coordination sites of Cu (II) (Fig. 1G). Infrared spectroscopy (IR) analysis revealed characteristic absorption peaks corresponding to hydroxyl groups at 3300–3600 cm^–^¹ and carbonyl and C–O stretching vibrations at 1750–1700 cm^–^¹ and 1300–1000 cm^–^¹, respectively (Fig. 1H). X–ray photoelectron spectroscopy (XPS) analysis revealed the presence of C, O, and Cu, with Cu (II) coordination peaks at 934 eV (Cu 2p_3/2_) and 954 eV (Cu 2p_1/2_) observed (Fig. 1I and J). Additionally, ICP–MS analysis revealed that the copper content in bCC was approximately 21.7 µg/mg, significantly higher than that in bm–Cur (0.133 µg/mg). In contrast, the copper content per mg of bCCM was slightly lower than that of bCC, which can be attributed to the increased molecular weight of the nanoparticles resulting from erythrocyte membrane (RBCm) coating (Fig. 1K). These results confirmed the formation of bCCM containing Cu (II). X–ray diffraction (XRD) analysis revealed that bCCM has almost no diffraction peaks, indicating that the bCCM has an amorphous structure with low crystallinity (Fig. 1L). The ultraviolet absorption spectra of bCCM were measured at days 1, 2, 3, and 5 to evaluate its stability (Fig. 1M). The results showed no significant changes in the UV absorption peaks of bCCM over this period. The stability half–life of bCCM was determined to be 149.3 h in PBS and 106 h in serum, indicating that bCCM exhibits excellent stability at room temperature (Fig. S7). Furthermore, the nanocomplexes exhibited excellent solubility and stability in various solutions, including normal saline (0.9% NaCl), PBS, and DMEM (Fig. S8), highlighting their suitability for in vitro and in vivo applications. Subsequently, the particle sizes of bCC and bCCM nanoparticles were characterized using dynamic light scattering (DLS). The results showed that the average particle size of bCC was 136.29 nm, while that of bCCM was 154.64 nm. This increase in particle size confirms the successful coating of bCC nanoparticles with the RBCm (Fig. 1N). TEM images revealed that the uniform sheet structure of nanocomplex bCC formed via the chelation of bm–Cur with copper ions. After coating with RBCm, a sphere–like structure of bCCM formed (Fig. 1O). The rapid disassembly of bCCM through a Fenton reaction between Cu (II) and GSH could deplete GSH into GSSG[45] and release single bm–Cur. The morphology of bCCM subsequently changed from flake to dispersed fragments after reacting with GSH, indicating that bCCM had been cracked by GSH, which further confirmed the response of bCCM to GSH (Fig. 1O). We then investigated the GSH consumption capability of bCCM via UV–vis spectral analysis. UV–vis spectra revealed an absorbance peak at 430 nm in the bCCM + GSH group but not in the bCCM or GSH groups (Fig. 1P). Moreover, the absorbance intensity decreased gradually with increasing GSH concentration (0–10 mM) in the bCCM + GSH group (Fig. S9) and decreased over time at 10 mM GSH (Fig. 1P). These results confirmed the response capability of bCCM to GSH and the release of bm–Cur from bCCM. These findings demonstrate that bCCM is responsive to GSH and subsequently releases bm–Cur, confirming its GSH–triggered degradation behavior. Collectively, these results demonstrate the efficient GSH consumption capability of bCCM. Moreover, the concentration of bm–Cur released from bCCM (90 µg/mL) upon GSH treatment was calculated to be 39.22 µM on the basis of changes in the absorbance at 430 nm (Fig. S10).

### In vitro anti–resistant tumor efficacy of bCCM

**Fig. 2:**
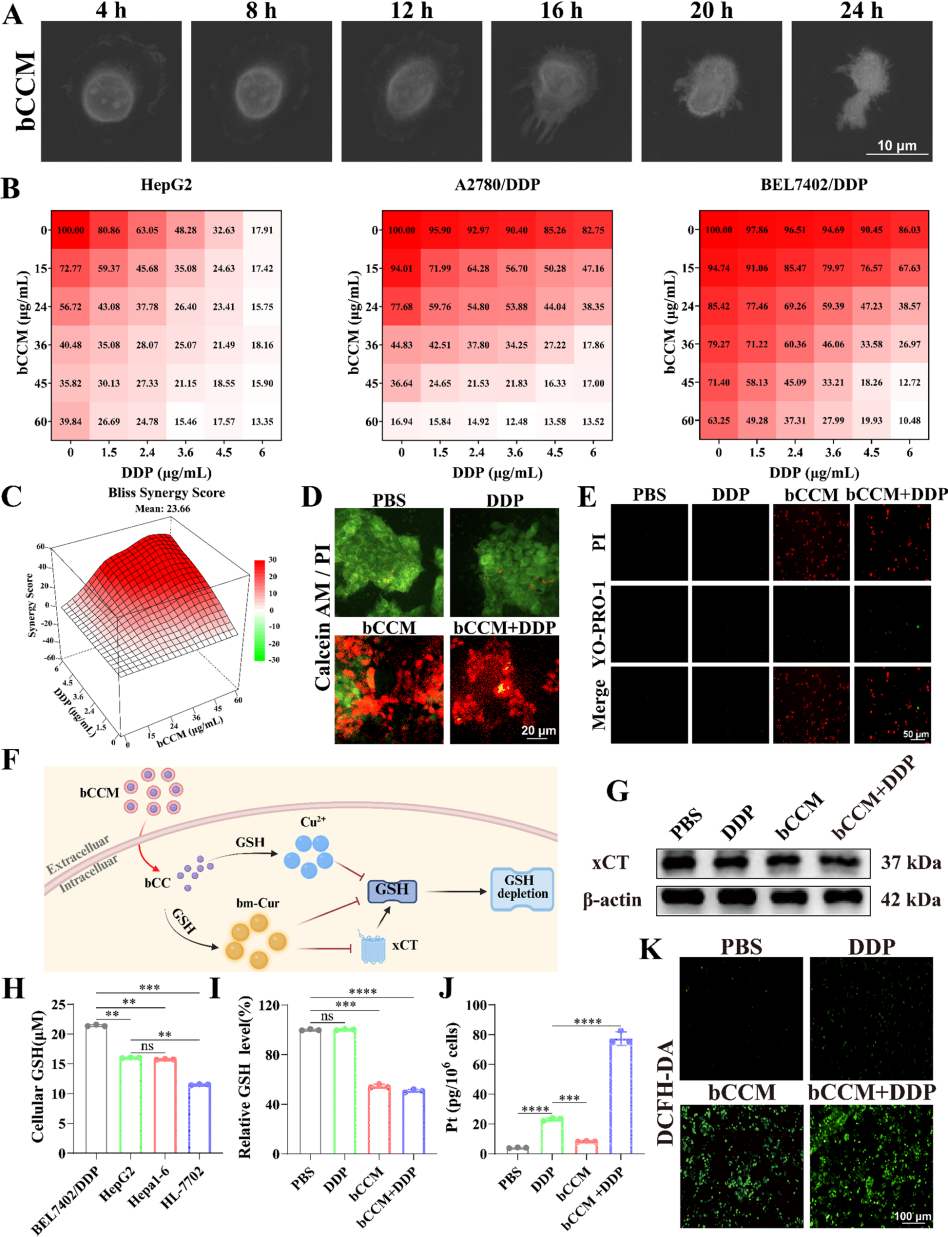
In vitro antitumor effects of bCCM and its synergistic effect with DDP, as well as the investigation of their ability to induce GSH depletion and ROS generation. (**A**) Monitoring of single BEL7402/DDP cell morphological changes after bCCM treatment at different time points via holographic microscopy. Scale bar, 10 μm. (**B**) Cytotoxicity of bCCM combined with DDP in HepG2, BEL7402/DDP and A2780/DDP cells for 24 h. (**C**) The quantitative analysis of the synergistic interaction between bCCM and DDP. (**D**) CLSM images of live/dead staining of BEL7402/DDP cells after different treatments for 24 h. Scale bar, 100 μm. (**E**) The necrosis and apoptosis imaging of BEL7402/DDP cells after different treatments for 24 h. Scale bar, 50 μm. (**F**) Proposed activation mechanism of bCCM depleting GSH. (**G**) Western blot of xCT protein in BEL7402/DDP cells treated for 24 h. (**H**) GSH level detection in BEL7402/DDP, HepG2, Hepa1–6 and HL7702 cells. (**I**) GSH level detection in BEL7402/DDP cells subjected to different treatments for 24 h. (**J**) The intracellular Pt content in BEL7402/DDP cells after different treatments was analyzed by ICP–MS. (**K**) The ROS levels in BEL7402/DDP after different treatments for 24 h. Scale bar, 100 μm. [DDP] = 3 µg/mL, [bCCM] = 60 µg/mL, [bCCM + DDP] = 60 µg/mL + 1.5 µg/mL. (*n* ≥ 3; error bars represent SD, *p ≤ 0.05; ** *p* ≤ 0.01; *** *p* ≤ 0.001; **** *p* ≤ 0.0001; ns, not significant)

First, the effects of bCC and bCCM on cell viability were assessed in BEL7402/DDP. Both showed comparable inhibitory activity at equimolar concentrations and already exhibited a substantial reduction in cell viability (60%) at 60 µg/mL (Fig. S11). This result indicates that the nanoparticles have little effect on the cell viability before and after being encapsulated in RBCm. Next, morphological alterations were observed in single cisplatin-resistant tumor cells (BEL7402/DDP) following treatment with bCCM. As shown in Fig. 2A, exposure to 60 µg/mL bCCM over 0–24 h induced progressive cell swelling and membrane rupture, culminating in critical lysis and cytoplasmic dispersion. Next, bCCM and DDP monotherapy groups was used as control, systematically compared the effects of the bCCM + DDP combination group to further investigate their synergistic antitumor action. Combination of bCCM with DDP had a significant additive antitumor effect on both DDP–resistant cell lines. In the BEL7402/DDP cells, the combined treatment (60 µg/mL bCCM + 1.5 µg/mL DDP) reduced the cell viability to 49.28%, whereas the viability was 63.25% with bCCM alone and 97.86% with DDP alone. These results clearly indicate that bCCM can effectively sensitize DDP–resistant tumor cells to cisplatin treatment (Fig. 2B). Subsequently, quantitative analysis of synergistic effects was performed using the Bliss model. The results indicated that the synergistic effects were not significant in HepG2 and A2780/DDP cells, which may be attributed to the potent efficacy of the individual drugs. In contrast, the Bliss score for BEL7402/DDP cells was 23.66, substantially greater than 0, demonstrating strong synergistic activity in this cell line. To reduce the concentration of DDP, the drug combination with the strongest synergistic effect and the lowest DDP concentration was selected: [bCCM + DDP] = 60 µg/mL + 1.5 µg/mL (Fig. 2C) and based on preliminary studies, the concentration of cisplatin in the positive control group was set at 10 µM[47]. The cytotoxicity of bCCM was subsequently assessed using the MTT assay, with cisplatin–resistant (BEL7402/DDP, A2780/DDP) and cisplatin–sensitive (HepG2) tumor cells serving as controls. As illustrated in Fig. S12 and S13, compared with the relatively weak cytotoxicity of cisplatin (IC_50_ values of (17.8 ± 0.4) µg/mL in BEL7402/DDP cells and (19.9 ± 0.5) µg/mL in A2780/DDP cells), bCCM exhibited stronger cytotoxicity against cisplatin–resistant cells. After 24 h of treatment, the half–maximal inhibitory concentration (IC_50_ of bCCM was calculated to be 80.9 ± 1.2 µg/mL) in BEL7402/DDP cells and (40.2 ± 0.6) µg/mL in A2780/DDP cells (Table. S1). Furthermore, bCCM demonstrated excellent biosafety in murine normal hepatocytes (AML–12) and human normal hepatic stellate cells (LX–2), maintaining cell viability above 95% even at concentrations (90 µg/mL) significantly higher than therapeutic levels for cisplatin–resistant cells (BEL7402/DDP and A2780/DDP) (Fig. S14 and S15). In contrast, cisplatin at 6 µg/mL reduced LX–2 cell viability to below 60%, indicating pronounced cytotoxicity. These results suggest that in combination therapy with bCCM and cisplatin, the cisplatin concentration should be minimized as much as possible, highlighting the selective cytotoxicity of bCCM toward tumor cells.

A subsequent live/dead cell costaining assay further confirmed the high cytotoxicity of bCCM in BEL7402/DDP cells. Confocal laser scanning microscopy (CLSM) images revealed abundant red fluorescence of dead cells in the bCCM– and bCCM + DDP–treated groups but abundant green fluorescence of living cells in the control and DDP groups, indicating strong cytotoxicity of bCCM and bCCM + DDP in BEL7402/DDP cells (Fig. 2D and Fig. S16). The results of necrosis and apoptosis imaging further verified the reliability of the above findings. Compared with the control, bCCM + DDP group showed a more obvious green fluorescence signal, indicating that bCCM can enhance the ability of DDP to induce apoptosis. In addition, the bCCM and bCCM + DDP groups exhibited stronger red fluorescence, while the red fluorescence of the DDP group alone could be ignored (Fig. 2E and Fig. S17). These results clearly demonstrate the excellent efficacy of bCCM and its DDP sensitization effect on cisplatin–resistant BEL7402/DDP cells.

The successful determination of the extracellular GSH reaction activity of the bCCM nanocomplex led us to further explore its ability to consume intracellular GSH and generate ROS. The effects of various treatments on the cytoplasmic expression of xCT were examined. The results revealed significant downregulation of xCT in bCCM– and bCCM + DDP–treated BEL7402/DDP cells, which effectively inhibited GSH synthesis (Fig. 2F and G and Fig. S18). Next, the GSH content was measured in four liver–related cell lines: cisplatin–resistant human HCC cells (BEL7402/DDP), drug–sensitive human and mouse HCC cells (HepG2 and Hepa 1–6), and normal liver cells (HL–7702). As shown in Fig. 2H, the GSH concentration in drug–resistant BEL7402/DDP cells was (21.42 ± 0.15) µM, which was much greater than that in normal liver cells (11.51 ± 0.10) µM and moderately greater than that in drug–sensitive HepG2 cells (16.04 ± 0.06) µM and Hepa 1–6 cells (15.76 ± 0.09) µM. Next, the test of the GSH consumption capacity at different concentrations of bCCM revealed that bCCM consumed GSH in a concentration–dependent manner in both BEL7402/DDP and HepG2 cells (Fig. S19). Additionally, GSH levels in BEL7402/DDP and HepG2 cells subjected to different treatments were detected, and neither bCCM nor bCCM + DDP depleted GSH significantly differently. In contrast, DDP had almost no ability to consume GSH in either BEL7402/DDP (Fig. 2I) or HepG2 cells (Fig. S20). As shown in Fig. 2J, the intracellular accumulation of cisplatin in BEL7402/DDP cells after 24 h of different treatments was measured using ICP–MS. The platinum accumulation in the bCCM + DDP group was 3.5 times higher than that in the DDP–alone group, demonstrating that bCCM not only reduces intracellular GSH levels but may also inhibit the efflux of DDP. Moreover, the overall level of intracellular ROS was evaluated via confocal laser scanning microscopy (CLSM) imaging with 2′,7′–dichlorofluorescein diacetate (DCFHDA) as an indicator, which is rapidly oxidized by ROS to generate the green fluorescence dichlorofluorescein (DCF) [48]. Notably, a sharp increase in DCF fluorescence was observed in the bCCM and bCCM + DDP groups after 6 h of incubation compared with the control (Fig. 2K). In contrast, there was only a small increase in the fluorescence intensity of the DDP group, indicating that bCCM is a strong ROS inducer. These results demonstrated that bCCM could directly deplete intracellular GSH, increasing ROS levels.

### bCCM–induced cuproptosis and RNA sequencing analysis

**Fig. 3:**
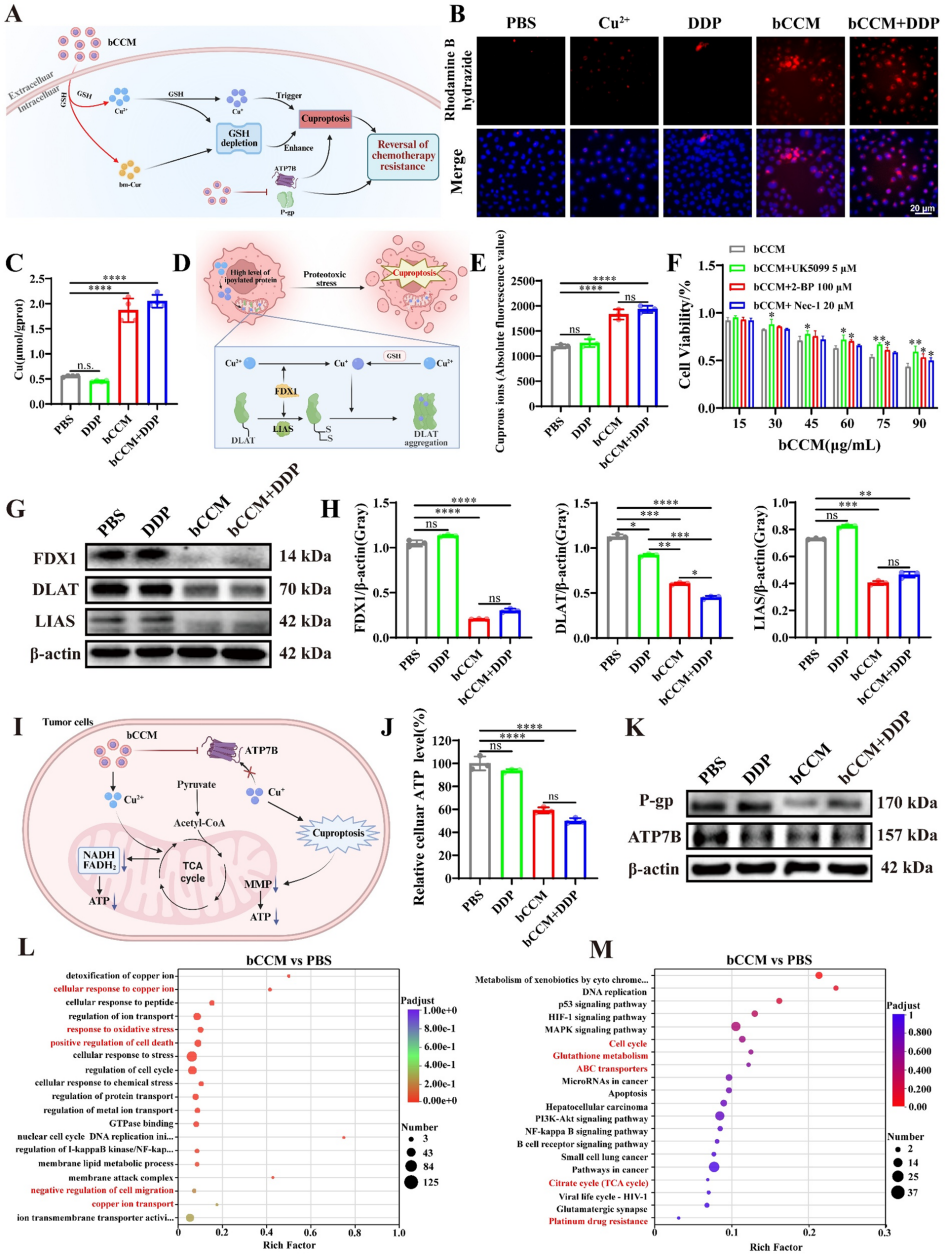
Investigation of cuproptosis–related characteristics and drug resistance–related protein expression and transcriptomic analysis of BEL7402/DDP cells after 24 h of various treatments. (**A**) Proposed mechanism by which bCCM induces cuproptosis to reverse resistance. (**B**, **C**) Imaging (Scale bar, 20 μm.) and quantitative detection of intracellular copper ion levels were performed after different treatments for 24 h. (Cu^2+^: 15 µg/mL, DDP: 3 µg/mL, bCCM: 60 µg/mL, bCCM + DDP: 60 µg/mL + 1.5 µg/mL). (**D**) The mechanism for the reduction of Cu²⁺ to cuprous Cu⁺ ions. (**E**) Intracellular Cu^+^ level detection after different treatments. (**F**) The viability of bCCM combined with the cuproptosis inhibitor UK5099 (5 µM), pyroptosis inhibitor 2–BP (100 µM) and necroptosis inhibitor Nec–1 (20 µM). (**G**, **H**) Western blot analysis of the expression of cuproptosis–related proteins (FDX1, DLAT and LIAS) in BEL7402/DDP cells. (**I**) Mechanistic diagram of the relationship between ATP and cuproptosis. (**J**) Intracellular ATP level detection after different treatments. (**K**) Western blot analysis of cisplatin resistance–related protein (P–gp, ATP7B) expression in BEL7402/DDP cells. (**L**) Gene Ontology (GO) enrichment analysis. (**M**) Kyoto Encyclopedia of Genes and Genomes (KEGG) pathway analysis. [DDP] = 3 µg/mL, [bCCM] = 60 µg/mL, [bCCM + DDP] = 60 µg/mL + 1.5 µg/mL. (*n* ≥ 3; error bars represent SD, * *p* ≤ 0.05; ** *p* ≤ 0.01; *** *p* ≤ 0.001; **** *p* ≤ 0.0001; ns, not significant)

As mentioned above, GSH consumption can induce cuproptosis by decreasing chelation with copper and increasing intracellular copper ion levels, inhibiting the activity of FDX1 and DLAT and leading to enhanced therapeutic efficacy in drug–resistant tumors (Fig. 3A). Because of the superior GSH consumption ability and copper ion–carrying ability of bCCM, we hypothesized that bCCM could significantly induce cuproptosis. The intracellular copper ion levels were subsequently monitored by a copper detection probe after different treatments. As we expected, compared with those in the control or DDP–treated groups, copper ion levels dramatically increased in bCCM– and bCCM + DDP–treated BEL7402/DDP cells, as indicated by the strong red fluorescence (Fig. 3B and Fig. S21). Moreover, the results of the quantitative analysis of intracellular copper ions confirmed the above findings (Fig. 3C). These results indicated that copper ions were loaded into the cells via bCCM. Moreover, Cu (II) showed no significant cytotoxicity to BEL7402/DDP cells within a certain concentration range (Fig. S22). Furthermore, considering that copper ions are further converted into Cuprous ions in the cytoplasm through GSH or FDX1 (Fig. 3D), the intracellular Cuprous ion levels were detected. Compared with DDP alone, both bCCM and bCCM + DDP significantly increased intracellular cuprous ion levels (Fig. 3E), which suggests that bCCM can carry copper ions into the cell and convert them to cuprous ions to trigger cuproptosis.

Encouraged by the observed intracellular copper influx and cuprous ion conversion, we investigated whether bCCM induces cuproptosis by assessing cell viability in combination with specific inhibitors. Compared to bCCM alone, co-treatment with the cuproptosis inhibitor UK5099 (5 µM) significantly increased viability by 15–17%, while UK5099 alone showed no effect (Fig. S23). In contrast, the pyroptosis inhibitor 2‑BP (100 µM)[49] and the necroptosis inhibitor Nec‑1 (20 µM) [50] led to much smaller increases in viability (Fig. S23). These results suggest bCCM acts primarily through the cuproptosis pathway (Fig. 3F). To further confirm the role of copper, cell viability was tested after combining bCCM with the copper chelator TTM. Co-treatment with 50 nM TTM increased viability by approximately 20%, supporting the key role of copper ions in mediating cell death (Fig. S24). Western blot analysis revealed that bCCM significantly reduced protein levels of FDX1, DLAT, and LIAS—three characteristic cuproptosis suppressors—confirming effective cuproptosis induction in BEL7402/DDP cells (Fig. 3G and H).

ATP7B is a key copper transporter responsible for Cu⁺ and cisplatin efflux, playing a crucial role in regulating intracellular copper levels [26]. Under elevated extracellular copper, ATP7B translocates to the cytoplasm, binds copper, and promotes ATP hydrolysis at its ATP-binding domain, releasing energy for transport [51, 52] Subsequently, it relocates to the cell membrane to export copper ions[53]. Given that ATP hydrolysis energizes ATP7B-mediated efflux, we measured intracellular ATP levels following treatment. Both the bCCM and bCCM + DDP groups showed a sharp decrease in ATP (Fig. 3I and J), indicating impaired ATP7B activity. Consistent with this, Western blot analysis confirmed significant downregulation of ATP7B and P-gp in these groups (Fig. 3K and S25). The downregulation of ATP7B expression promotes cuproptosis and helps overcome cisplatin resistance. Additional Western blot analyses indicated that bCCM may also activate necroptosis and pyroptosis pathways, with pyroptosis-related changes being particularly notable (Fig. S26–S28). Taken together, these data suggest that bCCM induces cuproptosis partly by suppressing ATP7B expression and depleting intracellular ATP, thereby inhibiting copper efflux. The concurrent downregulation of P-gp may further contribute to reversing drug resistance by modulating membrane permeability and function.

To verify the above results, genome–wide high–throughput RNA sequencing (RNA–Seq) was performed on BEL7402/DDP cells treated with bCCM and PBS as a control. Gene Ontology (GO) term analysis provided insights into the enrichment of DEGs related to the cellular response to copper ions, oxidative stress, regulation of cell death, cell migration, and copper ion transport (Fig. 3L). Notably, “copper ions” associated with Cuproptosis, which is a key inducer of Cuproptosis, were the most significantly enriched terms. Kyoto Encyclopedia of Genes and Genomes (KEGG) analysis further classified and annotated these DEGs, revealing that the upregulated DEGs induced by bCCM are closely correlated with the cell cycle, glutathione metabolism, ABC transporters, the citrate cycle (TCA cycle), hepatocellular carcinoma, and platinum drug resistance (Fig. 3M). Furthermore, gene set enrichment analysis (GSEA) revealed positively and negatively regulated pathways after bCCM administration (Fig. S29A). Notably, pathways related to “glutathione metabolism”, “citrate cycle (TCA cycle)”, and “ABC transporters” were upregulated, whereas “platinum drug resistance”–associated pathways were downregulated. These findings strongly suggest that the therapeutic mechanism of bCCM against resistant HCC involves the induction of cuproptosis and the modulation of glutathione metabolic and mitochondrial pathways. Finally, the protein–protein interaction (PPI) network of 15 proteins related to platinum resistance (ABCB1, GSTA1) revealed close interactions between proteins involved in these processes (Fig. S29B). These transcriptomics analysis results confirm the role of bCCM in inducing cuproptosis and further demonstrate the close relationships among cuproptosis, platinum drug resistance, and hepatocellular carcinoma (HCC) in cisplatin–resistant HCC cells (BEL7402/DDP).

### bCCM induces cuproptosis–mediated mitochondrial dysfunction

**Fig. 4:**
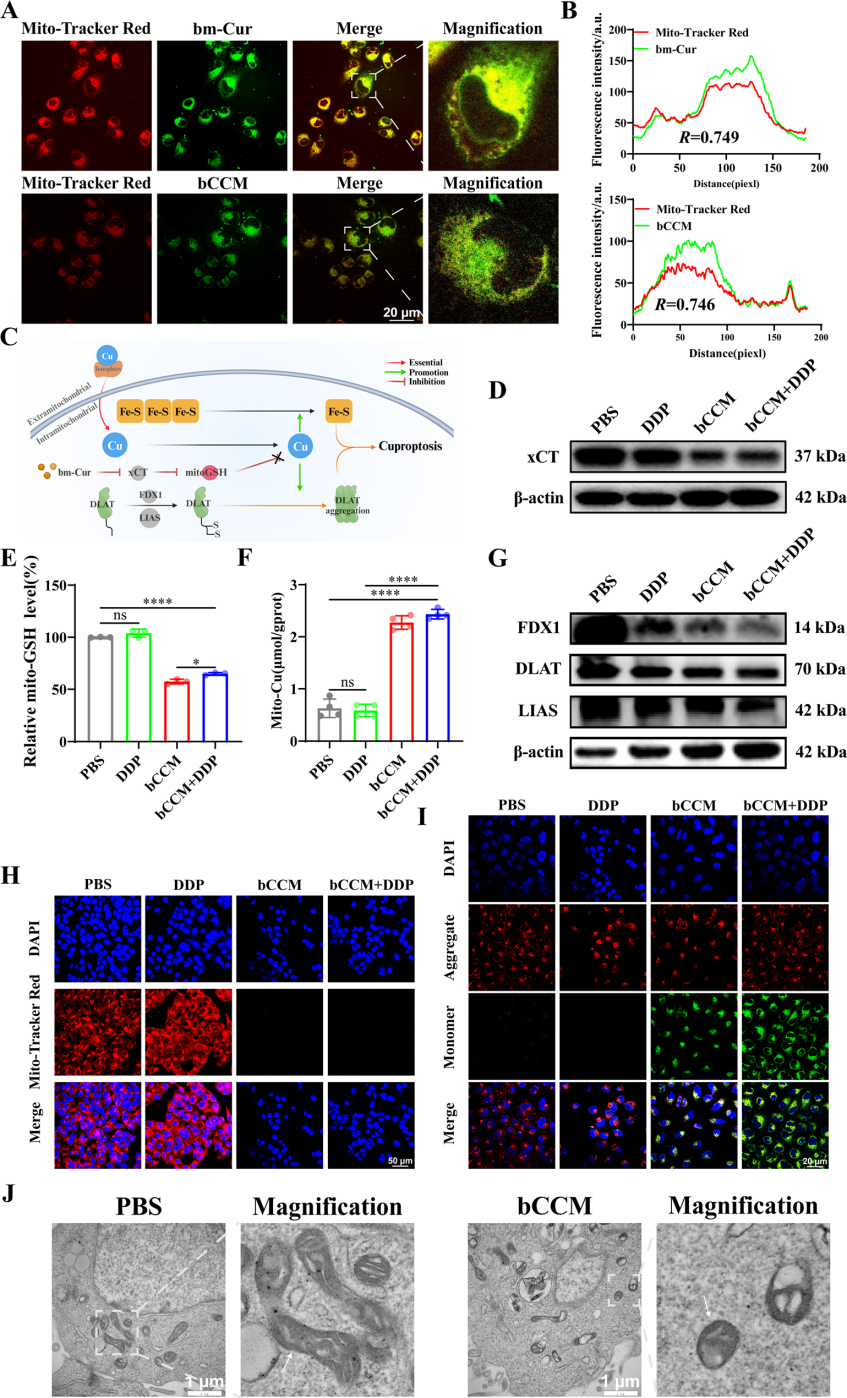
bCCM induces mitochondrial cuproptosis and mitochondrial damage. (**A**–**B**) Colocalization of bCCM and bm–Cur with the MitoTracker Red probe after 0.5 h of coincubation in BEL7402/DDP cells. Scale bar, 20 μm. (**C**) The mechanism of cuproptosis induced by bCCM in mitochondria. (**D**) Mito–protein expression of xCT in BEL7402/DDP cells after various treatments. (**E**) Mitochondrial GSH (Mito–GSH) levels are depicted in BEL7402/DDP cells. (**F**) Copper ion content in BEL7402/DDP cell mitochondria after various treatments. (**G**) Mitochondrial FDX1, DLAT, and LIAS expression in BEL7402/DDP cells subjected to different treatments. (**H**) CLSM images of mitochondrial membrane damage in BEL7402/DDP cells after 24 h of treatment by MitoTracker Red CMXRos^579nm/599nm^ monitoring. Scale bar, 20 μm. (**I**) JC–1 detection in BEL7402/DDP cells after different treatments for 24 h. Scale bar, 20 μm. (**J**) Bio–TEM images of BEL7402/DDP cells treated with PBS and bCCM. [bm–Cur] = 40 µM, [DDP] = 3 µg/mL, [bCCM] = 60 µg/mL, [bCCM + DDP] = 60 µg/mL + 1.5 µg/mL. (*n* ≥ 3; error bars represent SD, *p ≤ 0.05; ** *p* ≤ 0.01; *** *p* ≤ 0.001; **** *p* ≤ 0.0001; ns, not significant)

As pivotal players in cuproptosis and targets for cancer therapy, targeting mitochondria has tremendous therapeutic potential for drug–resistant cancer[54]. Cuproptosis cells morphologically exhibit features of mitochondrial dysfunction, including disruption of the mitochondrial structure and a reduction in the mitochondrial membrane potential [55]. Increasing damage to mitochondrial membranes and inducing dysfunction are classic characteristics of cuproptosis. To confirm that bCCM induces cuproptosis in mitochondria, the mitochondrial targeting ability of bCCM was first evaluated. Confocal microscopy analysis revealed clear and rapid colocalization (0.5 h) of bCCM and bm–Cur with mitochondria in BEL7402/DDP cells (Fig. 4A and B). The ability of bCCM to induce mitochondrial dysfunction was subsequently explored. A mitochondrial REDOX imbalance, which is induced by mitochondrial GSH consumption and copper ion imbalance in response to cuproptosis, is a significant manifestation of mitochondrial dysfunction (Fig. 4C). The mitochondrial GSH and copper ion levels in BEL7402/DDP cells were studied. First, the downregulation of xCT in mitochondria by bCCM suggested that it can also deplete mitochondrial GSH by inhibiting its synthesis (Fig. 4D). As shown in Fig. 4E and Fig. S30, the lowest GSH levels were detected in the mitochondria of BEL7402/DDP cells treated with bCCM compared with those treated with cisplatin alone, resulting in a 42.8% (bCCM) to − 3.9% (DDP) decrease in GSH levels, indicating that the greatest increase in mitochondrial GSH consumption was caused by bCCM. Notably, the decrease in GSH levels in the bCCM + DDP group was similar to that in the bCCM group and much greater than that in the DDP group (35.1% vs. − 3.9%). Moreover, compared with the DDP group, both the bCCM and bCCM + DDP groups accumulated more mitochondrial copper (3.2 ~ 3.4–fold) (Fig. 4F). Further investigation of the expression of classic mitochondrial cuproptosis proteins (DLAT, FDX1 and LIAS) revealed that bCCM– and bCCM + DDP–treated BEL7402/DDP cells presented significantly lower protein levels than DDP–treated cells did (Fig. 4G and Fig. S31). Together, these findings confirmed that bCCM could indeed trigger cuproptosis in mitochondria.

Given that mitochondrial membrane potential (Δψm) integrity and variation are critical for mitochondrial function, we investigated the Δψm using Mito–Tracker Red CMXRos^579nm/599nm^, a mitochondrial membrane potential indicator[56]. Significant red fluorescence was observed in the BEL7402/DDP cells in the control and DDP groups (indicating a normal Δψm) after 24 h of incubation, demonstrating that DDP alone had negligible effects on the Δψm. In contrast, red fluorescence was markedly diminished in cells treated with bCCM or bCCM + DDP (Fig. 4H), reflecting severe mitochondrial membrane damage induced by bCCM. Additionally, JC–1 was used to confirm the dissipation of the mitochondrial membrane potential, which remains as a monomer in damaged mitochondria with a dissipated Δψm and aggregates in normal mitochondria with a high Δψm [57]. For BEL7402/DDP cells, in the control and DDP groups, JC–1 aggregates constituted the majority. In contrast, the number of JC–1 aggregates significantly decreased after bCCM and bCCM + DDP treatments, implying the most severe decrease in the Δψm (Fig. 4I), which is consistent with the Mito–Tracker Red results. Next, morphological changes in mitochondria were directly observed via bio–TEM to evaluate mitochondrial damage in BEL7402/DDP cells. Compared with the normal morphology of the mitochondria in the PBS–treated group, the mitochondria in the bCCM–treated group presented severe destruction and swelling, increased membrane density, decreased volume, and reduced cristae (Fig. 4J), characteristic of mitochondrial dysfunction caused by cuproptosis. Taken together, bCCM effectively targets mitochondria and induces mitochondrial cuproptosis–like dysfunction, which is characterized by changes in the expression of cuproptosis–related proteins, mitochondrial morphological damage and membrane potential dissipation. These mitochondrial effects, combined with cytoplasmic cuproptosis, synergistically enhance cuproptosis and improve therapeutic efficacy against DDP–resistant tumors.

### Biosafety evaluation of bCCM in zebrafish and mouse models

**Fig. 5:**
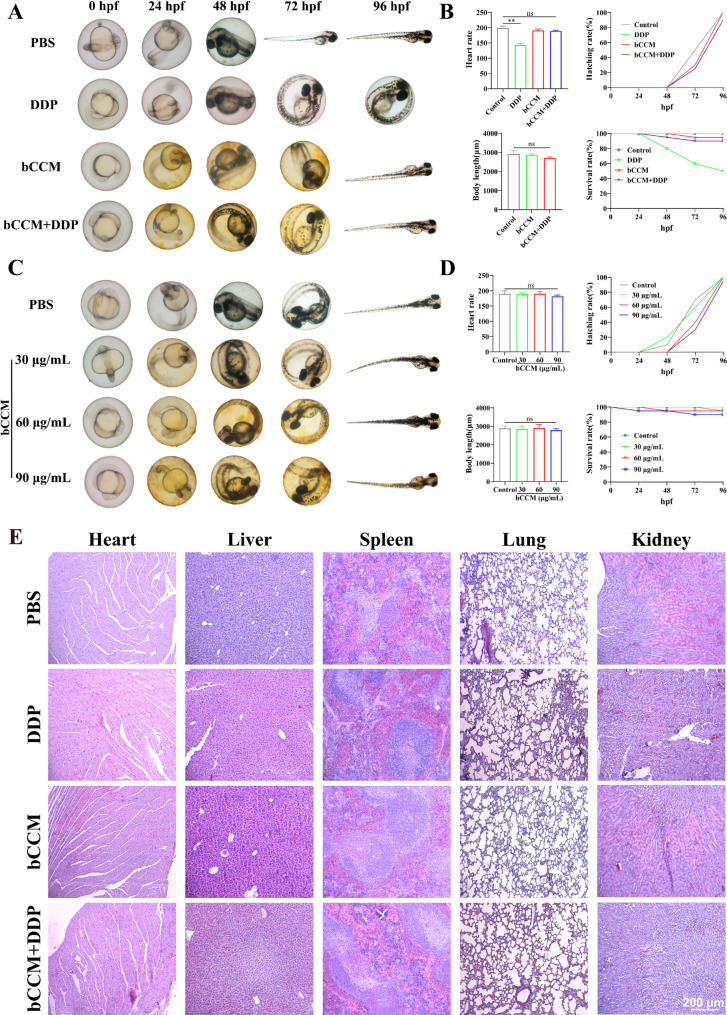
Biosafety assay of bCCM. (**A**) Representative images of zebrafish embryos subjected to different treatments at various time points. (**B**) Heart rate, hatching rate, body length and survival statistics of zebrafish under different treatments. (**C**) Representative images of zebrafish embryos at various time points after treatment with different concentrations of bCCM. (**D**) Heart rate, hatching rate, body length and survival statistics of zebrafish after treatment with different concentrations of bCCM. (**E**) H&E staining of the main organs (heart, liver, spleen, lung, and kidney) of the mice in the different treatment groups. Scale bars, 200 μm. [DDP] = 3 µg/mL, [bCCM] = 60 µg/mL, [bCCM + DDP] = 60 µg/mL + 1.5 µg/mL. (*n* ≥ 3; error bars represent SD, *p ≤ 0.05; ** *p* ≤ 0.01; *** *p* ≤ 0.001; **** *p* ≤ 0.0001; ns, not significant)

After confirming the in vitro cuproptosis effect of bCCM, the biosafety of bCCM was evaluated in zebrafish and mouse models. Considering that zebrafish are highly useful bioassays in toxicological studies, the in vivo toxicity of bCCM was first evaluated in a zebrafish model. Zebrafish embryos were equally divided into 8 groups and coincubated with cisplatin, bCCM, bCCM + DDP, or different concentrations of bCCM. Representative images and survival curves of zebrafish embryos were acquired at different time points (Fig. 5A and B and Fig. S32). At 48 hpf, 72 hpf, and 96 hpf, the cisplatin–treated zebrafish embryos appeared dead. At 96 hpf, representative images of zebrafish larvae were captured, which revealed that both the bCCM and bCCM + DDP groups developed normally. In contrast, DDP treatment resulted in the failure of zebrafish embryos to hatch. The heart rate of zebrafish larvae at 96 hpf was measured in all groups to assess the physiological effects. No significant changes were observed in the bCCM group compared with the control and combined groups, whereas the heart rate was reduced in the DDP group. In addition, no significant changes in body length were observed in the bCCM group compared with the control and combined groups (Fig. 5C). Moreover, there were no significant changes in the hatching rate, survival rate, body length or heart rate of zebrafish larvae in the bCCM group with different concentrations of bCCM compared with those in the control group (Fig. 5D), indicating that bCCM has excellent biocompatibility.

To further explore the antitumor effect and mechanism of therapy in vivo, the biosafety of bCCM in mice was evaluated. First, the blood physiological and biochemical parameters of the mice treated with different drugs were investigated. Prior to in vivo treatments, red blood cell (RBC) hemolysis and coagulation assays were conducted, revealing no hemolysis or coagulation phenomena within the tested concentrations (Fig. S33), indicating good blood compatibility of bCCM. Next, four groups, namely, PBS, DDP, bCCM, and bCCM + DDP, were injected into healthy mice through the tail vein four consecutive times. Thereafter, the blood, major tissues, and organs of the mice treated with different drugs were collected for further analysis on day 14. The results revealed that blood biochemical indices and parameters, including WBC, RBC, HGB, ALT, AST, PLT, CREA, and BUN, were not significantly different from those in PBS–treated mice (Fig. S34), demonstrating negligible side effects of bCCM. Additionally, H&E staining of major organs (heart, liver, spleen, lung, and kidney) revealed no morphological abnormalities (Fig. 5E). In contrast to DDP, which significantly caused liver and spleen damage, bCCM did not affect animal growth or damage any major organs, indicating the superior biocompatibility of bCCM.

### Biodistribution of bCCM and copper ions in a DDP–resistant HCC model

**Fig. 6:**
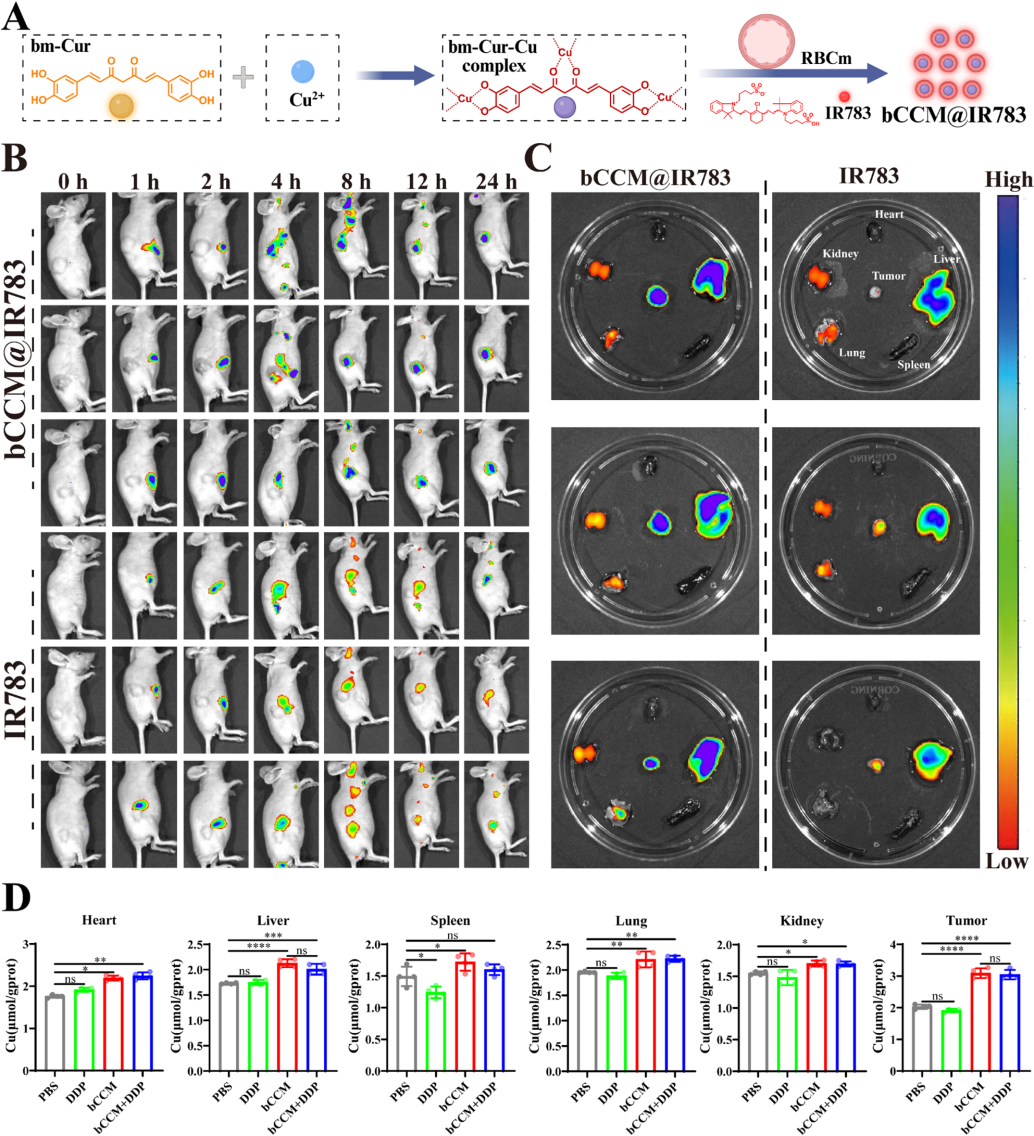
Biosafety and Biodistribution of bCCM. (**A**) Diagram of the preparation process of bCCM@IR783. (**B**) Fluorescence images of IR783 and bCCM@IR783 signals in tumors. (**C**) Fluorescence signals of IR783 and bCCM@IR783 in major organs and tumors. (**D**) Determination of the copper ion content in major organs and tumors. [DDP] = 5 mg/kg, [bCCM] = 3 mg/kg. [bCCM + DDP] = 3 mg/kg + 2.5 mg/kg. (*n* ≥ 3; error bars represent SD, *p ≤ 0.05; ** *p* ≤ 0.01; *** *p* ≤ 0.001; **** *p* ≤ 0.0001; ns, not significant)

After confirming the excellent biosafety of bCCM, a biodistribution study was performed on a DDP–resistant animal model of HCC (BEL7402/DDP). First, IR783–labeled bCCM (bCCM@IR783) was prepared for monitoring biodistribution in the body (Fig. 6A). The fluorescence signal of mice injected with bCCM@IR783 via the tail vein was monitored via an in vivo imaging system instrument. The results revealed a fluorescence signal at the tumor site in the mice after the injection of bCCM@IR783 for 8 h. The fluorescence intensity at the tumor site continuously increased with time and peaked at approximately 12 h. Notably, there was a strong fluorescence signal at the tumor site even at 24 h after bCCM@IR783 injection, indicating fast accumulation and long retention of bCCM@IR783 at the tumor site (Fig. 6B). After 24 h, the mice were sacrificed, and the ex vivo biodistribution of bCCM@IR783 was studied. The results revealed that the fluorescence signal intensity was the strongest in the tumors, which was greater than that in the livers, kidneys, hearts, lungs, spleens, and intestines, indicating that bCCM@IR783 performed well at tumor targeting (Fig. 6C). In summary, bCCM@IR783 can rapidly target and accumulate at tumor sites, which is very conducive to its further application.

Furthermore, a biodistribution analysis of copper ions was conducted in this study. Copper ion levels in tumor tissues and major organs (heart, liver, spleen, lung, and kidney) were measured using a copper detection kit. The results showed no significant changes in copper ion levels in the major organs of the bCCM and bCCM + DDP groups (Fig. 6D). This indicates selective accumulation of copper at the tumor site, consistent with the known ability of copper carriers to enrich copper ions in tumors*–*though the degree of enrichment can vary with carrier type and experimental conditions, with some studies reporting increases up to 4.2-fold [58]. Together, these results suggest that bCCM selectively delivers copper ions to tumors, thereby supporting cuproptosis induction in vivo.

### In vivo anti–resistant effect of bCCM on HCC

**Fig. 7:**
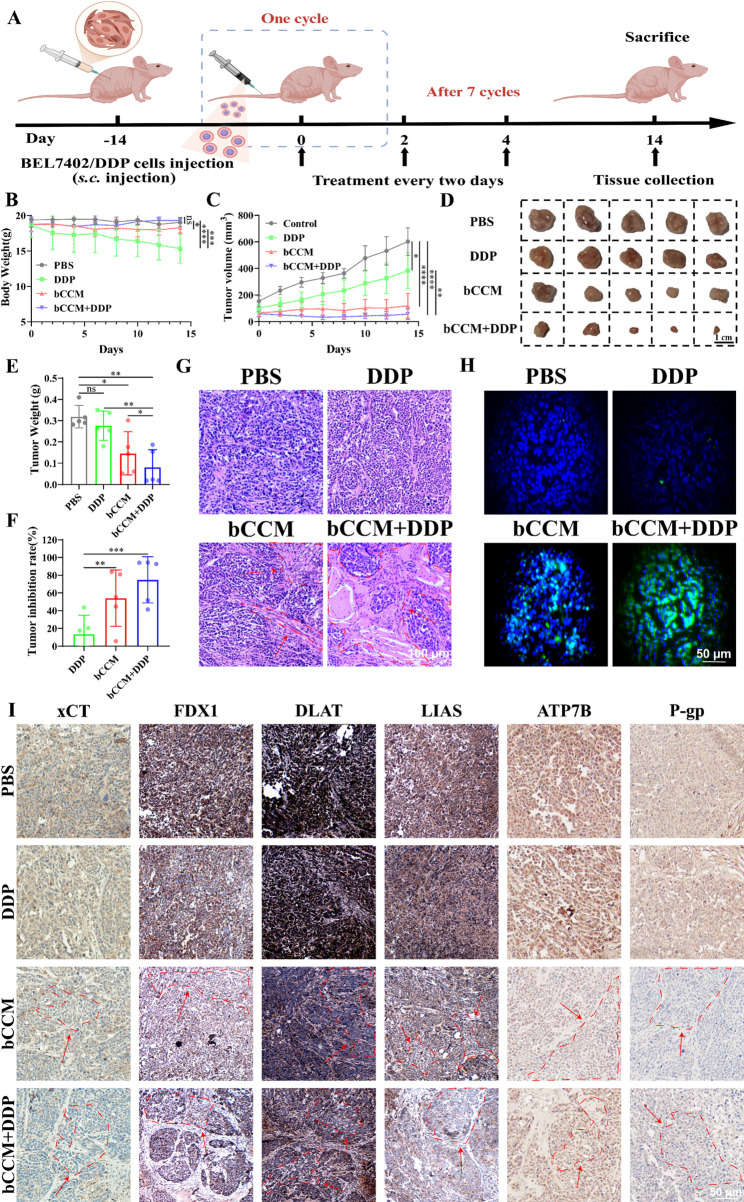
In vivo antidrug–resistant tumor efficacy and cuproptosis–associated mechanism investigation in BEL7402/DDP tumor–bearing mice. (**A**) Therapy schedule of different treatments (*n* = 5 per group). (**B**) Images of isolated tumors. Scale bar, 1 cm. (**C**) Body weights of the mice during the treatments. (**D**) Relative tumor volume changes during therapy. (**E**) Weights of isolated tumors. (**F**) Tumor inhibition rates (TIRs) of different treatments. (**G**, **H**) H&E and TUNEL staining of tumors from different treatment groups. Scale bars, 100 μm and 50 μm. (**I**) FDX1, DLAT, ATP7B and P–gp staining images of tumor tissues after different treatments for 14 days. Scale bar, 50 μm. [DDP] = 5 mg/kg, [bCCM] = 3 mg/kg. [bCCM **+** DDP] = 3 mg/kg + 2.5 mg/kg. (*n* = 5; error bars represent SD, *p ≤ 0.05; ** *p* ≤ 0.01; *** *p* ≤ 0.001; **** *p* ≤ 0.0001; ns, not significant)

Encouraged by the excellent in vitro antidrug resistance capability and enhanced cuproptosis effect of bCCM, we next sought to explore the in vivo anticancer efficacy in a cisplatin–resistant HCC mouse model. A BEL7402/DDP tumor model (five mice per group) was established, and the drug was administered via intravenous injection (i.j.) according to the treatment schedule (Fig. 7A). Based on the tumor-targeting capacity of bCCM, the improved bioavailability conferred by erythrocyte membrane modification, and supporting evidence from prior studies[47] and our in vitro results, we adopted the following dosing regimen: cisplatin monotherapy (5 mg/kg), bCCM monotherapy (3 mg/kg), and combination therapy (bCCM 3 mg/kg + cisplatin 2.5 mg/kg). This design aims to maximize therapeutic synergy while minimizing potential toxicity.

First, the body weight changes of the mice over 14 days of treatment were monitored. Negligible changes in body weight were observed in the bCCM and bCCM + DDP treatment groups compared with the control, whereas a significant decrease was noted in the DDP–treated group, indicating the good biosafety of bCCM (Fig. 7B). As shown in Fig. 7C, BEL7402/DDP tumor growth was effectively inhibited by bCCM and bCCM + DDP treatments, whereas DDP did not inhibit tumor growth, highlighting the superior anticancer effect of bCCM on drug–resistant HCC, as verified by tumor images (Fig. 7D). The bCCM + DDP groups presented the lowest tumor weight and highest tumor inhibitory ratio (TIR) at the given dose (3 mg/kg + 2.5 mg/kg), in contrast to the slight reduction in tumor size and TIR caused by DDP (5 mg/kg), suggesting poor efficiency of DDP and excellent therapeutic efficiency of bCCM in resistant HCC tumors (Fig. 7E and F).

Additionally, histological and immunohistochemical analyses were carried out to confirm the antitumor effect of bCCM (Fig. 7G). Hematoxylin and eosin (H&E) staining revealed significant tumor cell destruction in the bCCM and bCCM + DDP groups, as evidenced by massive nuclear deletions and noticeable karyopyknosis, whereas the DDP groups exhibited almost normal tumor cell morphology compared with the control. Terminal deoxynucleotidyl transferase–mediated nick end labeling (TUNEL) staining further confirmed the H&E results, revealing negligible apoptosis in the DDP group, whereas severe apoptosis and tumor growth inhibition were observed in the bCCM and bCCM + DDP groups (Fig. 7H and Fig. S35). These results demonstrated that bCCM administration led to excellent antitumor efficacy in resistant HCC, whereas limited efficacy was observed in the DDP group. The superior anti–resistance efficacy of bCCM in drug–resistant HCC mice may be attributed to the effects of cuproptosis. Subsequently, immunohistochemical staining and western blotting of tumor tissues were performed to explore the mechanism underlying the resistance capability of bCCM via cuproptosis. Immunohistochemistry revealed lower expression levels of cuproptosis–related factors (FDX1, DLAT and ATP7B) and drug resistance–related factors (ATP7B and P–gp) in the bCCM and bCCM + DDP groups than in the control group (Fig. 7I). Overall, bCCM demonstrated excellent resistance due to its ability to effectively potentiate cuproptosis.

## Discussion

As a malignant tumor with high global incidence and extremely poor prognosis[59], hepatocellular carcinoma (HCC) faces a core bottleneck in clinical treatment due to its complex and dynamically evolving drug resistance mechanisms. The mechanisms underlying tumour drug resistance manifest primarily across several key domains: clonal evolution and adaptive selection driven by tumour heterogeneity[60], drug efflux and detoxification systems exemplified by P-glycoprotein and glutathione metabolism[61], abnormal sustained activation of key signalling pathways such as Wnt/β-catenin and PI3K/AKT[62] and protective barriers formed by immunosuppressive cells and fibrotic stroma within the tumour microenvironment [63]. The interplay of these mechanisms not only limits the efficacy of current chemotherapy treatments but also hinders breakthroughs in achieving long-term patient survival.

Consequently, overcoming drug resistance in hepatocellular carcinoma has become one of the most pressing challenges. Novel therapeutic strategies are required to combat resistance in hepatocellular carcinoma. For instance, targeting novel cell death mechanisms such as copper-mediated death may circumvent the resistance pathways associated with conventional chemotherapy[64], utilizing nanocarriers enables tumor-specific enrichment and controlled drug release [65]. To address these challenges, we have developed a novel mitochondria-targeting and glutathione (GSH)-responsive copper-based nanodelivery system, designated bCCM, which demonstrates efficient targeting of cisplatin-resistant hepatocellular carcinoma cells. bCCM forms a stable Cu (II) chelate through a tridentate coordination structure and is further modified with a red blood cell membrane to enhance biocompatibility and tumor-targeting capability. The system operates via a triple-pronged synergistic therapeutic strategy: Firstly, it achieves high-capacity, controlled copper release through Cu (II)-mediated Fenton reactions and Michael addition. Secondly, it suppresses GSH biosynthesis by inhibiting xCT protein while simultaneously promoting GSH depletion, thereby comprehensively exhausting intracellular GSH levels in tumor cells. Thirdly, it triggers cuproptosis, leading to alterations in mitochondrial membrane potential and downregulation of ATP7B and P-gp expression, effectively inhibiting the efflux of both copper ions and cisplatin and synergistically reversing multidrug resistance. Comprehensive in vitro and in vivo safety evaluations demonstrate that bCCM exhibits favorable biosafety profiles in normal hepatocytes (AML-12), hepatic stellate cells (LX-2), zebrafish embryos, and mouse models, highlighting its promising potential for clinical translation.

## Conclusions

In conclusion, we have developed a novel mitochondrion–targeted and GSH–responsive copper carrier, bCCM, that effectively targets cisplatin–resistant hepatocellular carcinoma. This supramolecular complex, formed by chelating bm–Cur with Cu(II) at three coordination sites and encapsulating it with RBC membranes, addresses the key limitations of cuproptosis therapy by enhancing copper delivery through the tridentate chelation architecture, which enables high–capacity copper loading and targeted delivery, comprehensive GSH depletion achieved by dual inhibition of GSH synthesis and consumption via Cu(II)–mediated Fenton reactions and Michael addition, and resistance modulation through downregulation of ATP7B to prevent copper/cisplatin efflux. In summary, this work establishes bCCM as a first–in–class therapeutic paradigm for the effective treatment of platinum–resistant malignancies, which also serves as an important example for new applications of traditional Chinese medicine.

## Supplementary Information

Supplementary Material 1.

## Data Availability

No datasets were generated or analysed during the current study.

## Abbreviations

ATP7B: ATPase copper transporting beta
bm-Cur: Bisdemethylcurcumin
β-actin: Beta-actin
CCK-8: Cell Counting kit-8
CLSM: Confocal Laser Scanning Microscopy
DCFH-DA: Dichlorodihydrofluorescein Diacetate
DAPI: Diamidino-2-phenylindole
DDP: cis-Diaminodichloroplatinum
DLAT: Dihydrolipoamide S-acetyltransferase
DMSO: Dimethyl Sulfoxide
DMEM: Duchenne’s Modified Eagle Medium
DMEM/F-12: Dulbecco’s Modified Eagle Medium/Nutrient Mixture F-12
FBS: Fetal Bovine Serum
FDX1: Ferredoxin-1
Fer-1: Ferrostatin-1
GSH: Glutathione
GPx4: Glutathione Peroxidase 4
HCC: Hepatocellular Carcinoma
HRLC-MS: High Resolution Liquid Chromatography-Mass Spectrometry
^1^H NMR: Nuclear Magnetic Resonance Hydrogen Spectrum
LIAS: Lipoic Acid Synthetase
LPO: Lipid Peroxide
LRLC-MS: Low Resolution Liquid Chromatography-Mass Spectrometry
PBS: Phosphate Buffered Saline
P-gp: P-glycoprotein
ROS: Reactive Oxygen Species
TCA: Tricarboxylic Acid Cycle
TTM: Tetrathiomolybdate
xCT: solute carrier family 7 member 11

## References

[1] Vasan N, Baselga J, Hyman DM. A view on drug resistance in cancer. Nature. 2019;575(7782):299–309. 31723286 10.1038/s41586-019-1730-1 PMC8008476

[2] TADINI-BUONINSEGNI F, BARTOLOMMEI G, MONCELLI M R, et al. Translocation of platinum anticancer drugs by human copper ATPases ATP7A and ATP7B . Angew Chem Int Ed Engl. 2014;53(5):1297–1301. 10.1002/anie.201307718 PMC3937162 24375922

[3] JANARDHANAN P, SOMASUNDARAN A K, BALAKRISHNAN A J, et al. Sensitization of cancer cells towards Cisplatin and Carboplatin by protein kinase D inhibitors through modulation of ATP7A/B (copper transport ATPases) . Cancer Treat Res Commun. 2022;32:100613. 10.1016/j.ctarc.2022.100613 35908410

[4] Riordan JR, Deuchars K, Kartner N, et al. Amplification of P-glycoprotein genes in multidrug-resistant mammalian cell lines. Nature. 1985;316(6031):817–9. 2863759 10.1038/316817a0

[5] Lobo J, JERóNIMO C, Henrique R. Cisplatin resistance in testicular germ cell tumors: current challenges from various perspectives. Cancers (Basel). 2020;12(6):1601. 32560427 10.3390/cancers12061601 PMC7352163

[6] Xiao X, Wang K, Zong Q, et al. Polyprodrug with glutathione depletion and cascade drug activation for multi-drug resistance reversal. Biomaterials. 2021;270:120649. 33588139 10.1016/j.biomaterials.2020.120649

[7] Xie K, Doles J, Hemann MT, et al. Error-prone translesion synthesis mediates acquired chemoresistance. Proc Natl Acad Sci U S A. 2010;107(48):20792–7. 21068378 10.1073/pnas.1011412107 PMC2996453

[8] SHAO N, YUAN L, LIU L, et al. Reversing Anticancer Drug Resistance by Synergistic Combination of Chemotherapeutics and Membranolytic Antitumor β-Peptide Polymer . J Am Chem Soc. 2024; advance online publication. 10.1021/jacs.4c00434 10.1021/jacs.4c00434 38602146

[9] TSVETKOV P, COY S, PETROVA B, et al. Copper induces cell death by targeting lipoylated TCA cycle proteins . Science. 2022;375(6586):1254–1261. 10.1126/science.abf0529 PMC9273333 35298263

[10] Huang L, Zhu J, Wu G, et al. A strategy of “adding fuel to the flames” enables a self-accelerating cycle of ferroptosis-cuproptosis for potent antitumor therapy. Biomaterials. 2024;311:122701. 38981152 10.1016/j.biomaterials.2024.122701

[11] LIU C, GUO L, CHENG Y, et al. A Mitochondria-Targeted Nanozyme Platform for Multi-Pathway Tumor Therapy via Ferroptosis and Cuproptosis Regulation . Adv Sci (Weinh). 2025;e17616. 10.1002/advs.202417616 PMC12462976 40558386

[12] LU Y, PAN Q, GAO W, et al. Reversal of cisplatin chemotherapy resistance by glutathione-resistant copper-based nanomedicine via cuproptosis . J Mater Chem B. 2022;10(33):6296–6306. 10.1039/d2tb01150f 35904024

[13] Wang Y, Chen Y, Zhang J, et al. Cuproptosis: a novel therapeutic target for overcoming cancer drug resistance. Drug Resist Updat. 2024;72:101018. 37979442 10.1016/j.drup.2023.101018

[14] Yao L, Jiang B, Xu D. Strategies to combat cancer drug resistance: focus on copper metabolism and cuproptosis. Cancer Drug Resist. 2025;8:15. 40201308 10.20517/cdr.2025.41 PMC11977383

[15] SOLIER S, MüLLER S, CAñEQUE T, et al. A druggable copper-signalling pathway that drives inflammation . Nature. 2023;617(7960):386–394. 10.1038/s41586-023-06017-4 PMC10131557 37100912

[16] Xie J, Yang Y, Gao Y, et al. Cuproptosis: mechanisms and links with cancers. Mol Cancer. 2023;22(1):46. 36882769 10.1186/s12943-023-01732-y PMC9990368

[17] NING S, LYU M, ZHU D, et al. Type-I AIE Photosensitizer Loaded Biomimetic System Boosting Cuproptosis to Inhibit Breast Cancer Metastasis and Rechallenge . ACS Nano. 2023;17(11):10206–10217. 10.1021/acsnano.3c00326 37183977

[18] SUN L, ZHANG Y, YANG B, et al. Lactylation of METTL16 promotes cuproptosis via m(6)A-modification on FDX1 mRNA in gastric cancer . Nat Commun. 2023;14(1):6523. 10.1038/s41467-023-42025-8 PMC10589265 37863889

[19] XU Y, LIU S Y, ZENG L, et al. An Enzyme-Engineered Nonporous Copper(I) Coordination Polymer Nanoplatform for Cuproptosis-Based Synergistic Cancer Therapy . Adv Mater. 2022;34(43):e2204733. 10.1002/adma.202204733 36054475

[20] LIU Y, NIU R, ZHAO H, et al. Single-Site Nanozymes with a Highly Conjugated Coordination Structure for Antitumor Immunotherapy via Cuproptosis and Cascade-Enhanced T Lymphocyte Activity . J Am Chem Soc. 2024;146(6):3675–3688. 10.1021/jacs.3c08622 38305736

[21] Zhou J, Yu Q, Song J, et al. Photothermally triggered copper payload release for cuproptosis-promoted cancer synergistic therapy. Angew Chem Int Ed Engl. 2023;62(12):e202213922. 36585379 10.1002/anie.202213922

[22] Xiong C, Ling H, Hao Q, et al. Cuproptosis: p53-regulated metabolic cell death? Cell Death Differ. 2023;30(4):876–84. 36755067 10.1038/s41418-023-01125-0 PMC10070433

[23] HARRIS I S, TRELOAR A E, INOUE S, et al. Glutathione and thioredoxin antioxidant pathways synergize to drive cancer initiation and progression . Cancer Cell. 2015;27(2):211–222. 10.1016/j.ccell.2014.11.019 25620030

[24] Zhang S, Xie X, Liu M, et al. Biomimetic Ti3C2 nanosheets for synergistically overcoming chemoresistance and boosting immunotherapy via enhanced cuproptosis in cancer. Chem Eng J. 2025;520:165766.

[25] Zhu G, Xie Y, Wang J, et al. Multifunctional copper-phenolic nanopills achieve comprehensive polyamines depletion to provoke enhanced pyroptosis and cuproptosis for cancer immunotherapy. Adv Mater. 2024;36(45):e2409066. 39285820 10.1002/adma.202409066

[26] Wu Z, Lv G, Xing F, et al. Copper in hepatocellular carcinoma: a double-edged sword with therapeutic potentials. Cancer Lett. 2023;571:216348. 37567461 10.1016/j.canlet.2023.216348

[27] Niu B, Liao K, Zhou Y, et al. Application of glutathione depletion in cancer therapy: enhanced ROS-based therapy, ferroptosis, and chemotherapy. Biomaterials. 2021;277:121110. 34482088 10.1016/j.biomaterials.2021.121110

[28] JIANG W, LUO X, WEI L, et al. The Sustainability of Energy Conversion Inhibition for Tumor Ferroptosis Therapy and Chemotherapy . Small. 2021;17(38):e2102695. 10.1002/smll.202102695 34350694

[29] GE E J, BUSH A I, CASINI A, et al. Connecting copper and cancer: from transition metal signalling to metalloplasia . Nat Rev Cancer. 2022;22(2):102–113. 10.1038/s41568-021-00417-2 PMC8810673 34764459

[30] Quamar S, Kumar J, Mishra A, et al. Oxidative stress and neurobehavioural changes in rats following copper exposure and their response to MiADMSA and d-penicillamine. Toxicol Res Appl. 2019;3:1–15.

[31] Oliveri V. Selective targeting of cancer cells by copper ionophores: an overview. Front Mol Biosci. 2022;9:841814. 35309510 10.3389/fmolb.2022.841814 PMC8931543

[32] STEINBRUECK A, SEDGWICK A C, BREWSTER J T, 2ND, et al. Transition metal chelators, pro-chelators, and ionophores as small molecule cancer chemotherapeutic agents . Chem Soc Rev. 2020;49(12):3726–3747. 10.1039/c9cs00373h 32525153

[33] Oliveri V. Biomedical applications of copper ionophores. Coord Chem Rev. 2020;422:213474.

[34] Nagai M, Vo NH, Shin Ogawa L, et al. The oncology drug elesclomol selectively transports copper to the mitochondria to induce oxidative stress in cancer cells. Free Radic Biol Med. 2012;52(10):2142–50. 22542443 10.1016/j.freeradbiomed.2012.03.017

[35] HAMZA M, WANG S, WU H, et al. Targeting copper homeostasis: Akkermansia-derived OMVs co-deliver Atox1 siRNA and elesclomol for cancer therapy . Acta Pharm Sin B. 2025;15(5):2640–2654. 10.1016/j.apsb.2025.03.014 PMC12145039 40487636

[36] KANG X, JADHAV S, ANNAJI M, et al. Advancing Cancer Therapy with Copper/Disulfiram Nanomedicines and Drug Delivery Systems . Pharmaceutics. 2023;15(6):1567. 10.3390/pharmaceutics15061567 PMC10302862 37376016

[37] Lee BH, Song E, Hong J. Interaction of thiol antioxidants with α,β-unsaturated ketone moiety: its implication for stability and bioactivity of curcuminoids. Molecules. 2023;28(23):7711. 38067442 10.3390/molecules28237711 PMC10707499

[38] Mohanty C, Sahoo SK. The in vitro stability and in vivo pharmacokinetics of curcumin prepared as an aqueous nanoparticulate formulation. Biomaterials. 2010;31(25):6597–611. 20553984 10.1016/j.biomaterials.2010.04.062

[39] AWASTHI S, PANDYA U, SINGHAL S S, et al. Curcumin-glutathione interactions and the role of human glutathione S-transferase P1-1 . Chem Biol Interact. 2000;128(1):19–38. 10.1016/s0009-2797(00)00185-x 10996298

[40] PIPER J T, SINGHAL S S, SALAMEH M S, et al. Mechanisms of anticarcinogenic properties of curcumin: the effect of curcumin on glutathione linked detoxification enzymes in rat liver . Int J Biochem Cell Biol. 1998; 30(4): 445–456. 10.1016/s1357-2725(98)00015-6 9675878

[41] Chen WH, Chen QW, Chen Q, et al. Biomedical polymers: synthesis, properties, and applications. Sci China Chem. 2022;65(6):1010–75. 35505924 10.1007/s11426-022-1243-5 PMC9050484

[42] Xu T, Ma Q, Zhang C, et al. A novel nanomedicine for osteosarcoma treatment: triggering ferroptosis through GSH depletion and inhibition for enhanced synergistic PDT/PTT therapy. J Nanobiotechnology. 2025;23(1):323. 40301915 10.1186/s12951-025-03380-4 PMC12039277

[43] SATHYABHAMA M, PRIYA DHARSHINI L C, KARTHIKEYAN A, et al. The Credible Role of Curcumin in Oxidative Stress-Mediated Mitochondrial Dysfunction in Mammals . Biomolecules. 2022;12(10):1405. 10.3390/biom12101405 PMC9599178 36291614

[44] ZHANG M, XU H, WU X, et al. Engineering Dual-Responsive Nanoplatform Achieves Copper Metabolism Disruption and Glutathione Consumption to Provoke Cuproptosis/Ferroptosis/Apoptosis for Cancer Therapy . ACS Appl Mater Interfaces. 2025;17(14):20726–20740. 10.1021/acsami.4c22546 40134095

[45] LIU J, YUAN Y, CHENG Y, et al. Copper-Based Metal-Organic Framework Overcomes Cancer Chemoresistance through Systemically Disrupting Dynamically Balanced Cellular Redox Homeostasis . J Am Chem Soc. 2022;144(11):4799–4809. 10.1021/jacs.1c11856 35192770

[46] ZHU J, WANG X, SU Y, et al. Multifunctional nanolocks with GSH as the key for synergistic ferroptosis and anti-chemotherapeutic resistance . Biomaterials. 2022;288:121704. 10.1016/j.biomaterials.2022.121704 35948496

[47] MAO L, QIN Y, FAN J, et al. Rapid discovery of a novel “green” and natural GST inhibitor for sensitizing hepatocellular carcinoma to Cisplatin by visual screening strategy . J Pharm Anal. 2024;14(5):100923. 10.1016/j.jpha.2023.12.013 PMC11127223 38799232

[48] HANS C, SAINI R, SACHDEVA M U S, et al. 2’,7’-Dichlorofluorescein (DCF) or 2’,7’-dichlorodihydrofluorescein diacetate (DCFH2-DA) to measure reactive oxygen species in erythrocytes . Biomed Pharmacother. 2021;138:111512. 10.1016/j.biopha.2021.111512 33743333

[49] LIU S, ZHANG N, JI X, et al. Helicobacter pylori CagA promotes gastric cancer immune escape by upregulating SQLE . Cell Death Dis. 2025;16(1):17. 10.1038/s41419-024-07318-w PMC11733131 39809787

[50] PAGANO C, NAVARRA G, COPPOLA L, et al. N6-isopentenyladenosine induces cell death through necroptosis in human glioblastoma cells . Cell Death Discov. 2022;8(1):173. 10.1038/s41420-022-00974-x PMC8991250 35393392

[51] Chen L, Min J, Wang F. Copper homeostasis and cuproptosis in health and disease. Signal Transduct Target Ther. 2022;7(1):378. 36414625 10.1038/s41392-022-01229-y PMC9681860

[52] Tadini-Buoninsegni F, Smeazzetto S. Mechanisms of charge transfer in human copper ATPases ATP7A and ATP7B. IUBMB Life. 2017;69(4):218–25. 28164426 10.1002/iub.1603

[53] Ye J, Hou F, Chen G, et al. Novel copper-containing ferrite nanoparticles exert lethality to MRSA by disrupting MRSA cell membrane permeability, depleting intracellular iron ions, and upregulating ROS levels. Front Microbiol. 2023;14:1023036. 36846790 10.3389/fmicb.2023.1023036 PMC9947852

[54] Tang D, Chen X, Kroemer G. Cuproptosis: a copper-triggered modality of mitochondrial cell death. Cell Res. 2022;32(5):417–8. 35354936 10.1038/s41422-022-00653-7 PMC9061796

[55] Li Y, Liu J, Weichselbaum RR, et al. Mitochondria-targeted multifunctional nanoparticles combine cuproptosis and programmed cell death-1 downregulation for cancer immunotherapy. Adv Sci. 2024;11(35):e2403520. 10.1002/advs.202403520 PMC11425249 39013093

[56] HE W, ZHANG X-Y, GONG X, et al. Drug-Free Biomimetic Oxygen Supply Nanovehicle Promotes Ischemia-Reperfusion Therapy in Stroke . Advanced Functional Materials. 2023;33(21):2212919.

[57] Zhang D, Man D, Lu J, et al. Mitochondrial TSPO promotes hepatocellular carcinoma progression through ferroptosis inhibition and immune evasion. Adv Sci. 2023;10(15):e2206669. 10.1002/advs.202206669 PMC10214260 36994647

[58] GUO B, YANG F, ZHANG L, et al. Cuproptosis Induced by ROS Responsive Nanoparticles with Elesclomol and Copper Combined with αPD-L1 for Enhanced Cancer Immunotherapy . Adv Mater. 2023;35(22):e2212267. 10.1002/adma.202212267 36916030

[59] Bray F, Laversanne M, Sung H, et al. Global cancer statistics 2022: GLOBOCAN estimates of incidence and mortality worldwide for 36 cancers in 185 countries. CA Cancer J Clin. 2024;74(3):229–63. 38572751 10.3322/caac.21834

[60] KEENAN B P, YADAV M, ANSSTAS G, et al. Intratumoral heterogeneity and immunotherapy resistance: clinical implications . Ann Oncol, 2025, advance online publication. 10.1016/j.annonc.2025.10.1239 10.1016/j.annonc.2025.10.1239 41203206

[61] Sun J, An X, Wang Y, et al. A hyaluronic acid modified copper-based metal-organic framework overcomes multidrug resistance via two-way redox dyshomeostasis under hypoxia. Int J Biol Macromol. 2025;300:140148. 39848376 10.1016/j.ijbiomac.2025.140148

[62] LIM S H, LEE H, LEE H J, et al. PLD1 is a key player in cancer stemness and chemoresistance: Therapeutic targeting of cross-talk between the PI3K/Akt and Wnt/β-catenin pathways . Exp Mol Med. 2024;56(7):1479–1487. 10.1038/s12276-024-01260-9 PMC11297275 38945955

[63] LIU C, XIA S, WANG B, et al. Osteopontin promotes tumor microenvironment remodeling and therapy resistance . Cancer Lett. 2025;617:217618. 10.1016/j.canlet.2025.217618 40058726

[64] Lei G, Sun M, Cheng J, et al. Radiotherapy promotes cuproptosis and synergizes with cuproptosis inducers to overcome tumor radioresistance. Cancer Cell. 2025;43(6):1076-1092.e5. 40215978 10.1016/j.ccell.2025.03.031 PMC12151758

[65] Cheng H, Liao J, Ma Y, et al. Advances in targeted therapy for tumor with nanocarriers: a review. Mater Today Bio. 2025;31:101583. 40061211 10.1016/j.mtbio.2025.101583 PMC11889621

