# Supplementary material for: A mitochondrion–targeted natural polyphenolic copper carrier overcomes tumor resistance to cisplatin by potentiating cuproptosis
Source: J Exp Clin Cancer Res. 2026 Jan 20;45:53. doi: 10.1186/s13046-026-03642-5 (PMC12905869; doi:10.1186/s13046-026-03642-5)
Supplement: Supplementary file 1 — Supplementary Material 1. [file 13046_2026_3642_MOESM1_ESM.docx]

**A mitochondrion–targeted natural polyphenolic copper carrier overcomes tumor resistance to cisplatin by potentiating cuproptosis**

Haoyu Yang^a#^, Xiang Xiong^b#^, Xin Chen^a^, Siqi Huang^a^, Hongfang Dai^a^, Liqin Yuan^b*^, Jialong Fan ^c^, Zhenhong Xiang ^a^, Wei Wang ^a*^, Yan Qin ^a*^

^a^ School of Pharmacy, Hunan University of Chinese Medicine, Changsha, Hunan, 410208, P.R. China

^b^ The Second Xiangya Hospital, Central South University, Changsha, Hunan, 410011, P.R. China

^c^ Hunan Provincial Key Laboratory of the Research and Development of Novel Pharmaceutical Preparations, Changsha Medical University, Changsha, China

^#^ These authors contributed equally to this work and should be regarded as the first authors

^*^ To whom correspondence should be addressed. Tel: +86–731–88458223;

E–mail: Yan Qin (email, [qin86yan@hotmail.com](mailto:qin86yan@hotmail.com)); Wei Wang (email, wangwei402@hotmail. com); Liqin Yuan (email, yuanliqin@csu.edu.cn)

**Contents**

Fig. S1. The characterization of bm–Cur4

Fig. S2. HRLC–MS spectrometry of bm–Cur reaction with GSH4

**Fig. S3. The UV–vis spectra of bm–Cur reaction with GSH5**

Fig. S4. The GSH consumption capacity test of bm–Cur 5

Fig. S5. Cytotoxicity of bm–Cur on DDP–sensitive hepatocellular carcinoma cells5

Fig. S6. Cytotoxicity of bm–Cur on DDP–resistant hepatocellular carcinoma cells6

Fig. S7. The stable half–life of bCCM in PBS and serum 6

Fig. S8. Stability test of bCCM 7

Fig. S9. The UV–vis spectra after the reaction of bCCM with different concentrations of GSH 7

Fig. S10. bm–Cur content detection in bCCM 7

Fig. S11. The cell viability of BEL7402/DDP cells treated with different concentrations of bCC and bCCM8

Fig. S12. The cell viability of different cells treated with DDP8

Fig. S13. The cell viability of different cells treated with bCCM8

Table. S1. IC_50_ for different cells with different administrations9

Fig. S14. The CCK–8 assay of AML–12 cells treat with bCCM9

Fig. S15. The CCK–8 assay of LX–2 cells treat with bCCM and DDP9

Fig. S16. Bright field image of BEL7402/DDP cells for Live/dead staining 10

Fig. S17. Bright field image of BEL7402/DDP cells for necrosis and apoptosis10

Fig. S18. Quantification of xCT proteins in BEL7402/DDP cells10

Fig. S19. The GSH consumption capacity test of bCCM11

**Fig. S20. Detection of GSH levels with different treatments on HepG2 cells11**

**Fig. S21.** **Bright field image of BEL7402/DDP cells for intracellular copper ions levels detection11**

**Fig. S22.** **Cytotoxicity of copper ions on BEL7402/DDP cells12**

**Fig. S23.** **Cytotoxicity of UK5099 on BEL7402/DDP cells12**

**Fig. S24.** **Cell viability of BEL7402/DDP cells treated with bCCM with different concentrations of TTM12**

**Fig. S25. Quantification of drug–resistant proteins in BEL7402/DDP cells 13**

**Fig. S26. The WB of necroptosis and pyroptosis related proteins13**

**Fig. S27.** **Quantitative analysis of necroptosis–associated proteins in BEL7402/DDP cells14**

**Fig. S28. Quantitative analysis of pyroptosis–associated proteins in BEL7402/DDP cells14**

**Fig. S29. Transcriptomics analysis of BEL7402/DDP cells with bCCM treatment 15**

**Fig. S30. Mitochondrial GSH detection on HepG2 cells15**

**Fig. S31.** **Quantitative analysis of Western blot at mitochondrial level16**

**Fig. S32. Zebrafish toxicity assay17**

**Fig. S33. Hemolysis test of bCCM18**

**Fig. S34. Biosafety and biocompability assay of bCCM18**

**Fig. S35. Quantitative analysis of mean fluorescence by Tunel assay19**

**Fig. S36. The original blots of Fig. 1D20**

**Fig. S37. The original blots of Fig. 1E20**

**Fig. S38. The original blots of Fig. 2F21**

**Fig. S39. The original blots of Fig. 3G21**

**Fig. S40. The original blots of Fig. 3K22**

**Fig. S41. The original blots of Fig. 4D22**

**Fig. S42.** **The original blots of Fig. 4G23**

**Fig. S43.** **The original blots of necroptosis–associated protein23**

**Fig. S44. The original blots of pyroptosis–related proteins24**


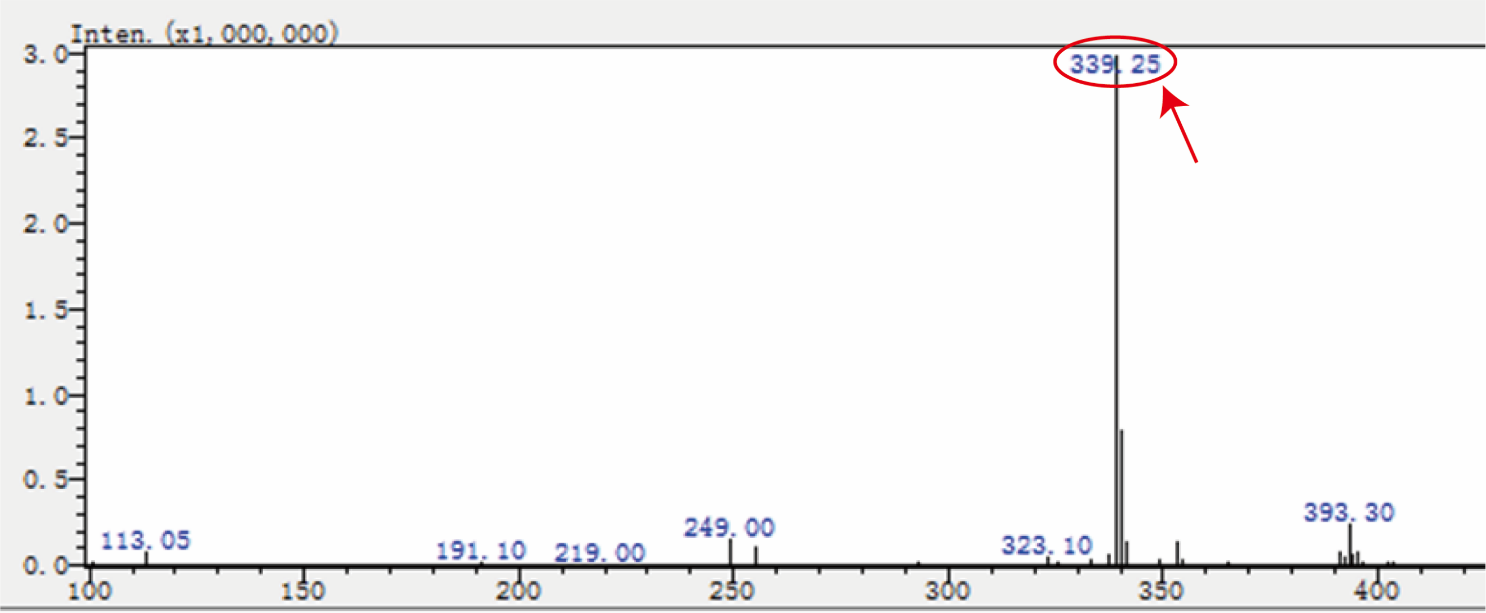


**Fig. S1.** The characterization of bm–Cur. Low resolution liquid chromatography–mass spectrometry of bm–Cur. ESI^–^ scanning for C_19_H_16_O_6_ (Mw = 340.33) detected [M – H] ^–^ at m/z = 339.25.


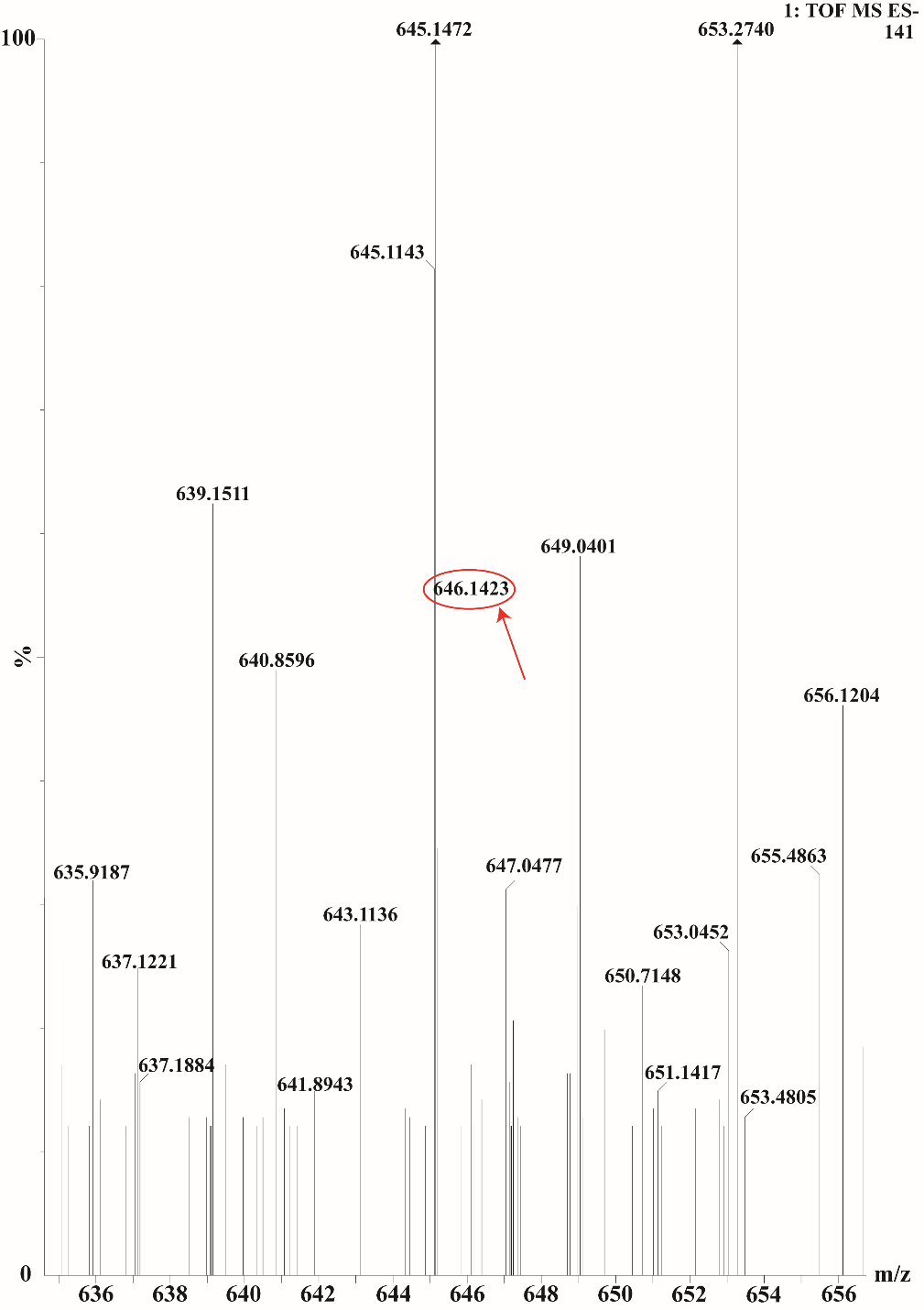


**Fig. S2.** High–resolution liquid chromatography–mass spectrometry of bm–Cur (40 μM) reaction with GSH (10 mM) for 24 h. ESI^–^ scanning for C_29_H_33_N_3_O_12_S (Mw = 647.18) detected [M – H]^-^ at m/z = 646.1423.


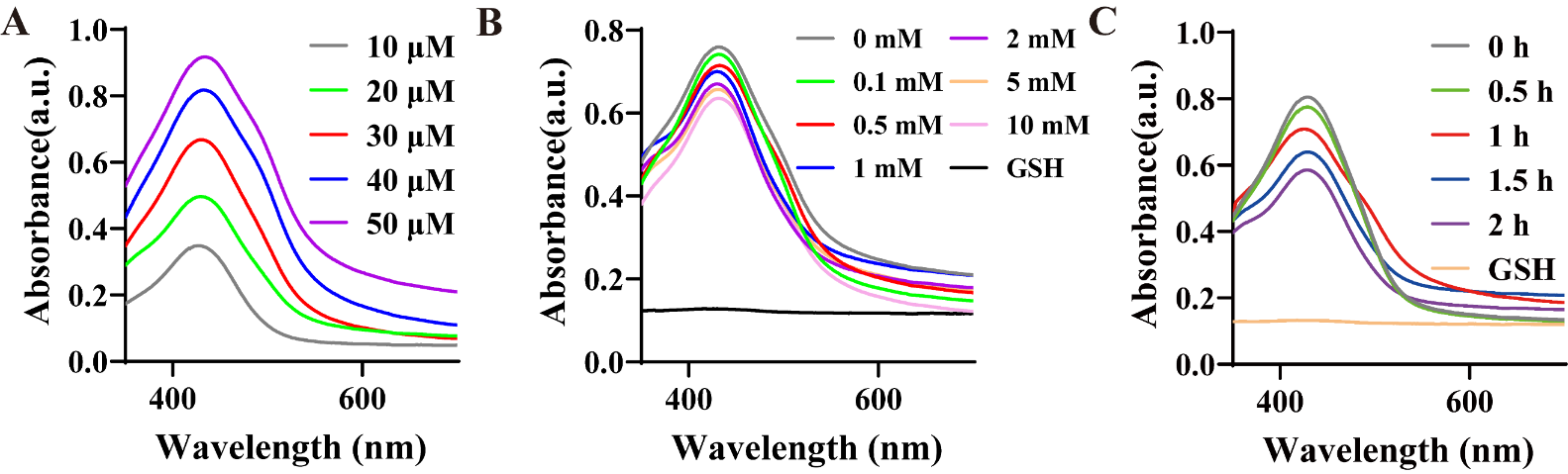


**Fig. S3.** The UV–vis spectra of bm–Cur reaction with GSH. (A) UV–vis spectra of different bm–Cur concentrations. (B) UV–vis spectra of bm–Cur (40 μM) reaction with different GSH concentrations for 1 h. (C) UV–vis spectra of bm–Cur (40 μM) reaction with 10 mM GSH over time.


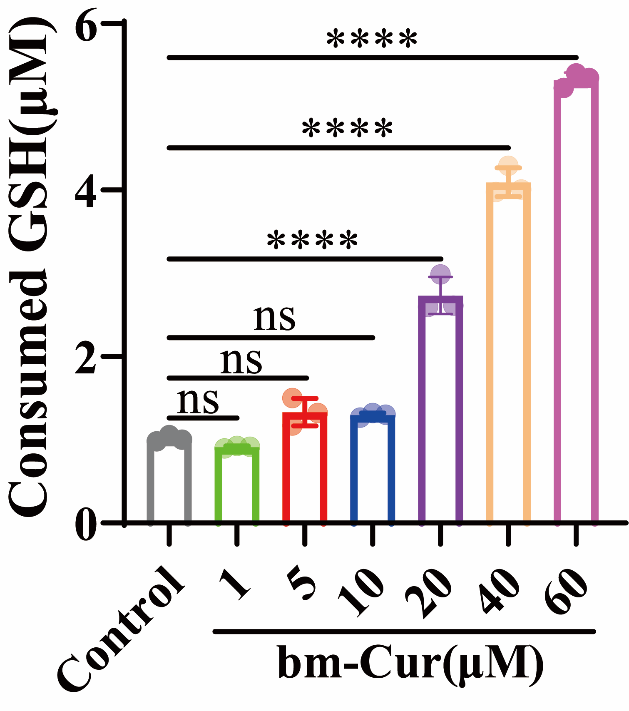


**Fig. S4.** GSH consumption capacity test of bm–Cur. The consumed GSH of BEL7402/DDP cells with different concentrations of bm–Cur treatments for 24 h. (n ≥ 3; error bars represent SD, **p* ≤ 0.05; ** *p* ≤ 0.01; *** *p* ≤ 0.001; **** *p* ≤ 0.0001).


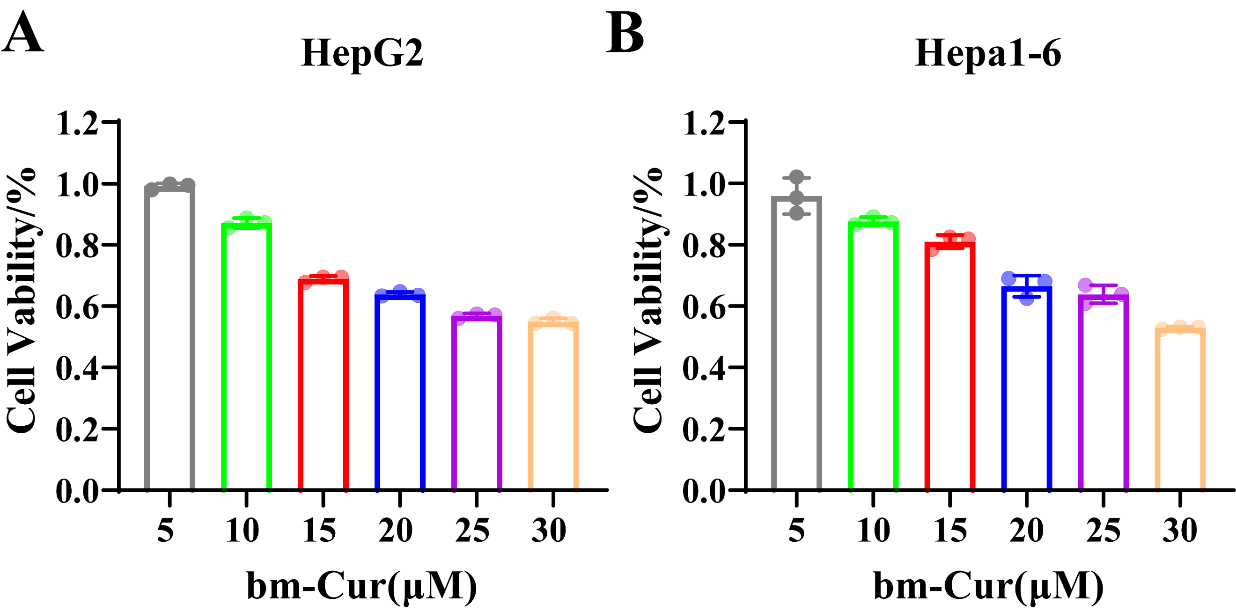


**Fig. S5.** Cytotoxicity of bm–Cur on cisplatin–sensitive hepatocellular carcinoma cells. (A–B) The cell viability of Hepatocellular carcinoma sensitive cells (HepG2 and Hepa1–6 cells) treated with different concentrations of bm–Cur for 48 h. (n ≥ 3; error bars represent SD).


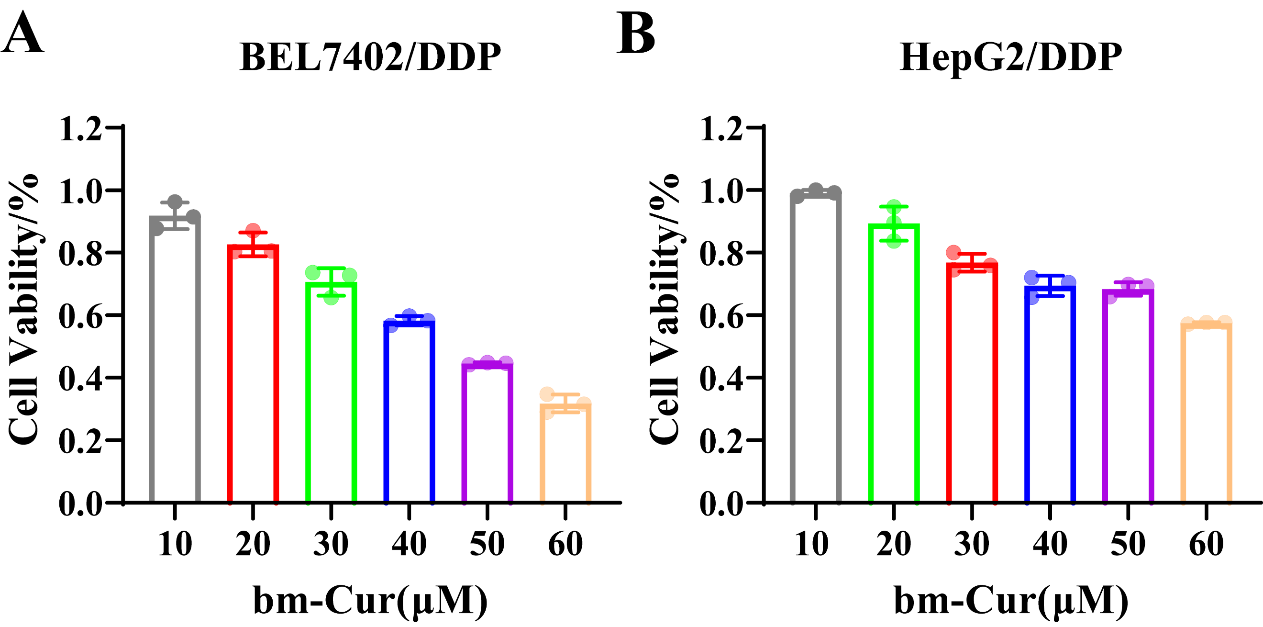


**Fig. S6.** Cytotoxicity of bm–Cur on cisplatin–resistant hepatocellular carcinoma cells. (A–B) The cell viability of Hepatocellular carcinoma cisplatin–resistant cells (BEL7402/DDP and HepG2/DDP cells) treated with different concentrations of bm–Cur for 48 h. (n ≥ 3; error bars represent SD).


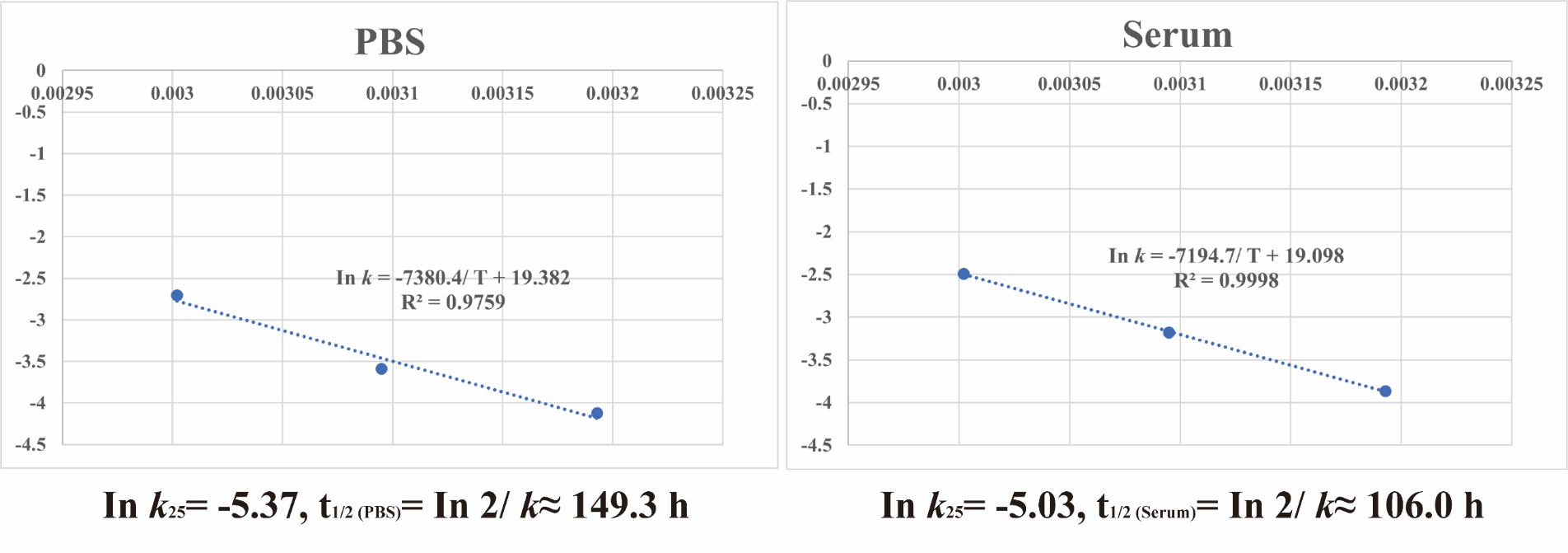


**Fig. S7.** The stable half–life of bCCM in PBS and serum.


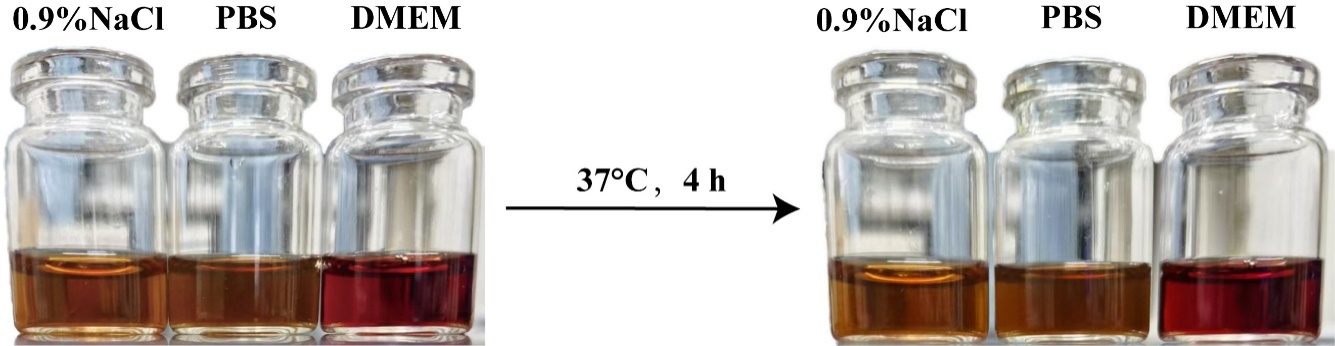


**Fig. S8.** Stability test of bCCM in various solutions.


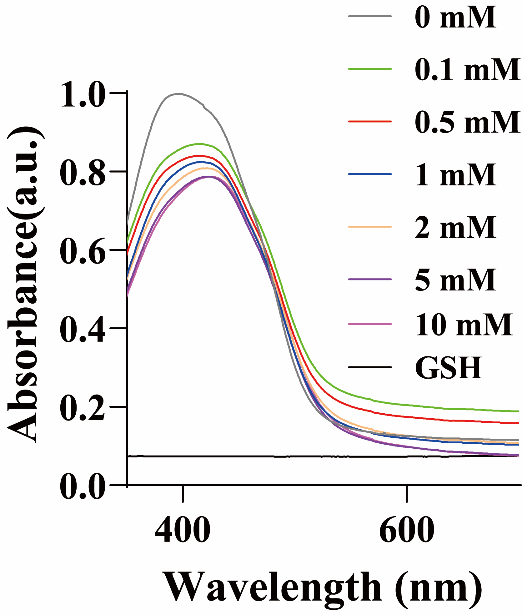


**Fig. S9.** The UV–vis spectra after the reaction of bCCM with different concentrations of GSH.


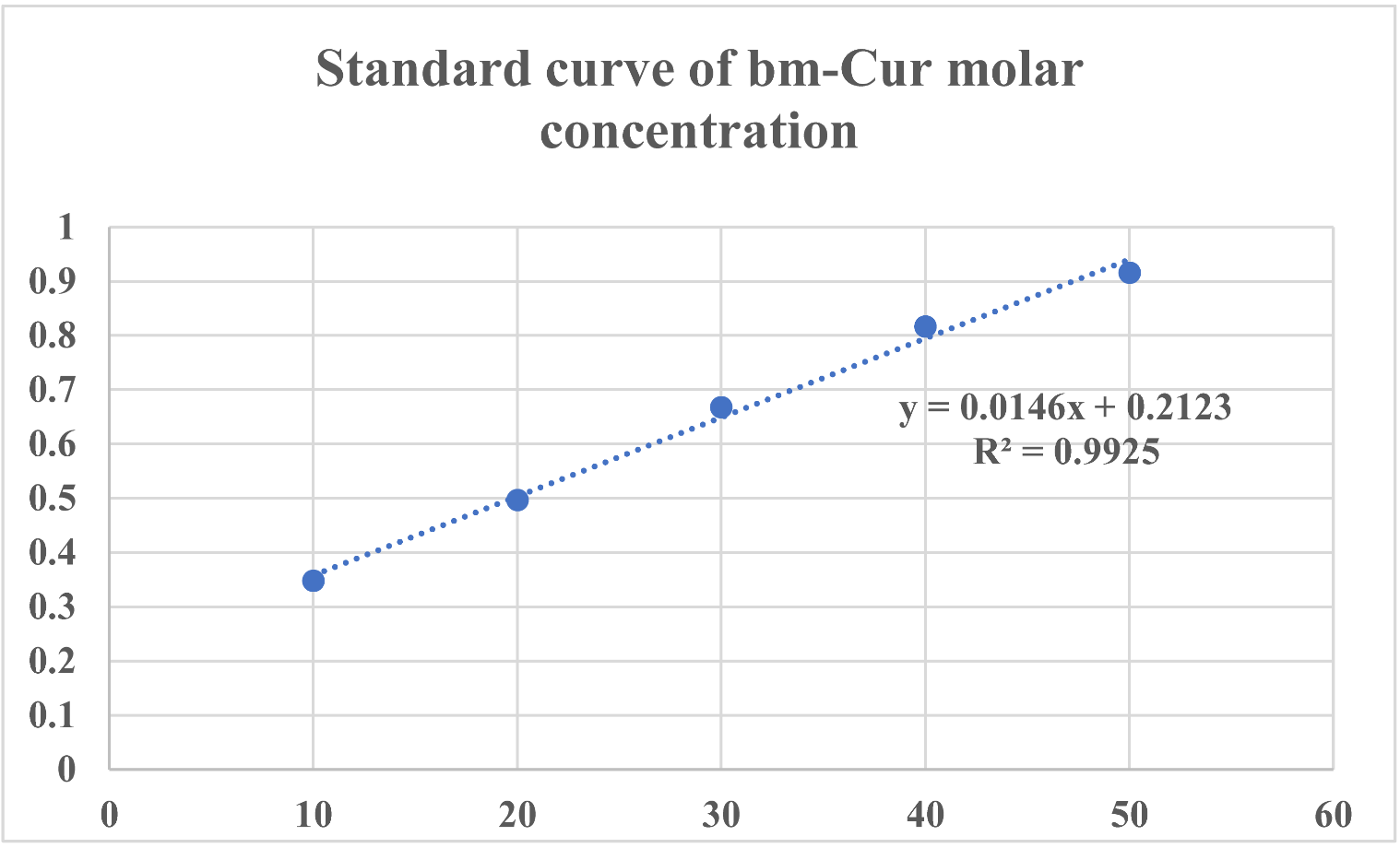


**Fig. S10.** Concentration standard curve of bm–Cur and the determination of bm–Cur content in bCCM. Releasing bm–Cur (μM) = [A430 (90 μg/mL bCCM + 10mM GSH, 2 h)–0.2123]/0.0146 = 39.22.


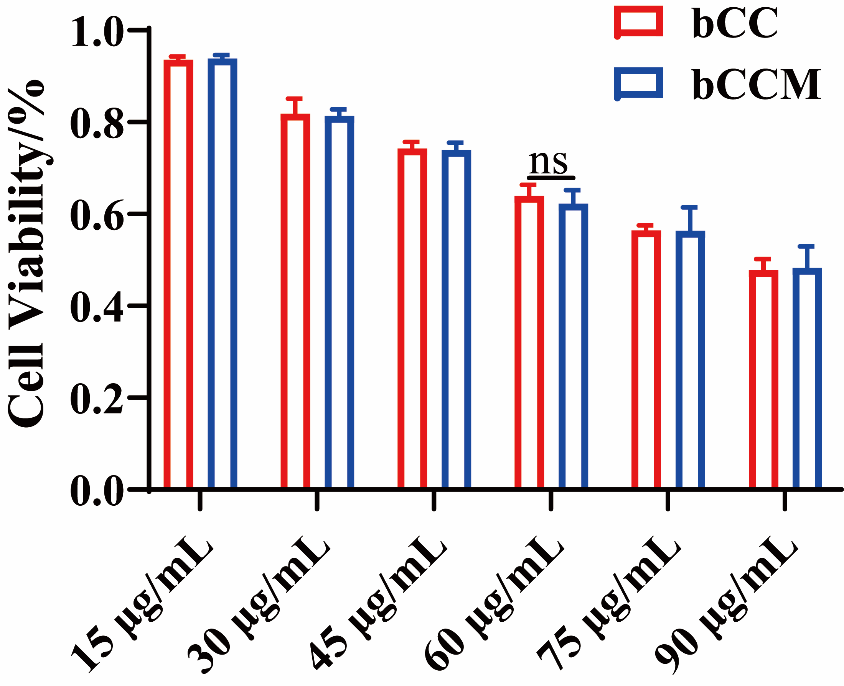


**Fig. S11.** The cell viability of BEL7402/DDP cells treated with different concentrations of bCC and bCCM. (n ≥ 3; error bars represent SD, **p* ≤ 0.05; ** *p* ≤ 0.01; *** *p* ≤ 0.001; **** *p* ≤ 0.0001).


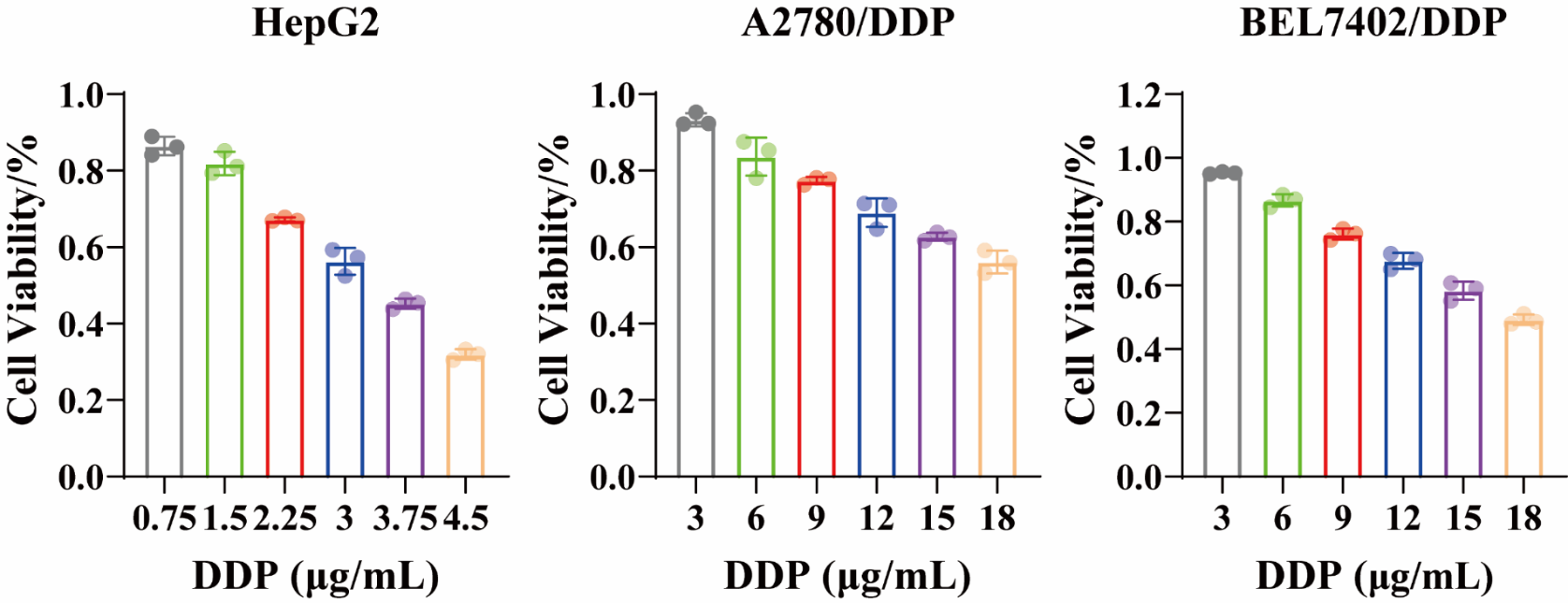


**Fig. S12.** The cell viability of different cells exposed to different concentrations of DDP for 24 h. (n ≥ 3; error bars represent SD).


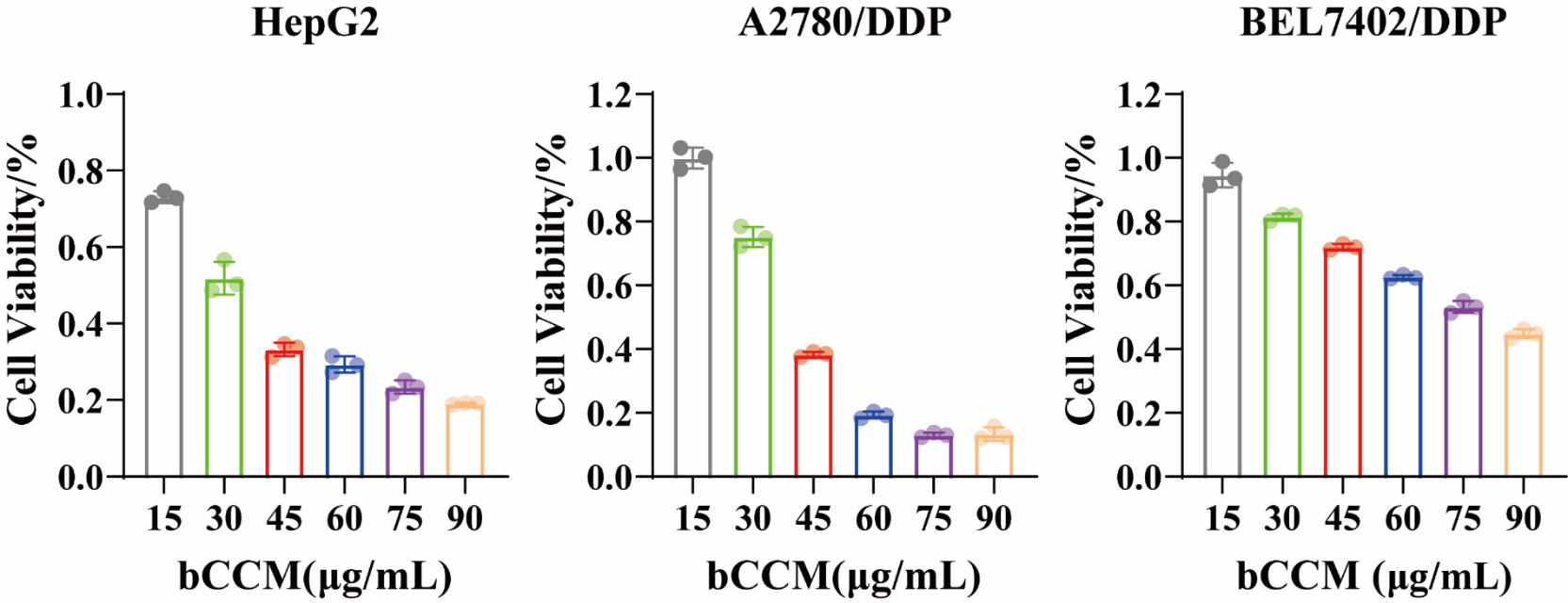


**Fig. S13.** The cell viability of different cells exposed to different concentrations of bCCM for 24 h. (n ≥ 3; error bars represent SD).

**Table. S1.** IC_50_ for different cells with different administrations (mean±SD, n=3)

| **IC_50_** | **bCCM (μg/mL)** | **DDP (μg/mL)** |
| --- | --- | --- |
| HepG2 | 29.1±0.6 | 3.4±0.1 |
| BEL7402/DDP | 80.9±1.2 | 17.8±0.4 |
| A2780/DDP | 40.2±0.6 | 19.9±0.5 |


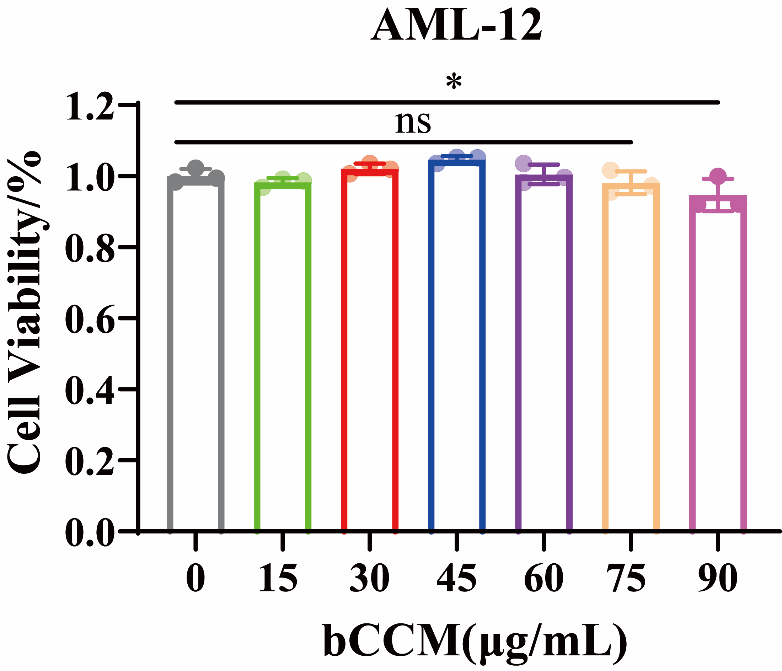


**Fig. S14.** The cell viability of AML–12 cells treated with different concentrations of bCCM for 24 h. (n ≥ 3; error bars represent SD, **p* ≤ 0.05; ** *p* ≤ 0.01; *** *p* ≤ 0.001; **** *p* ≤ 0.0001).


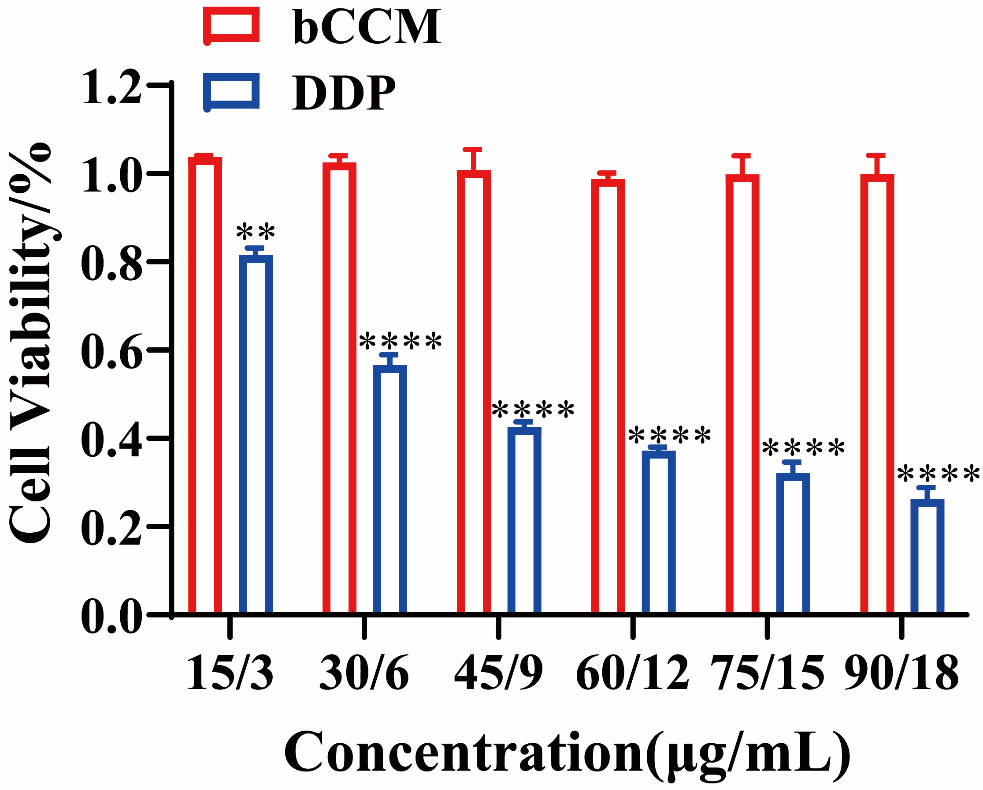


**Fig. S15.** The cell viability of LX–2 cells treated with different concentrations of bCCM and DDP for 24 h. (n ≥ 3; error bars represent SD, **p* ≤ 0.05; ** *p* ≤ 0.01; *** *p* ≤ 0.001; **** *p* ≤ 0.0001).


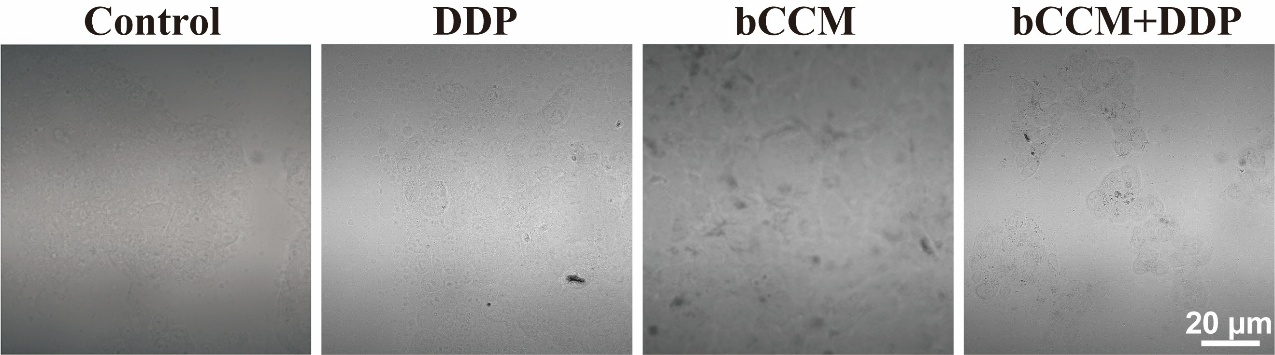


**Fig. S16.** Bright field image of BEL7402/DDP cells for Live/dead staining.


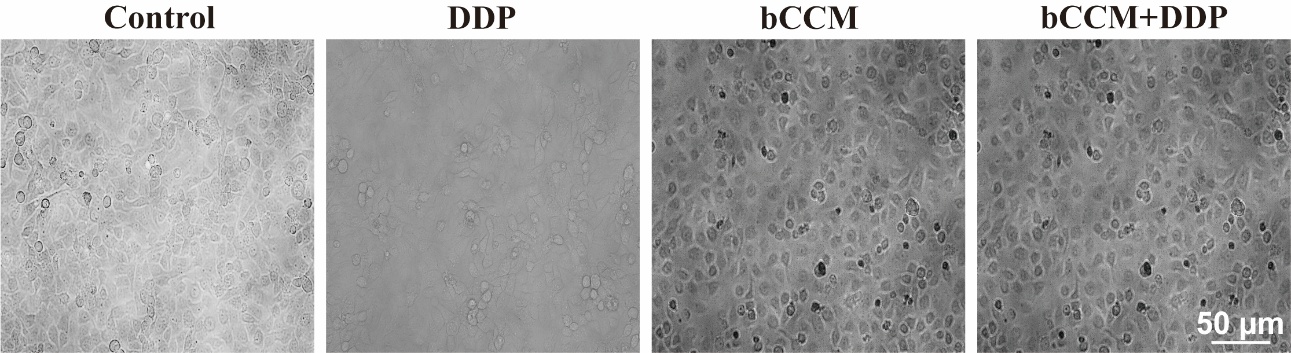


**Fig. S17.** Bright field image of BEL7402/DDP cells for necrosis and apoptosis.


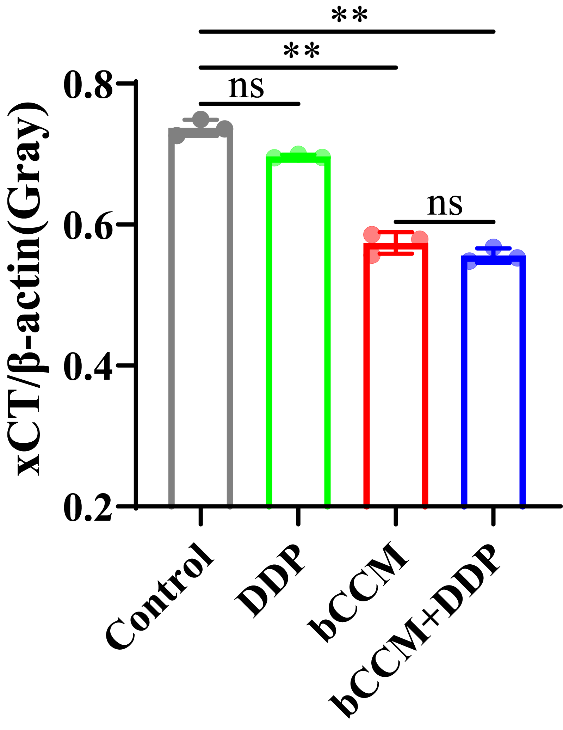


**Fig. S18.** Quantification of xCT protein in BEL7402/DDP cells treated with different groups for 24 h. (n ≥ 3; error bars represent SD, **p* ≤ 0.05; ** *p* ≤ 0.01; *** *p* ≤ 0.001; **** *p* ≤ 0.0001). [DDP]= 3 μg/mL, [bCCM] = 60 μg/mL, [bCCM + DDP] = 60 μg/mL + 1.5 μg/mL.


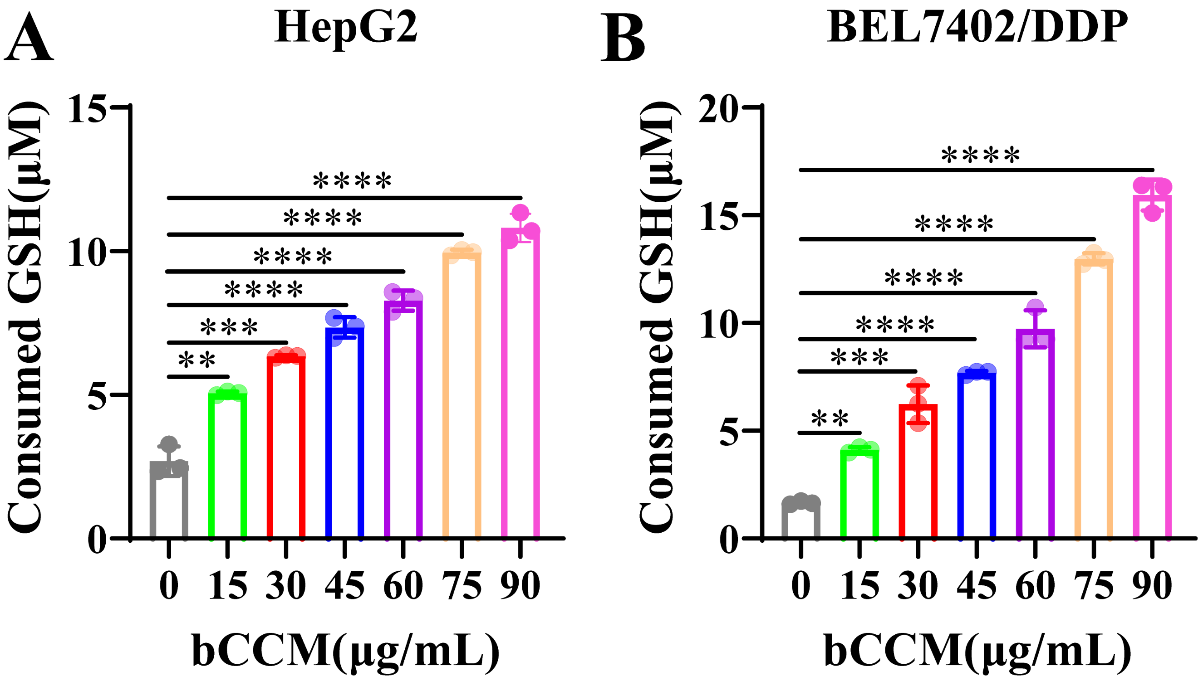


**Fig. S19.** GSH consumption capacity test of **bCCM**. (A–B) The consumed GSH of HepG2 and BEL7402/DDP cells treated with different concentrations of bCCM for 24 h. (n ≥ 3; error bars represent SD, **p* ≤ 0.05; ** *p* ≤ 0.01; *** *p* ≤ 0.001; **** *p* ≤ 0.0001).


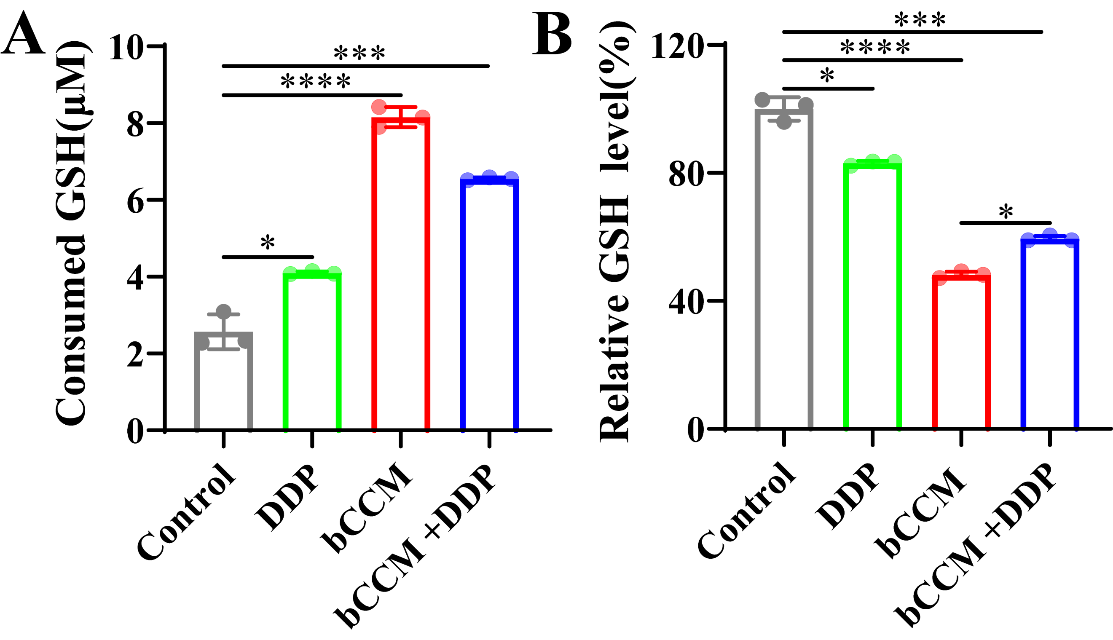


**Fig. S20.** Detection of GSH levels with different treatments on HepG2 cells. (A) The consumed GSH and (B) relative GSH level treated with different groups for 24 h. (n ≥ 3; error bars represent SD, **p* ≤ 0.05; ** *p* ≤ 0.01; *** *p* ≤ 0.001; **** *p* ≤ 0.0001). [DDP]= 3 μg/mL, [bCCM] = 60 μg/mL, [bCCM + DDP] = 60 μg/mL + 1.5 μg/mL.


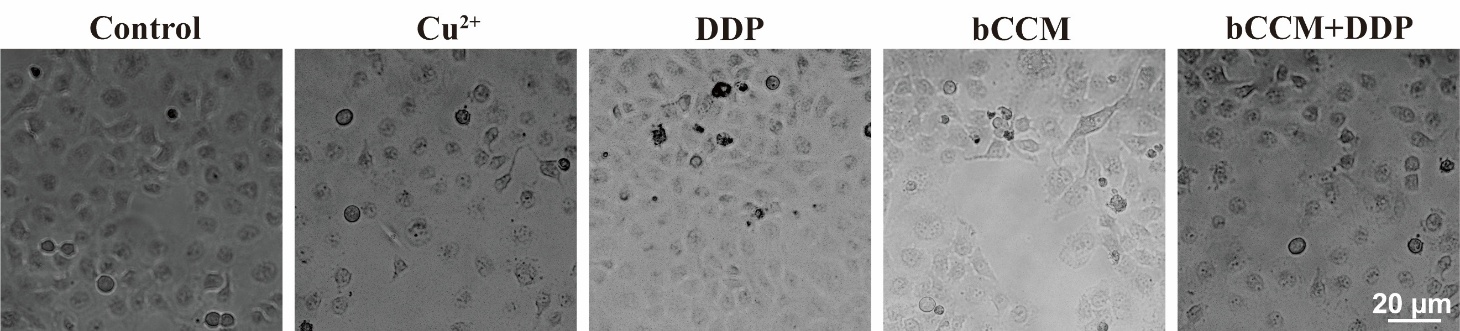


**Fig. S21.** Bright field image of BEL7402/DDP cells for intracellular copper ions.


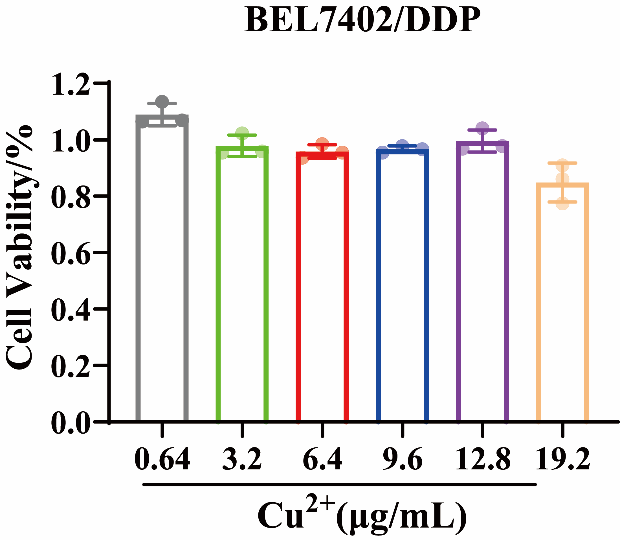


**Fig. S22.** Cytotoxicity of copper ions on BEL7402/DDP cells. The cell viability of BEL7402/DDP cells treated with different concentrations of copper ions for 24 h.


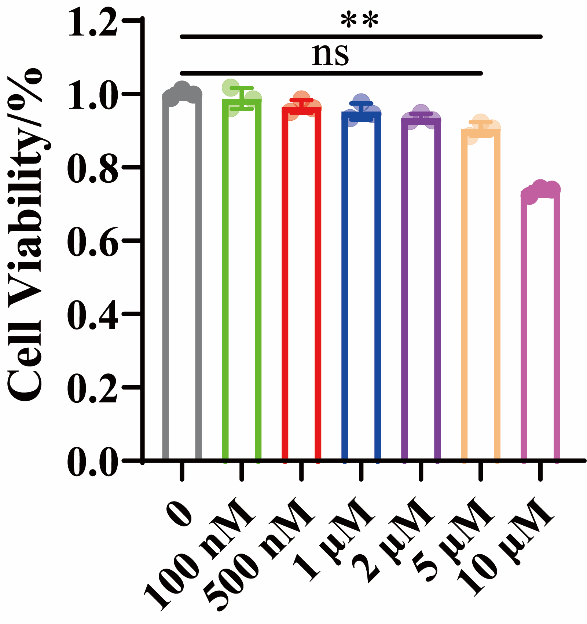


**Fig. S23.** Cytotoxicity of UK5099 on BEL7402/DDP cells. The cell viability of BEL7402/DDP cells treated with different concentrations of UK5099 for 24 h. (n ≥ 3; error bars represent SD, **p* ≤ 0.05; ** *p* ≤ 0.01; *** *p* ≤ 0.001; **** *p* ≤ 0.0001).


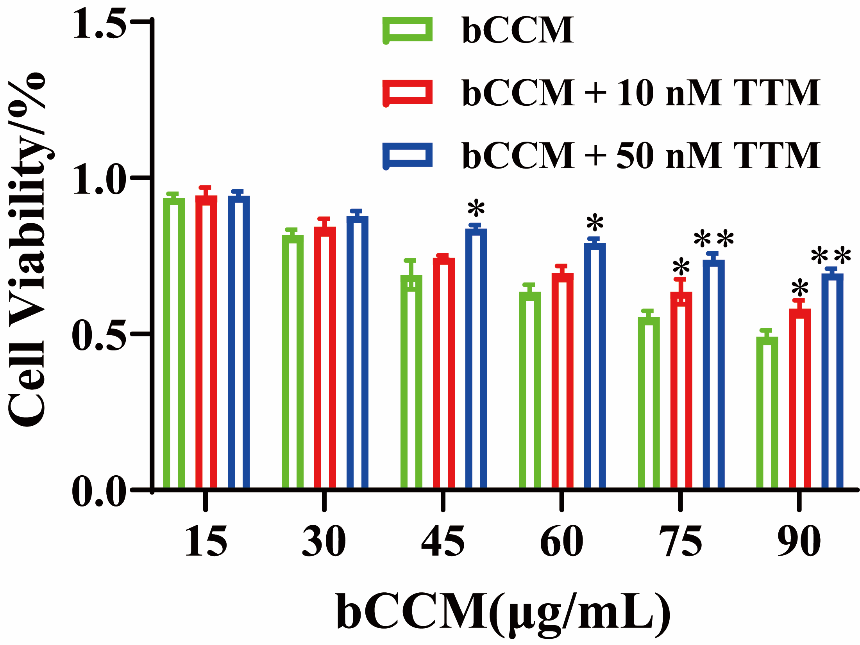


**Fig. S24.** The cell viability of BEL7402/DDP cells treated with bCCM combined with different concentrations of TTM for 24 h. (n ≥ 3; error bars represent SD, **p* ≤ 0.05; ** *p* ≤ 0.01; *** *p* ≤ 0.001; **** *p* ≤ 0.0001).


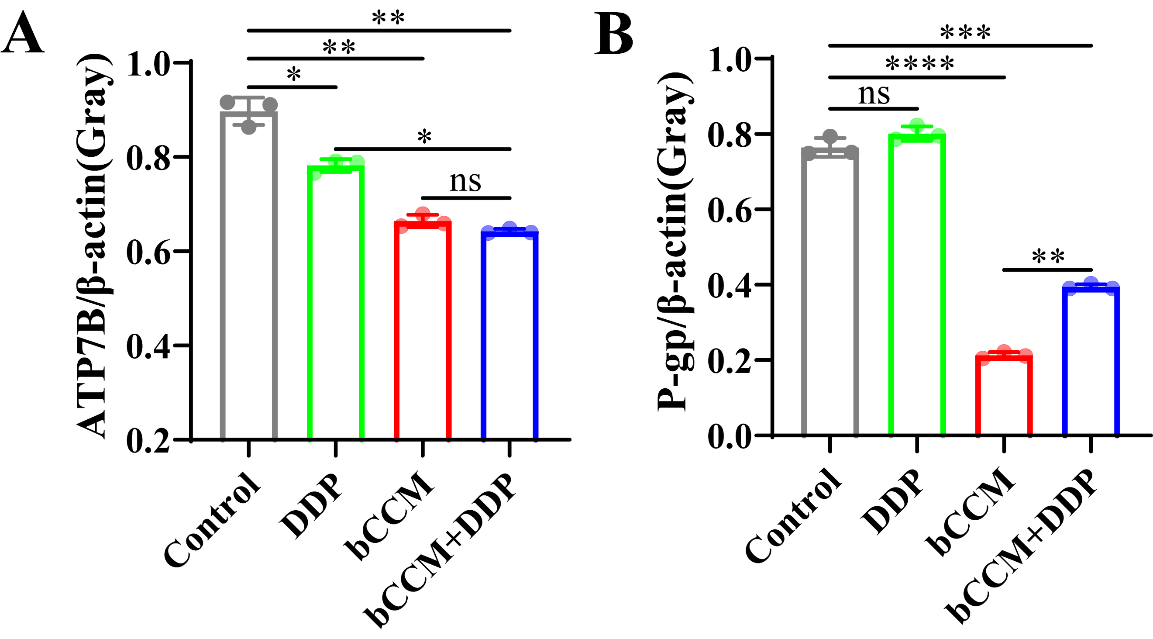


**Fig. S25.** Quantification of drug–resistant proteins (A–B) in BEL7402/DDP cells treated with different groups for 24 h. (n ≥ 3; error bars represent SD, **p* ≤ 0.05; ** *p* ≤ 0.01; *** *p* ≤ 0.001; **** *p* ≤ 0.0001). [DDP]= 3 μg/mL, [bCCM] = 60 μg/mL, [bCCM + DDP] = 60 μg/mL + 1.5 μg/mL.


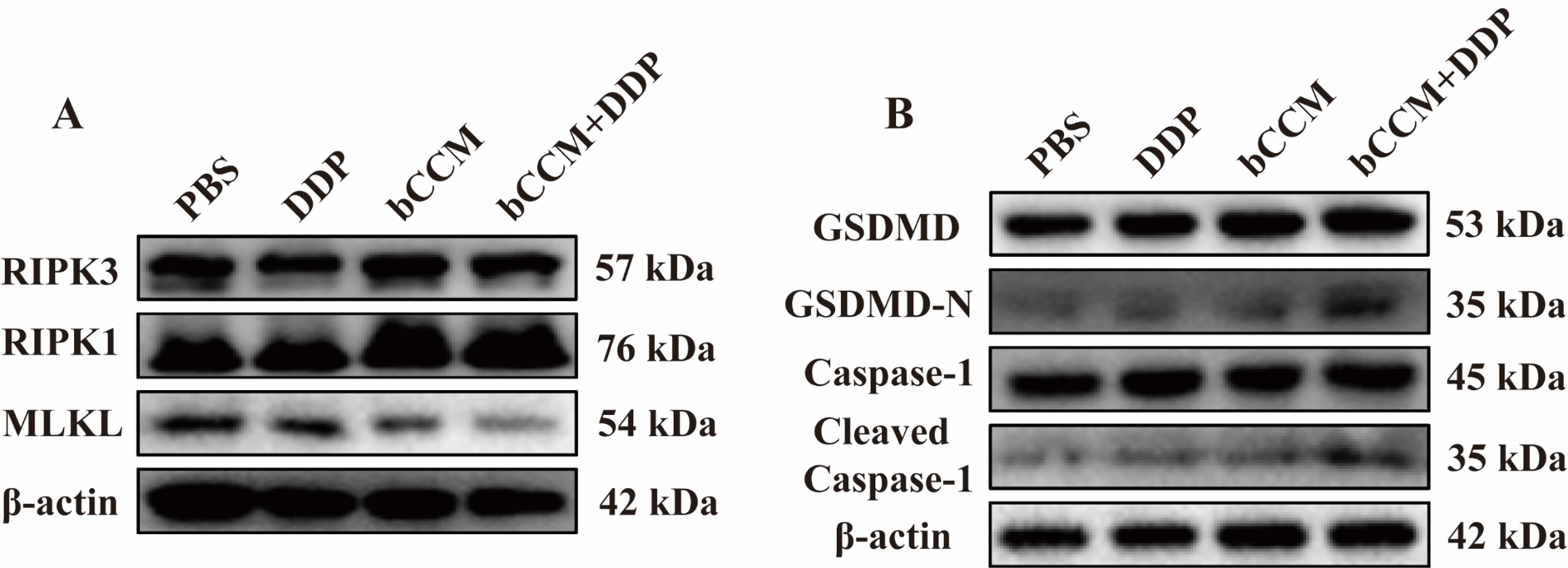


**Fig. S26.** The western blot of (A) necroptosis–related proteins and (B) pyroptosis–related proteins.


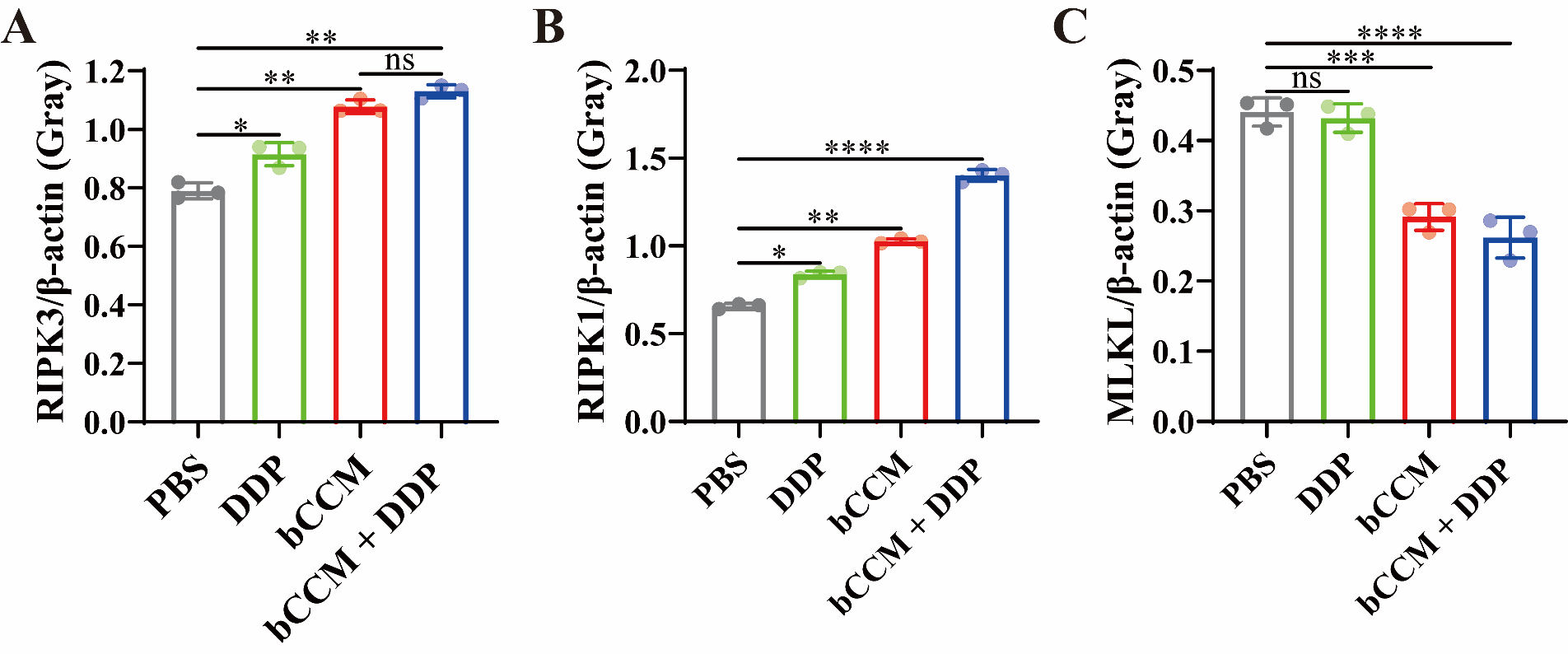


**Fig. S27.** (A–C) Quantification of necroptosis–associated proteins in BEL7402/DDP cells treated with different groups for 24 h. (n ≥ 3; error bars represent SD, **p* ≤ 0.05; ** *p* ≤ 0.01; *** *p* ≤ 0.001; **** *p* ≤ 0.0001). [DDP]= 3 μg/mL, [bCCM] = 60 μg/mL, [bCCM + DDP] = 60 μg/mL + 1.5 μg/mL.


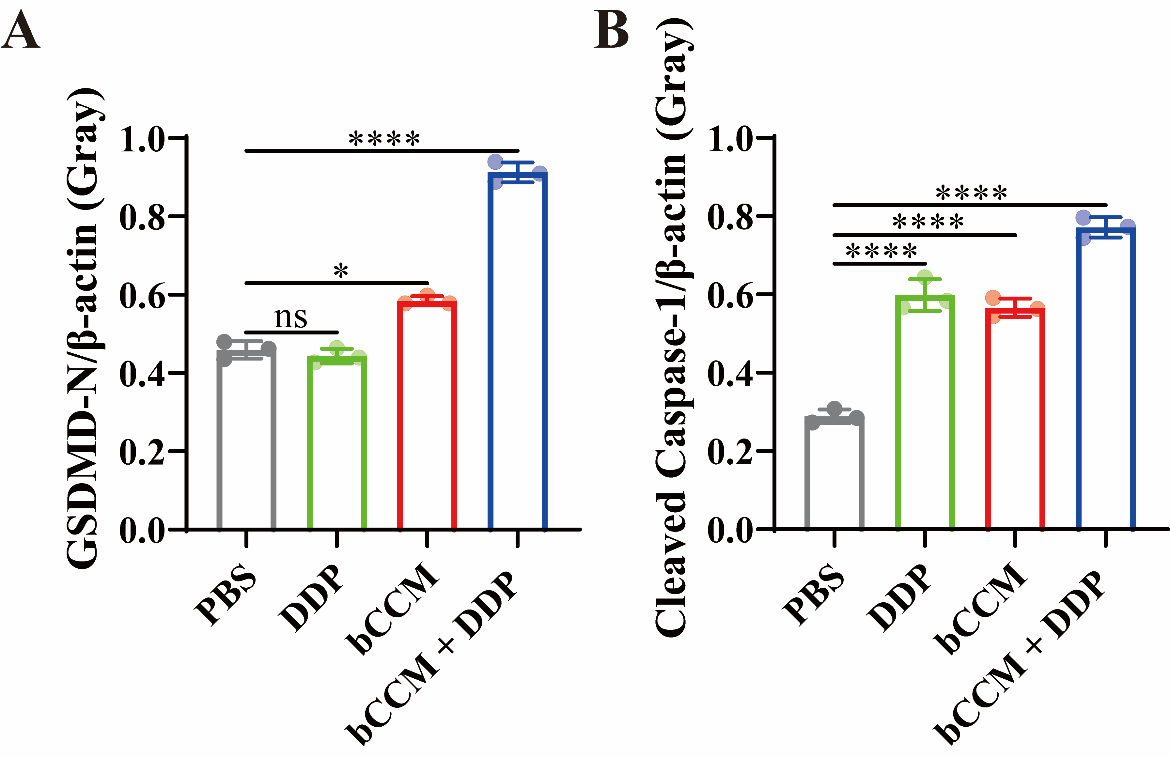


**Fig. S28.** (A–B) Quantification of pyroptosis–associated proteins in BEL7402/DDP cells treated with different groups for 24 h. (n ≥ 3; error bars represent SD, **p* ≤ 0.05; ** *p* ≤ 0.01; *** *p* ≤ 0.001; **** *p* ≤ 0.0001). [DDP]= 3 μg/mL, [bCCM] = 60 μg/mL, [bCCM + DDP] = 60 μg/mL + 1.5 μg/mL.


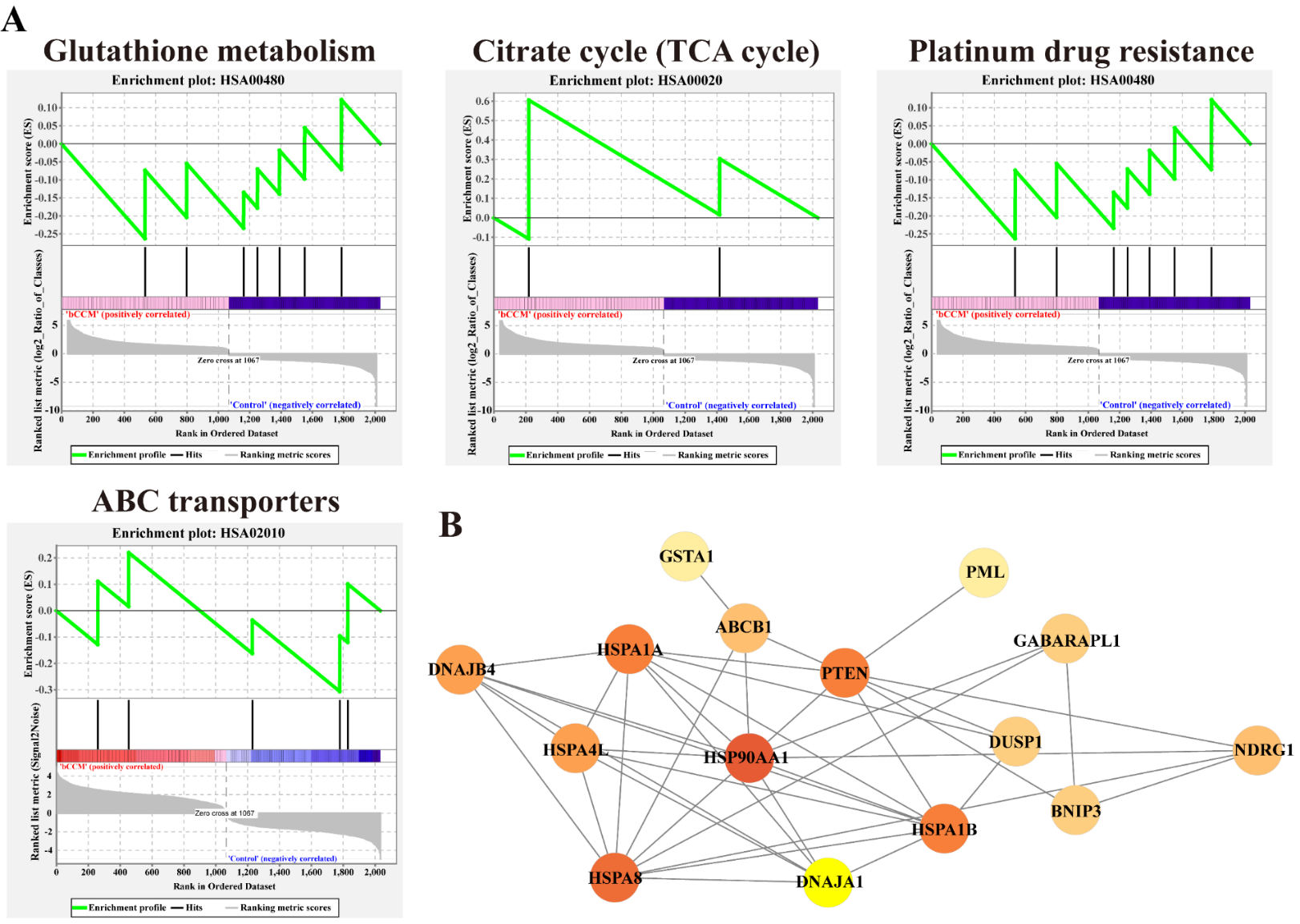


**Fig. S29.** Transcriptomics analysis of BEL7402/DDP cells post 24 h treatment with **bCCM**. (A) Gene Set Enrichment Analysis (GSEA) for Glutathione Metabolism, Citrate (TCA) cycle, Platinum drug resistance and ABC transporters pathways. (B) Protein–protein interaction (PPI) network of Cuprotosis and drug resistance.


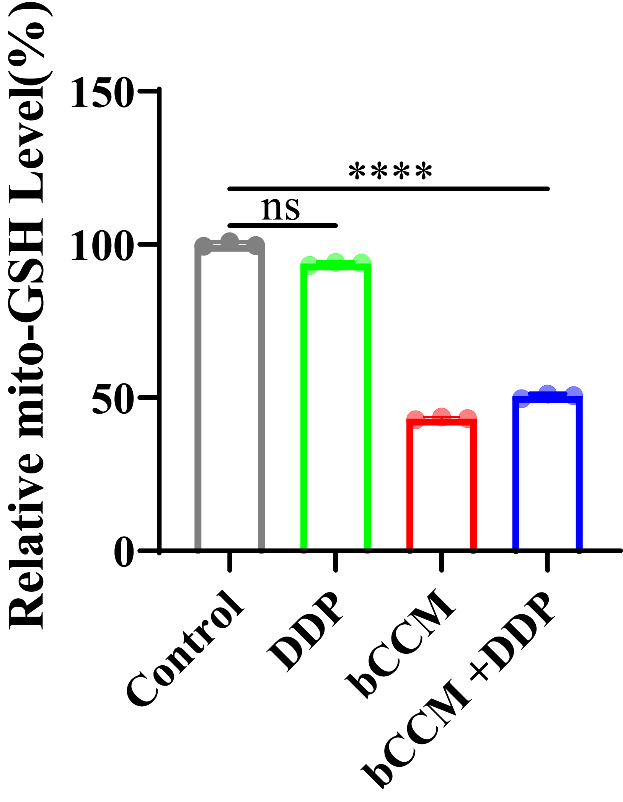


**Fig. S30.** The Relative mito–GSH level with different treatments on HepG2 cells for 24 h. (n ≥ 3; error bars represent SD, **p* ≤ 0.05; ** *p* ≤ 0.01; *** *p* ≤ 0.001; **** *p* ≤ 0.0001). [DDP]= 3 μg/mL, [bCCM] = 60 μg/mL, [bCCM + DDP] = 60 μg/mL + 1.5 μg/mL.


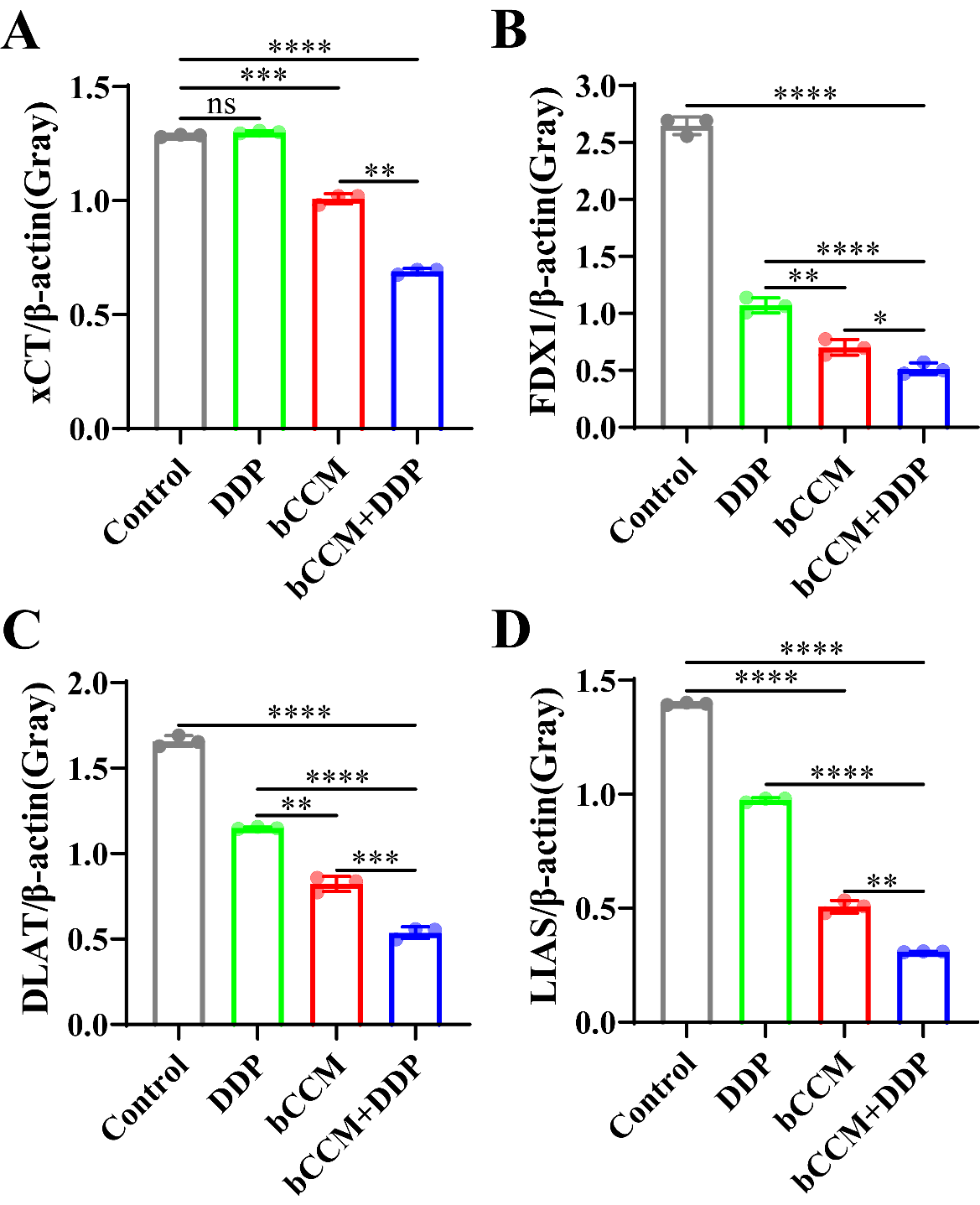


**Fig. S31.** Quantitative analysis of different proteins at the mitochondrial level. (A) Quantification of xCT in BEL7402/DDP cells treated with different groups for 24 h. (B–D) Quantification of cuproptosis–associated mito-proteins in BEL7402/DDP cells treated with different groups for 24 h. (n ≥ 3; error bars represent SD, **p* ≤ 0.05; ** *p* ≤ 0.01; *** *p* ≤ 0.001; **** *p* ≤ 0.0001). [DDP]= 3 μg/mL, [bCCM] = 60 μg/mL, [bCCM + DDP] = 60 μg/mL + 1.5 μg/mL.


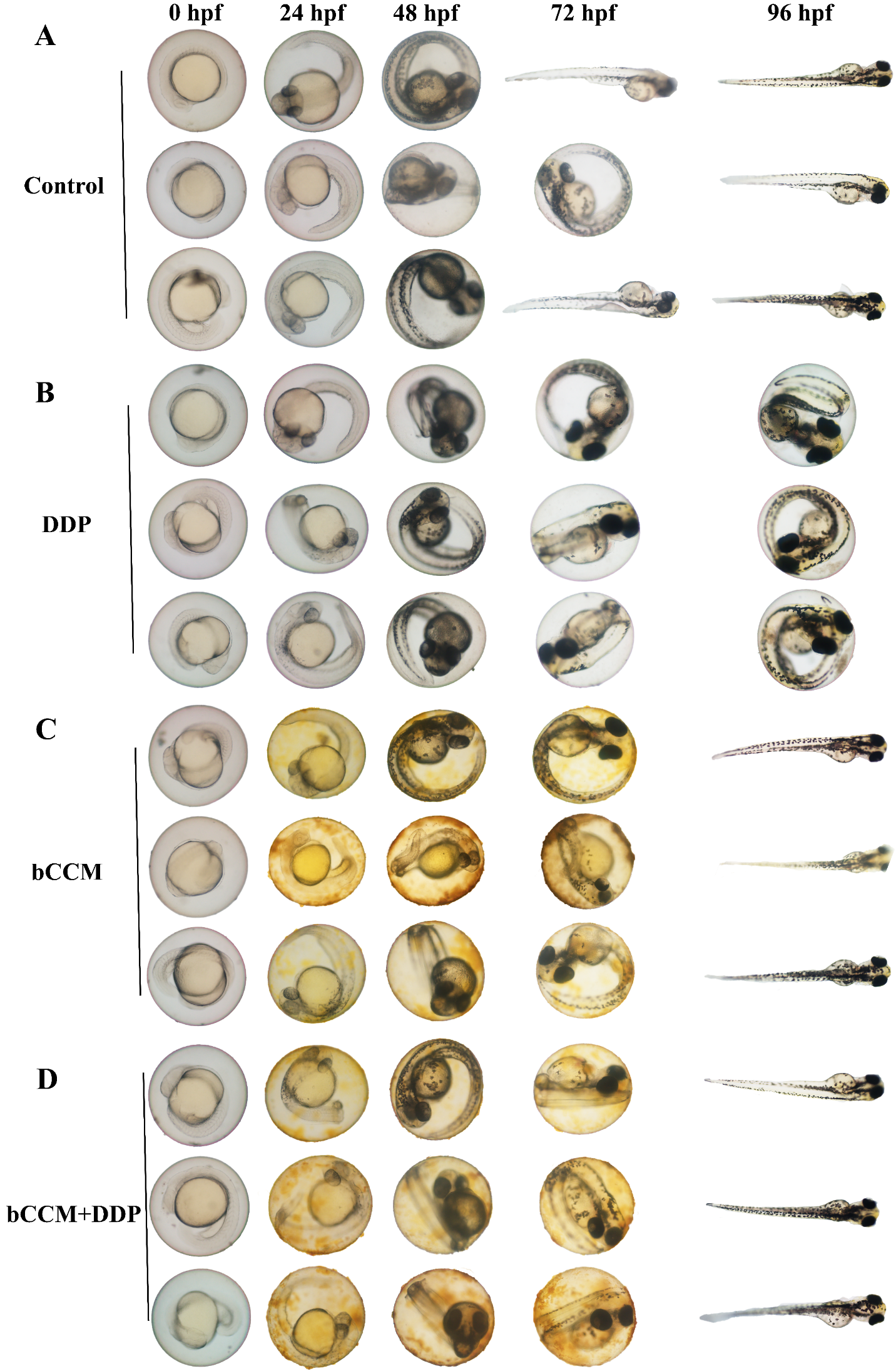


**Fig. S32.** Zebrafish toxicity assay of different groups. (A) Representative images of zebrafish from the Control group. (B) Representative images of zebrafish from the DDP group. (C) Representative images of zebrafish from the **bCCM** group. (D) Representative images of zebrafish from the bCCM+DDP group. (n=3). [DDP]= 3 μg/mL, [bCCM] = 60 μg/mL, [bCCM + DDP] = 60 μg/mL + 1.5 μg/mL.


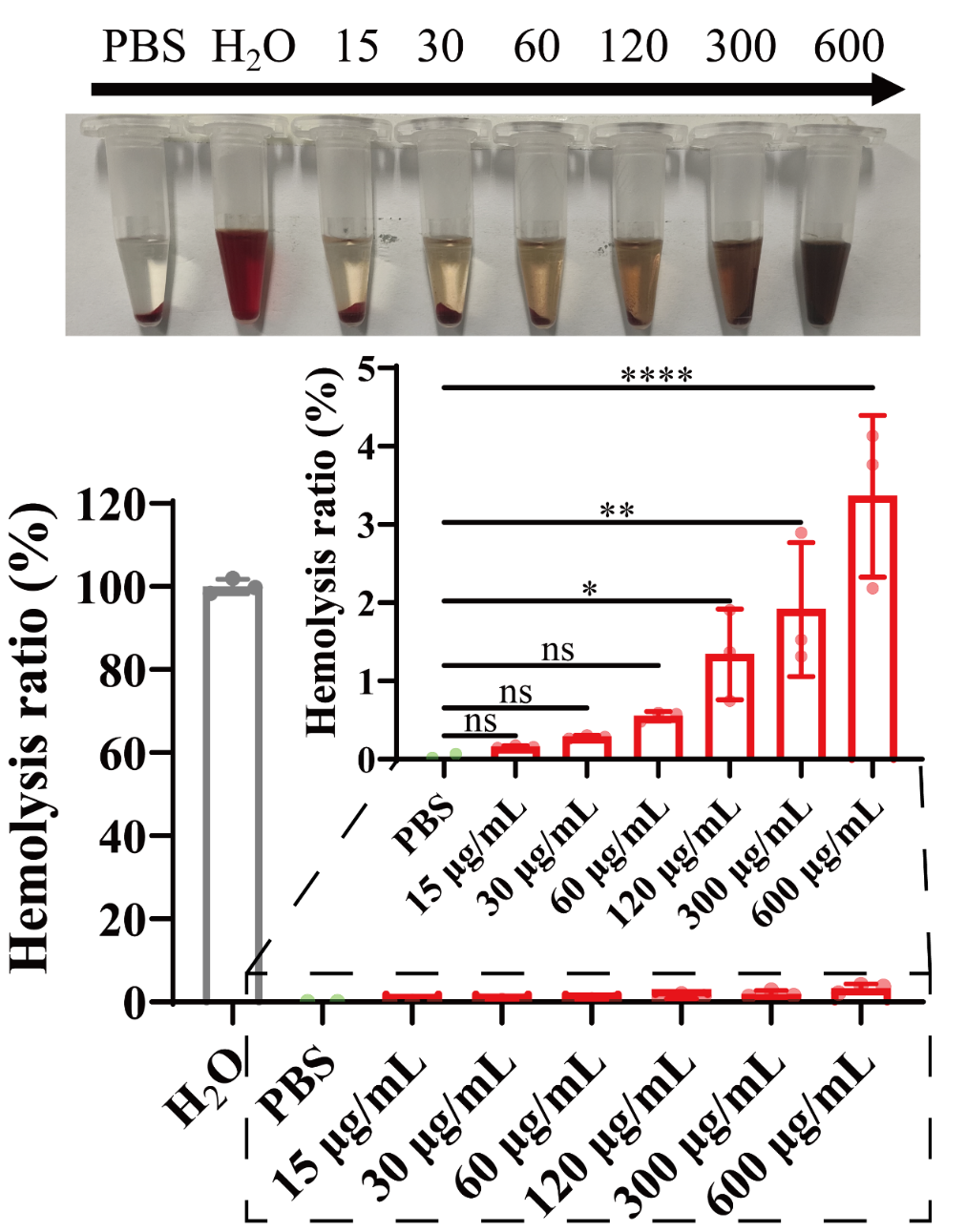


**Fig. S33.** Hemolysis images of erythrocytes incubated in H_2_O, PBS and **bCCM** and quantification of hemolysis rate. (n ≥ 3; error bars represent SD, **p* ≤ 0.05; ** *p* ≤ 0.01; *** *p* ≤ 0.001; **** *p* ≤ 0.0001).


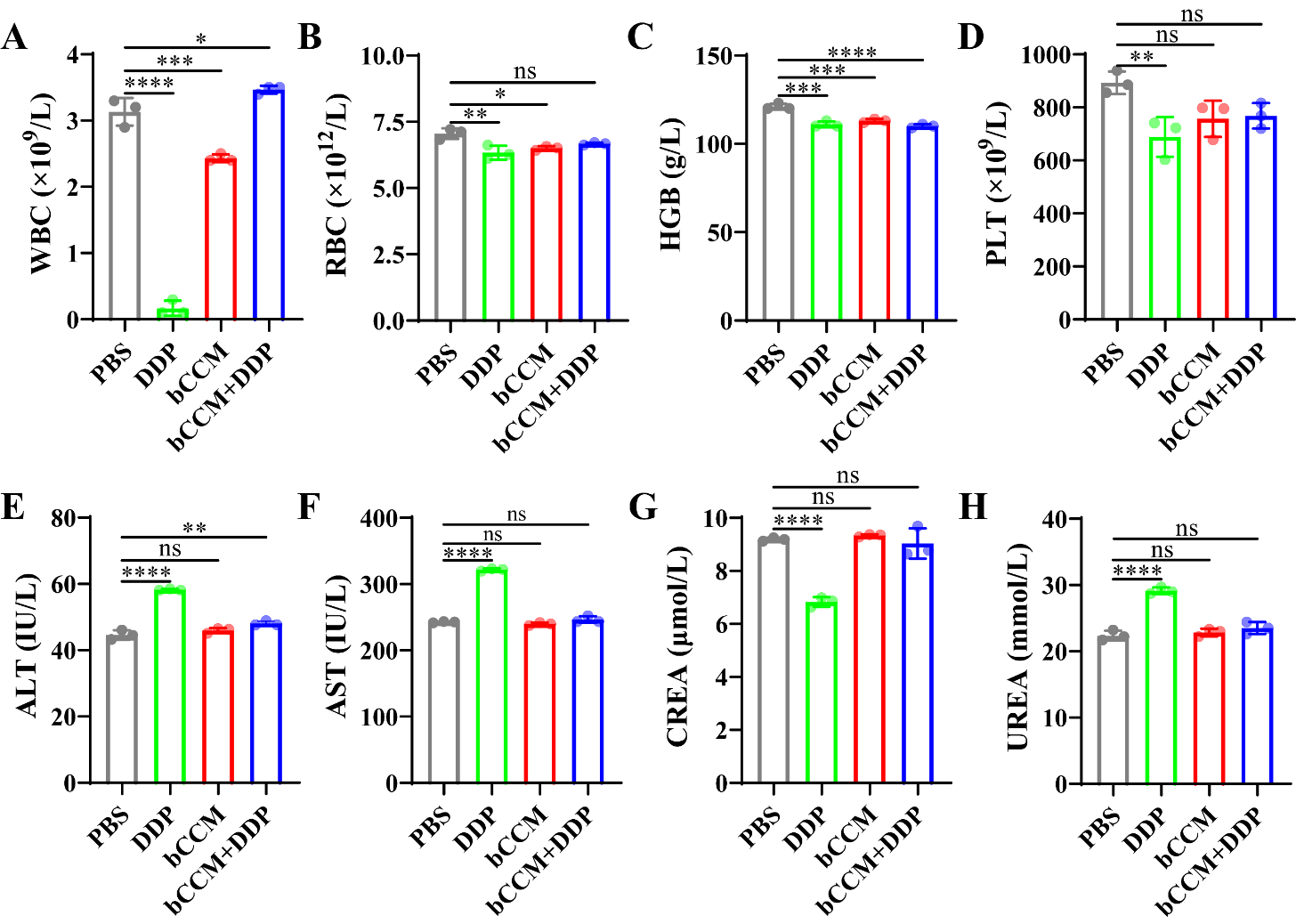


**Fig. S34.** Biosafety and biocompatitably of **bCCM**. (A) WBC: white blood cell, (B) RBC: red blood cell, (C) HGB: hemoglobin, (D) PLT: blood platelet. Liver function markers: (E) ALT: alanine aminotransferase and (F) AST: aspartate aminotransferase. Kidney function markers: (G) CREA: creatinine and (H) BUN: blood urea–nitrogen. Error bars denote the standard deviation. (n ≥ 3; error bars represent SD, **p* ≤ 0.05; ** *p* ≤ 0.01; *** *p* ≤ 0.001; **** *p* ≤ 0.0001). [DDP]= 5 mg/kg, [bCCM] = 3 mg/kg, [bCCM + DDP] = 3 mg/kg + 2.5 mg/kg.


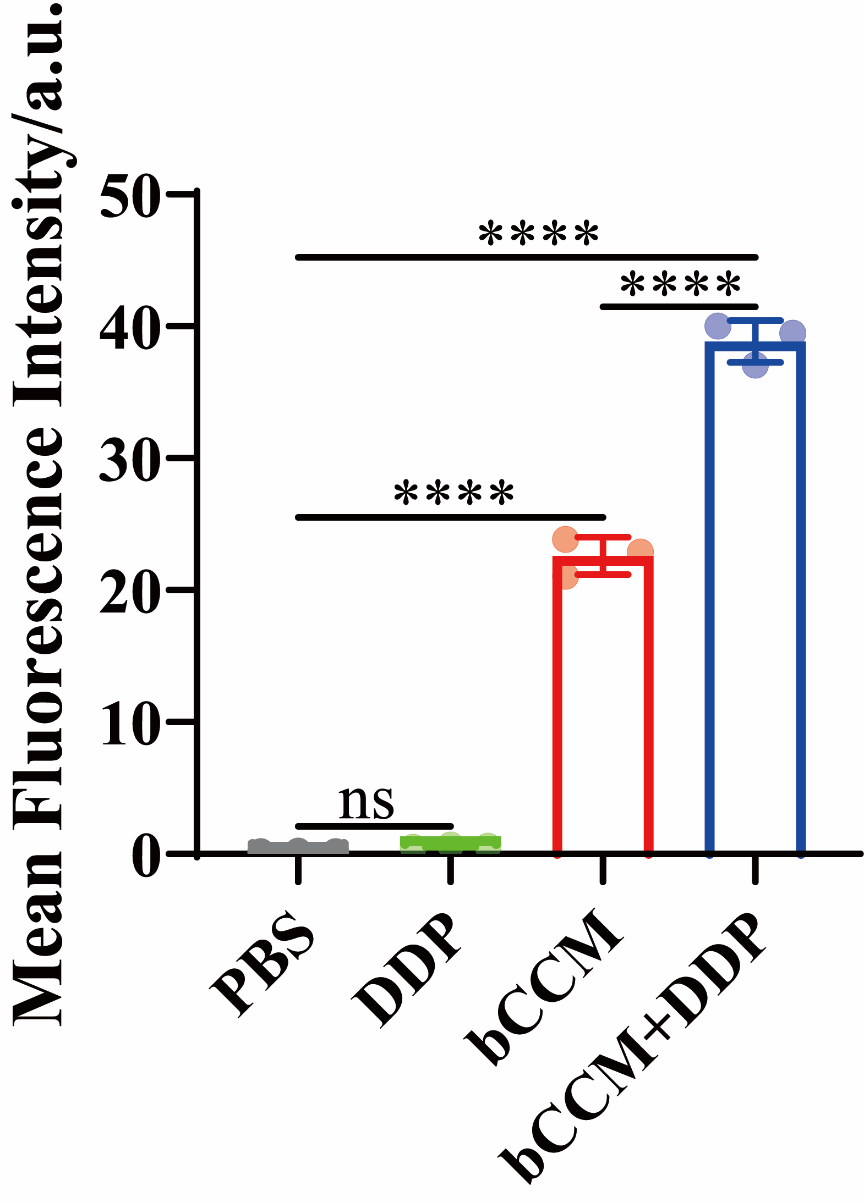


**Fig. S35.** Quantitative analysis of mean fluorescence by Tunel assay. (n ≥ 3; error bars represent SD, **p* ≤ 0.05; ** *p* ≤ 0.01; *** *p* ≤ 0.001; **** *p* ≤ 0.0001). [DDP]= 5 mg/kg, [bCCM] = 3 mg/kg, [bCCM + DDP] = 3 mg/kg + 2.5 mg/kg.


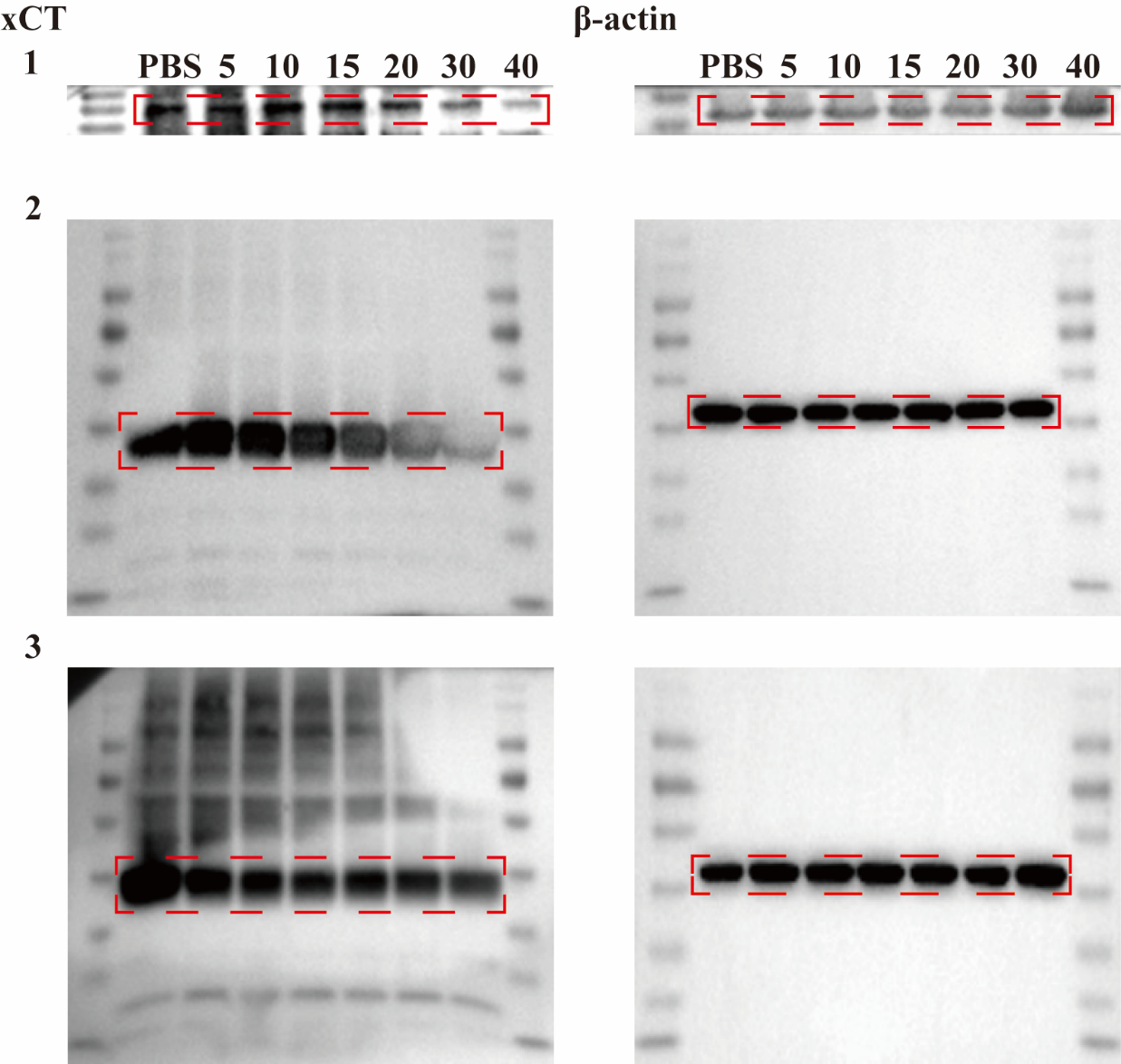


**Fig. S36.** The original blots of Fig. 1D.


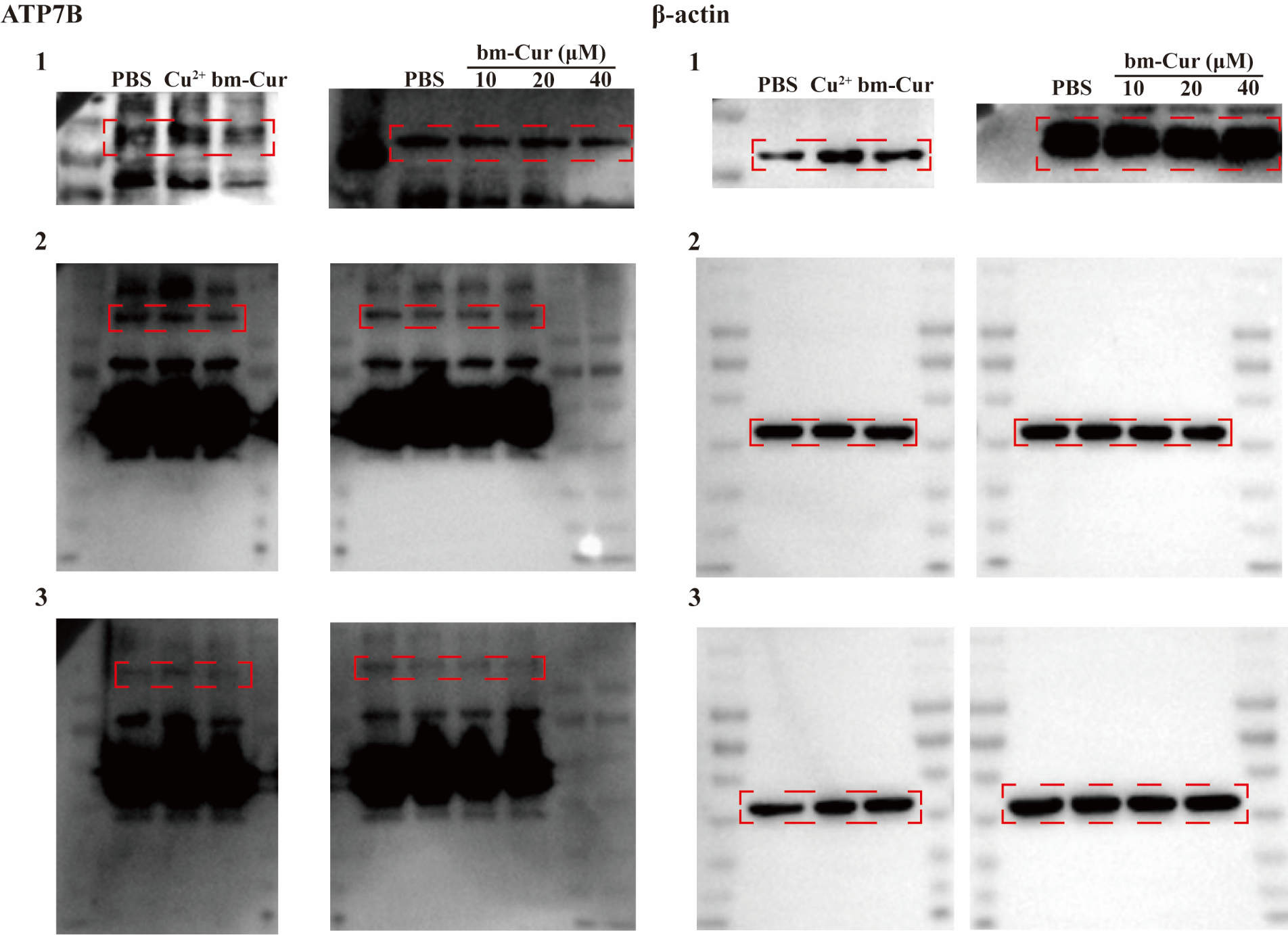


**Fig. S37.** The original blots of Fig. 1E.


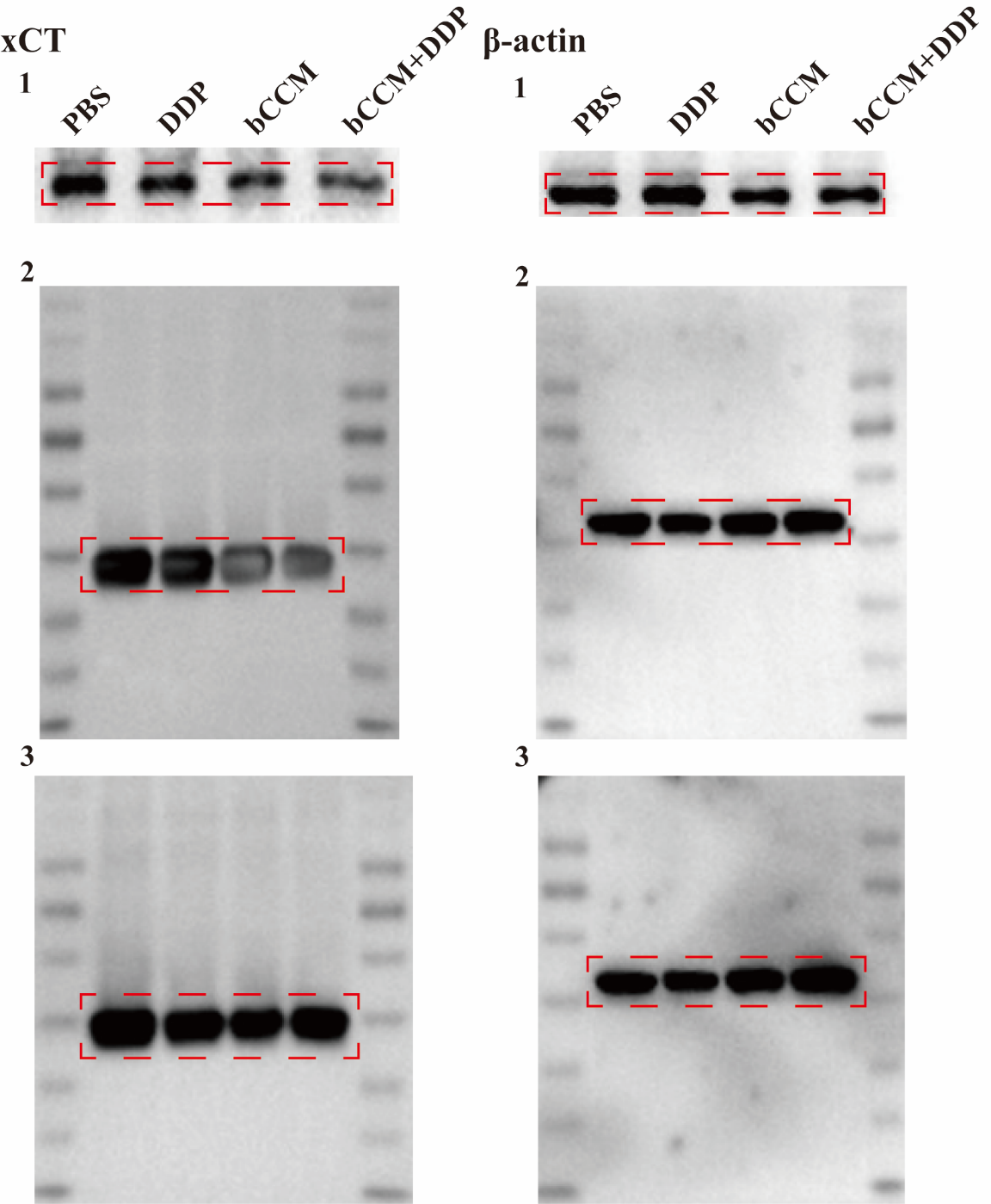


**Fig. S38.** The original blots of Fig. 2F.


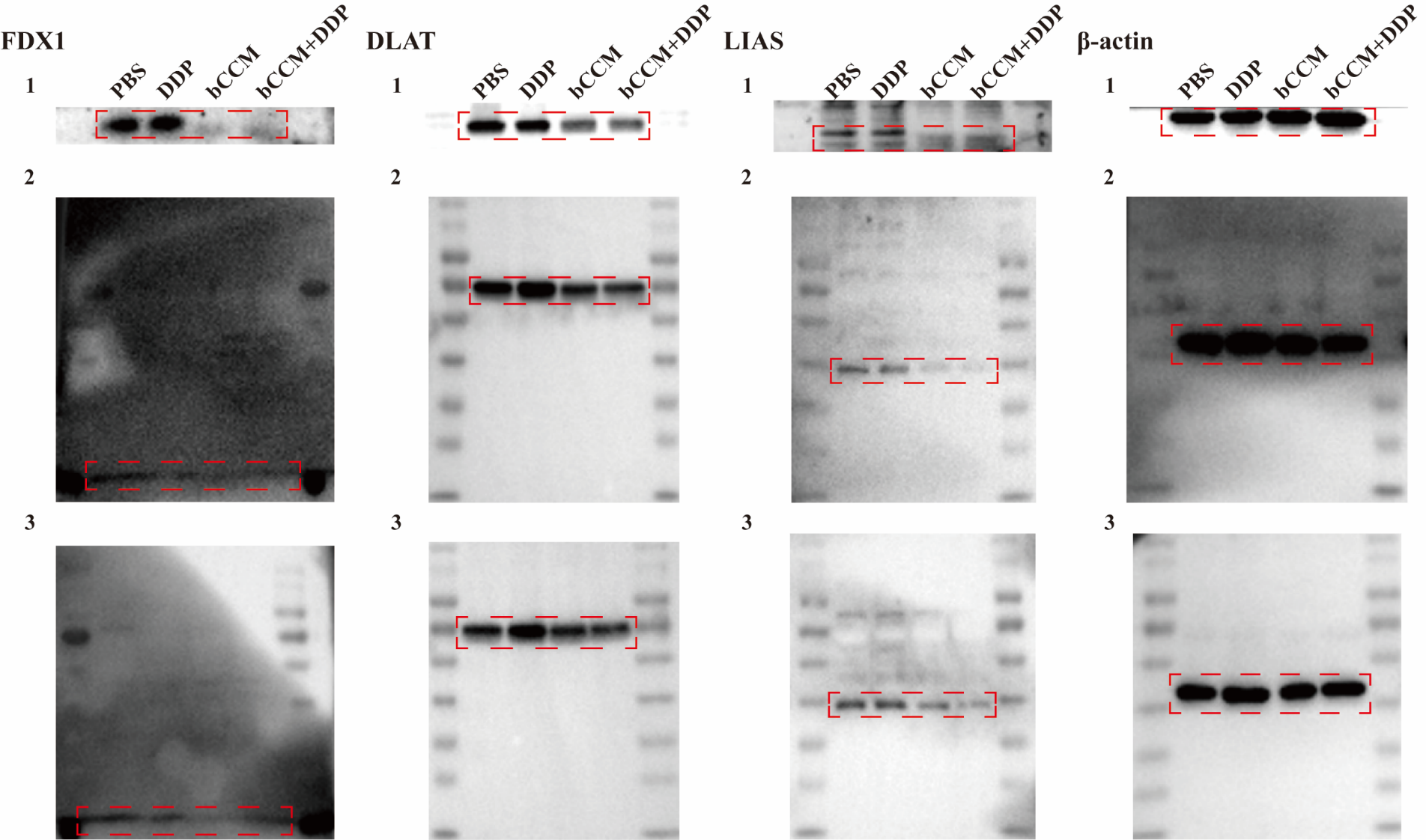


**Fig. S39.** The original blots of Fig. 3G.


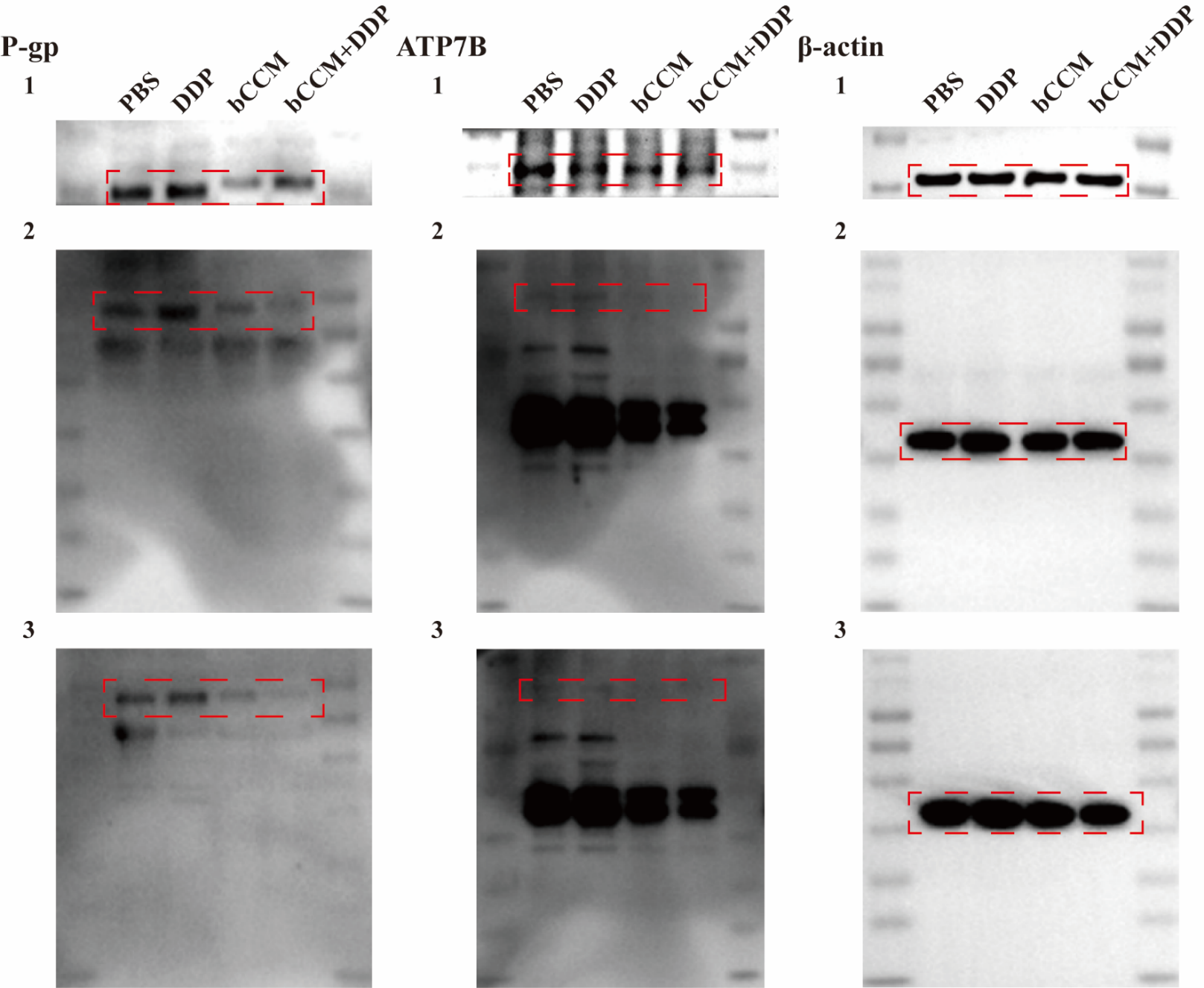


**Fig. S40.** The original blots of Fig. 3K.


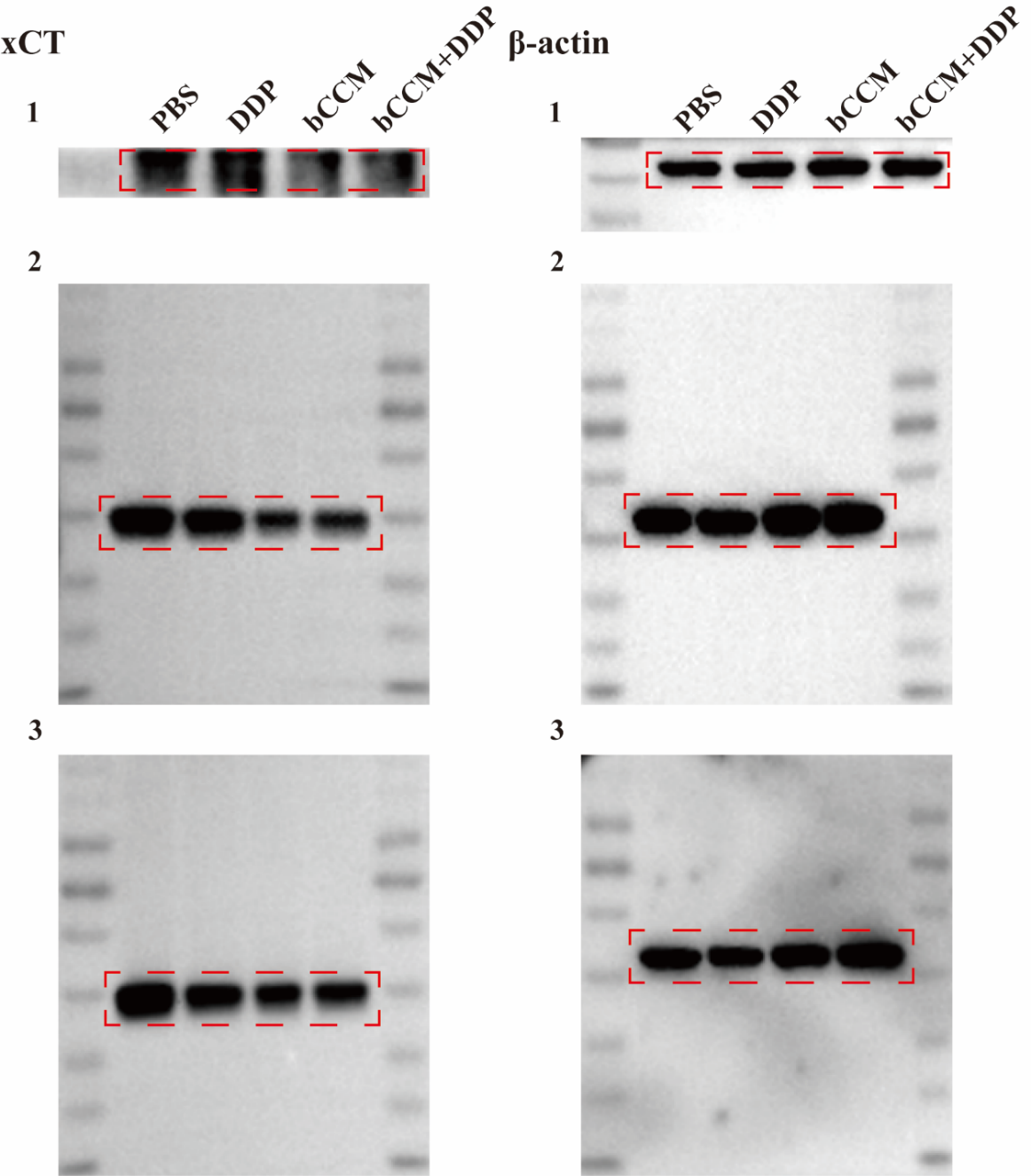


**Fig. S41.** The original blots of Fig. 4D.


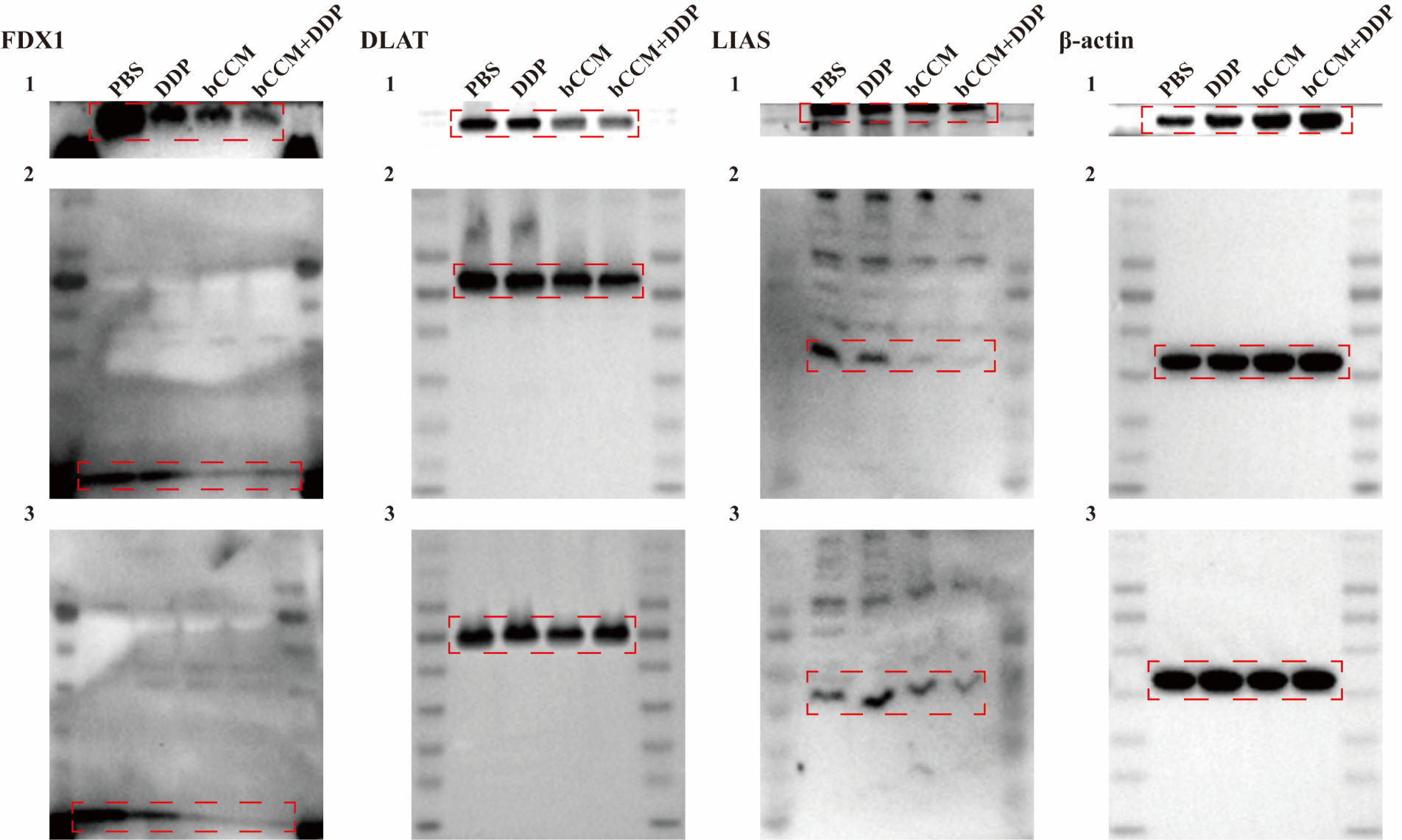


**Fig. S42.** The original blots of Fig. 4G.


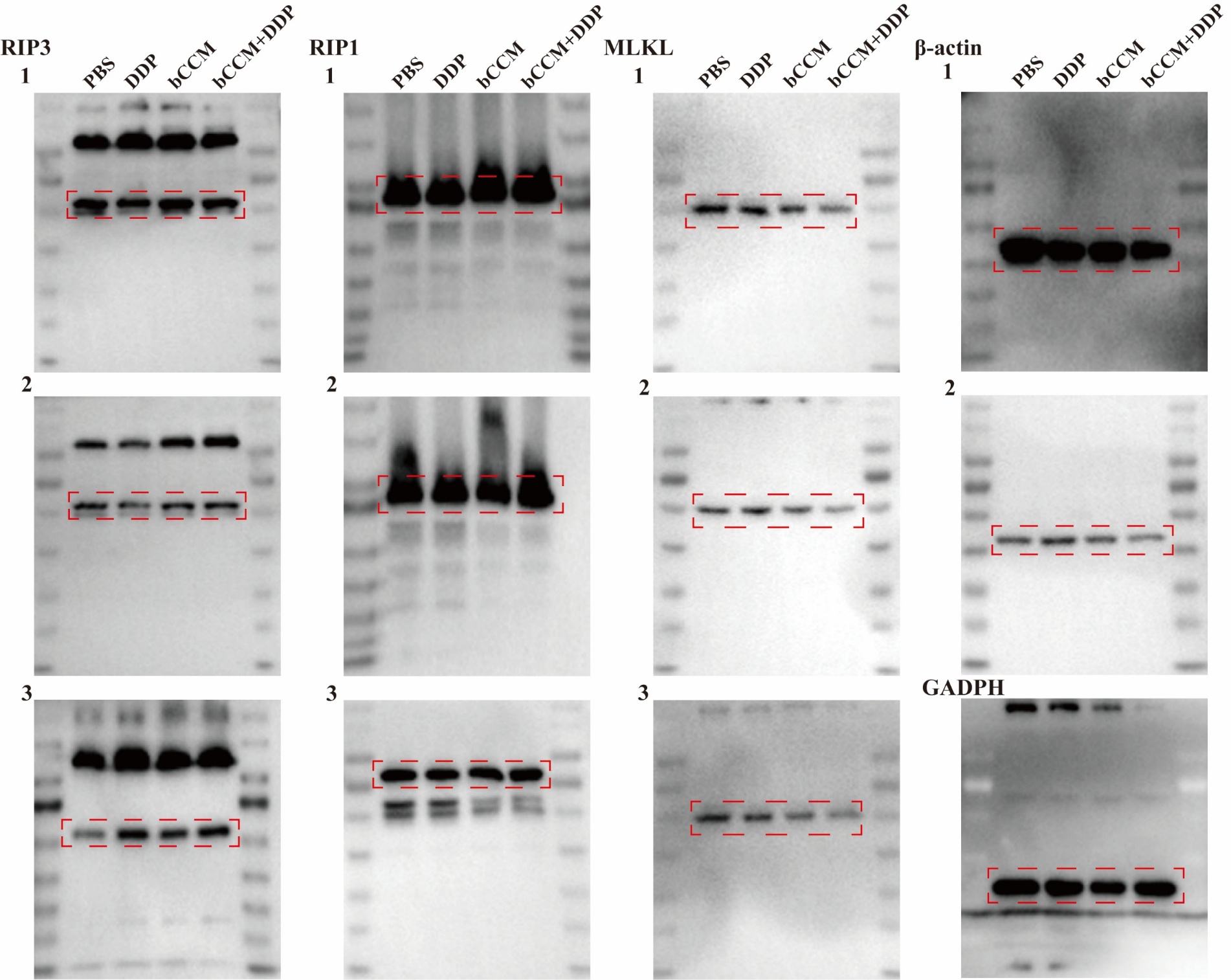
**Fig. S43.** The original blots of necroptosis–associated protein (Fig. S25A).


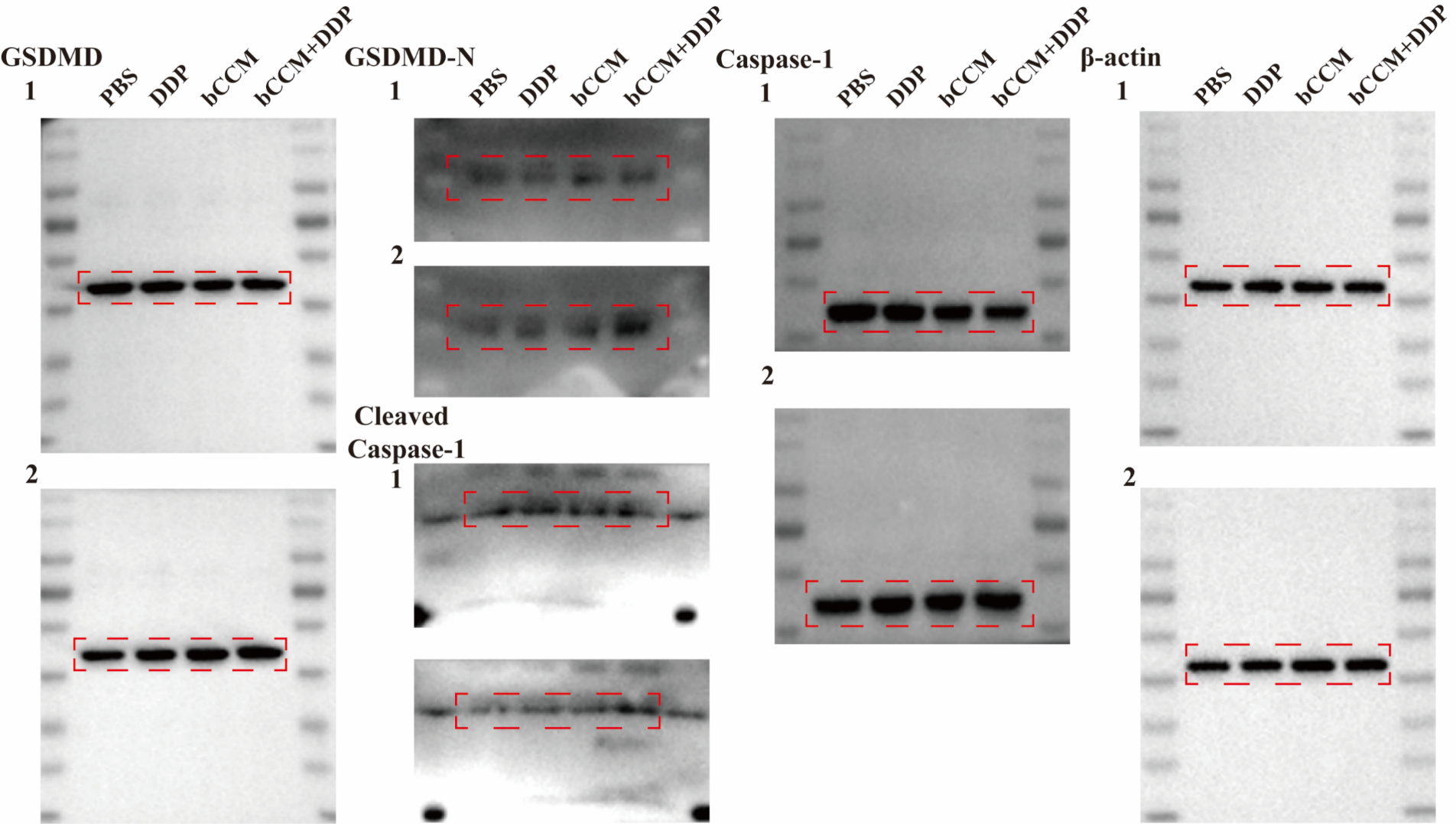


**Fig. S44.** The original blots of pyroptosis–related proteins (Fig. S25B).
